# Supplementary material for: Nup358 restricts ER-mitochondria connectivity by modulating mTORC2/Akt/GSK3β signalling
Source: EMBO Rep. 2024 Jul 18;25(10):14. doi: 10.1038/s44319-024-00204-8 (PMC11466962; doi:10.1038/s44319-024-00204-8)
Supplement: Supplementary file 1 — Appendix [file 44319_2024_204_MOESM1_ESM.pdf]

Nup358 restricts ER-mitochondria connectivity by modulating mTORC2/Akt/GSK3β signalling

Misha Kalarikkal, Rimpi Saikia, Lizanne Oliveira, Yashashree Bhorkar, Akshay Lonare, Pallavi Varshney, Prathamesh Dhamale, Amitabha Majumdar and Jomon Joseph*

National Centre for Cell Science, S.P. Pune University Campus, Pune - 411007, India

Appendix Figure	Title	Page No.
1	Specificity of *in situ* PLA with VAPB and PTPIP51 antibodies	1
2	Nup358 depletion does not affect the AL integrity	2
3	Details of Nup358 KO HeLa cell line	3
4	Nup358 depletion does not affect mitochondrial content	4
5	Nup358 restricts Epidermal Growth Factor (EGF)-stimulated activation of mTORC2	5
6	Insulin signalling does not affect the levels of proteins involved in ERMCS formation and/or regulation	6
7	Depletion of VAPB and PTPIP51 does not affect the levels of mTORC2 complex components	7

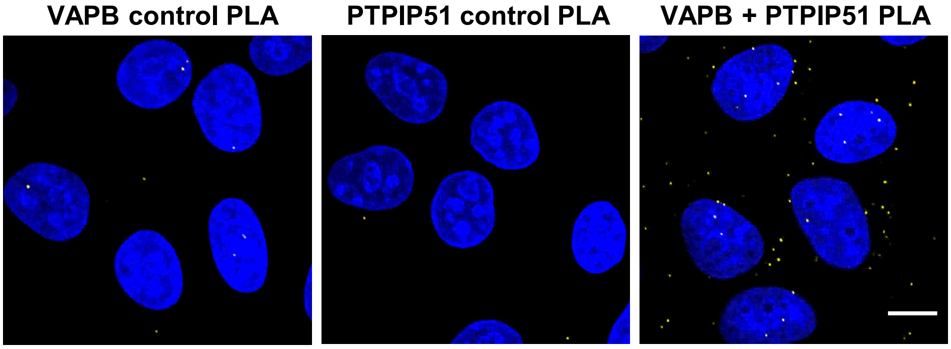

Appendix Fig S1. Specificity of *in situ* PLA with VAPB and PTPIP51 antibodies

HeLa cells were either incubated with antibodies against VAPB (control PLA) or PTPIP51 (control PLA) or both (VAPB+PTPIP51 PLA) and proceeded with PLA. DNA was stained with Hoechst 33342 (blue). Specific PLA puncta (red) were apparent when incubated with both antibodies (VAPB+PTPIP51) as compared to single antibody control. Scale bar, 10 µm.

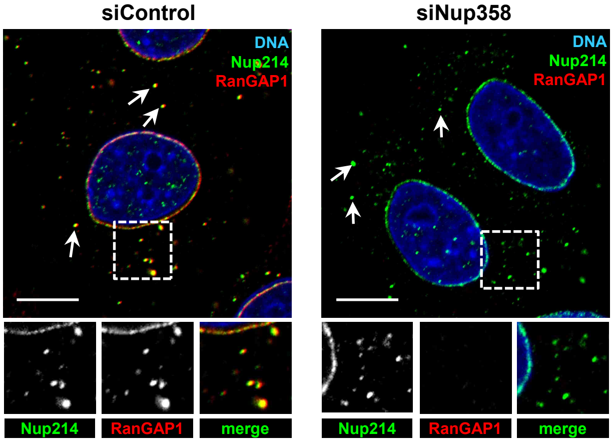

Appendix Figure S2. Nup358 depletion does not affect the AL integrity

Depletion of Nup358 does not affect AL integrity. HeLa cells were treated with siControl or siNup358 and were immunostained for Nup214 (as an AL marker, green) and RanGAP1 (red). Note that Nup214 positive AL (arrows) puncta largely remained unaffected. DNA was stained with Hoechst 33342 (blue). Scale bar, 10 µm.

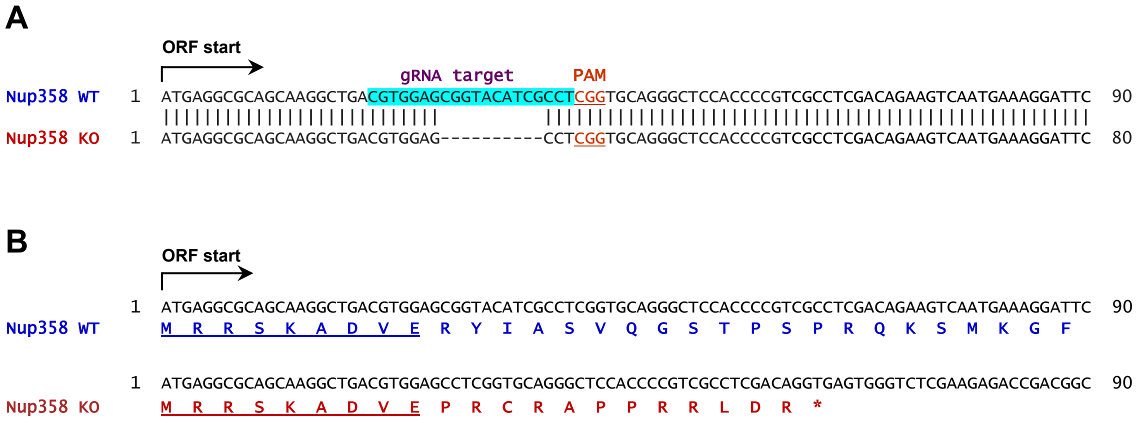

Appendix Figure S3. Details of Nup358 KO HeLa cell line

A. HeLa cells were transfected with a construct encoding Cas9 protein, and a guide(g) RNA targeted against Nup358 [highlighted in blue in Nup358 wild type (WT) sequence]. PAM sequence in Nup358 is also labelled. The Nup358 knockout (KO) line that was used in this study was heterozygous. It had one wild type allele and the other allele had deletions (indicated as dashed lines in Nup358 KO sequence).

B. The KO cell line has a 10-nucleotide deletion in one of the alleles, thus rendering it incapable of producing full length Nup358, but predicted to produce a 21-amino acid peptide as indicated in Nup358 KO sequence. Common sequence present in the WT and peptide derived from the KO allele are underlined. * indicates stop codon. ORF; open reading frame.

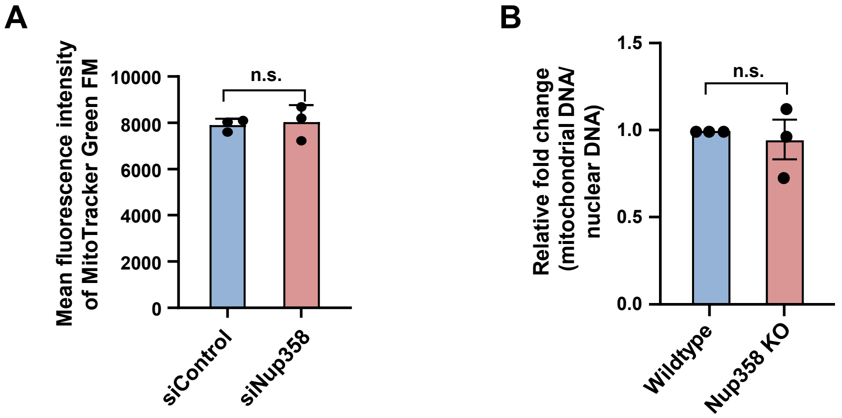

Appendix Figure S4. Nup358 depletion does not affect mitochondrial content

A. HeLa cells were treated with control (siControl) or Nup358 (siNup358) siRNA and stained for mitochondria using the MitoTracker Green FM dye. The graph represents mean fluorescence intensity of MitoTracker Green FM dye, analysed by FACS, in siControl and siNup358 cells (*n* = 3 independent experiments). Data are mean ± SEM, unpaired Student's *t* test, n.s., not significant (*P*>0.05).

B. Quantitative real time (qRT) PCR for mtDNA (cytochrome b) was performed in HeLa wild-type and Nup358 knockout (KO) cells. The nuclear gene ribosomal protein large subunit (RPL) was used for normalization. Graph represents normalized mean values (*n* = 3 independent experiments). Data are mean ± SEM, unpaired Student's *t* test, n.s., not significant (*P*>0.05).

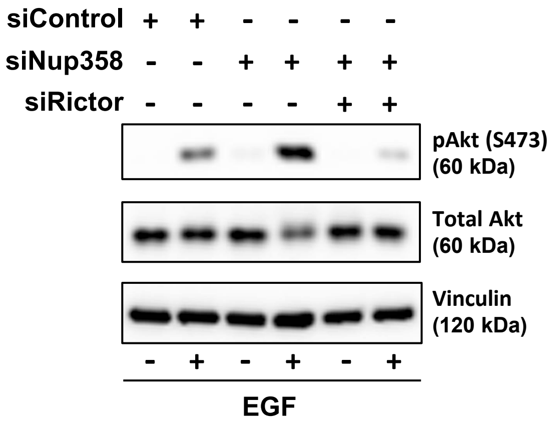

Appendix Figure S5. Nup358 restricts Epidermal Growth Factor (EGF)-stimulated activation of mTORC2

HeLa cells, transfected with indicated siRNAs, were serum starved for 3 h. Then the cells treated with (+) or without (-) epidermal growth factor (EGF, 20 ng/ml) for 10 min were analyzed for mTORC2/Akt activation by western blotting. Vinculin was used as loading control.

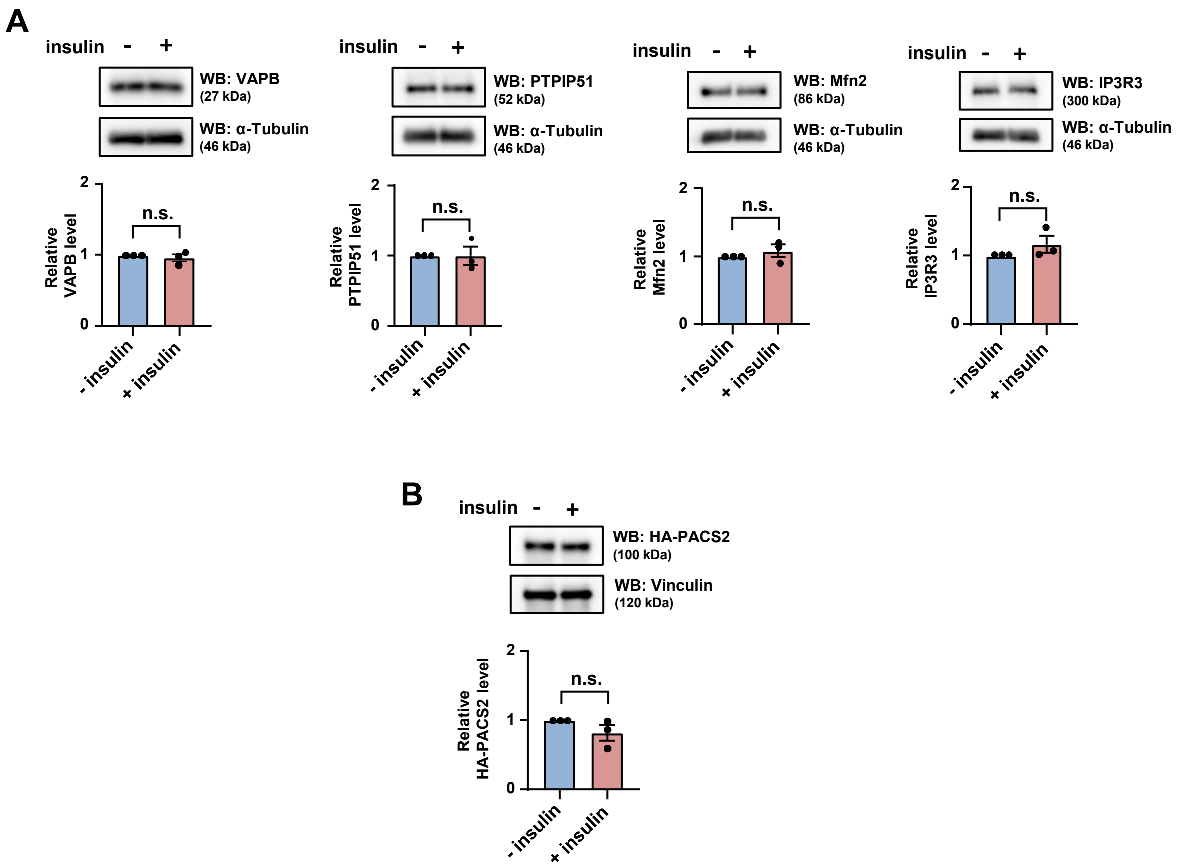

Appendix Figure S6. Insulin signalling does not affect the levels of proteins involved in ERMCS formation or regulation

A. HeLa cells were serum starved for 3 h. Then the cells treated with (+) or without (-) insulin (1 nM for 10 min) and analyzed for the levels of VAPB, PTPIP51, Mfn2 and IP3R3 as indicated. Levels of indicated proteins were quantitated, in relation to the loading control Vinculin from 3 experiments. Data are mean ± SEM, unpaired Student's *t* test, n.s., not significant (*P*>0.05).

B. HeLa cells were transfected with HA-PACS2 for 36 h, and serum starved for 3 h before insulin treatment for 20 min. Vinculin was used as loading control. Levels of indicated proteins were quantitated, in relation to the loading control Vinculin from 3 experiments. Data are mean ± SEM, unpaired Student's *t* test, n.s., not significant (*P*>0.05).

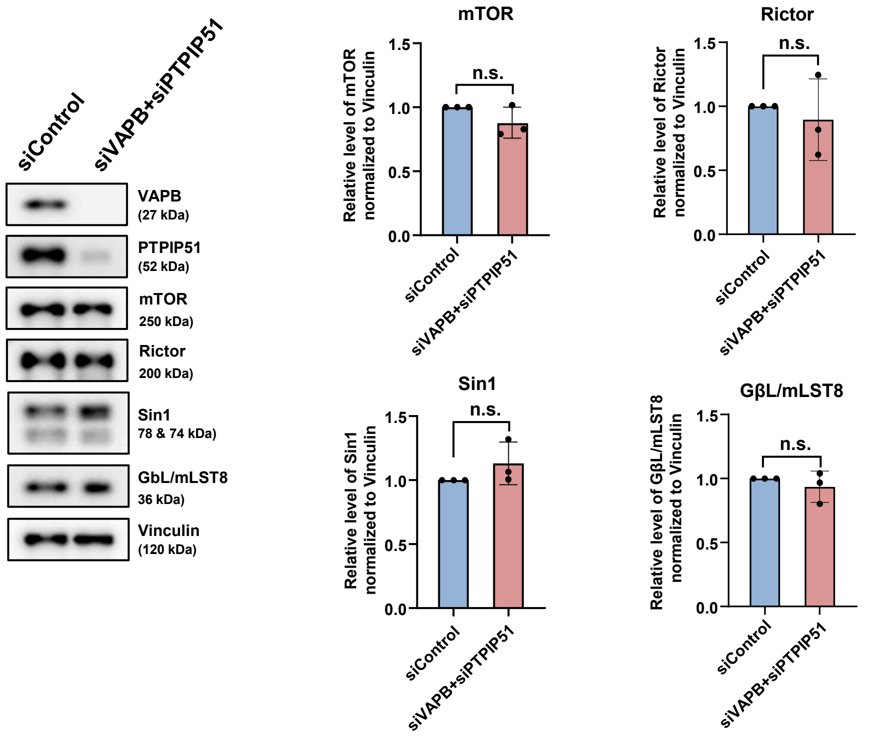

Appendix Figure S7. Depletion of VAPB and PTPIP51 did not affect the levels of mTORC2 complex components

HeLa cells were treated with control (siControl) or VAPB and PTPIP51 (siVAPB+siPTPIP51). *Left panel*: Cells were lysed and analyzed for the levels of indicated mTORC2 complex proteins by western blotting. Vinculin was used as loading control. Respective molecular weights of proteins are as shown in bracket. *Right panels*: Levels of indicated proteins were quantitated, in relation to the loading control Vinculin, derived from 3 experiments. Data are mean ± SEM, unpaired Student's *t* test, n.s., not significant (P>0.05).
