# Supplementary material for: Nup358 restricts ER-mitochondria connectivity by modulating mTORC2/Akt/GSK3β signalling
Source: EMBO Rep. 2024 Jul 18;25(10):14. doi: 10.1038/s44319-024-00204-8 (PMC11466962; doi:10.1038/s44319-024-00204-8)
Supplement: Supplementary file 3 — Source data Fig. 1 [file 44319_2024_204_MOESM3_ESM.zip › Figure 1/1D/Figure 1D - IF images (Left).pdf]

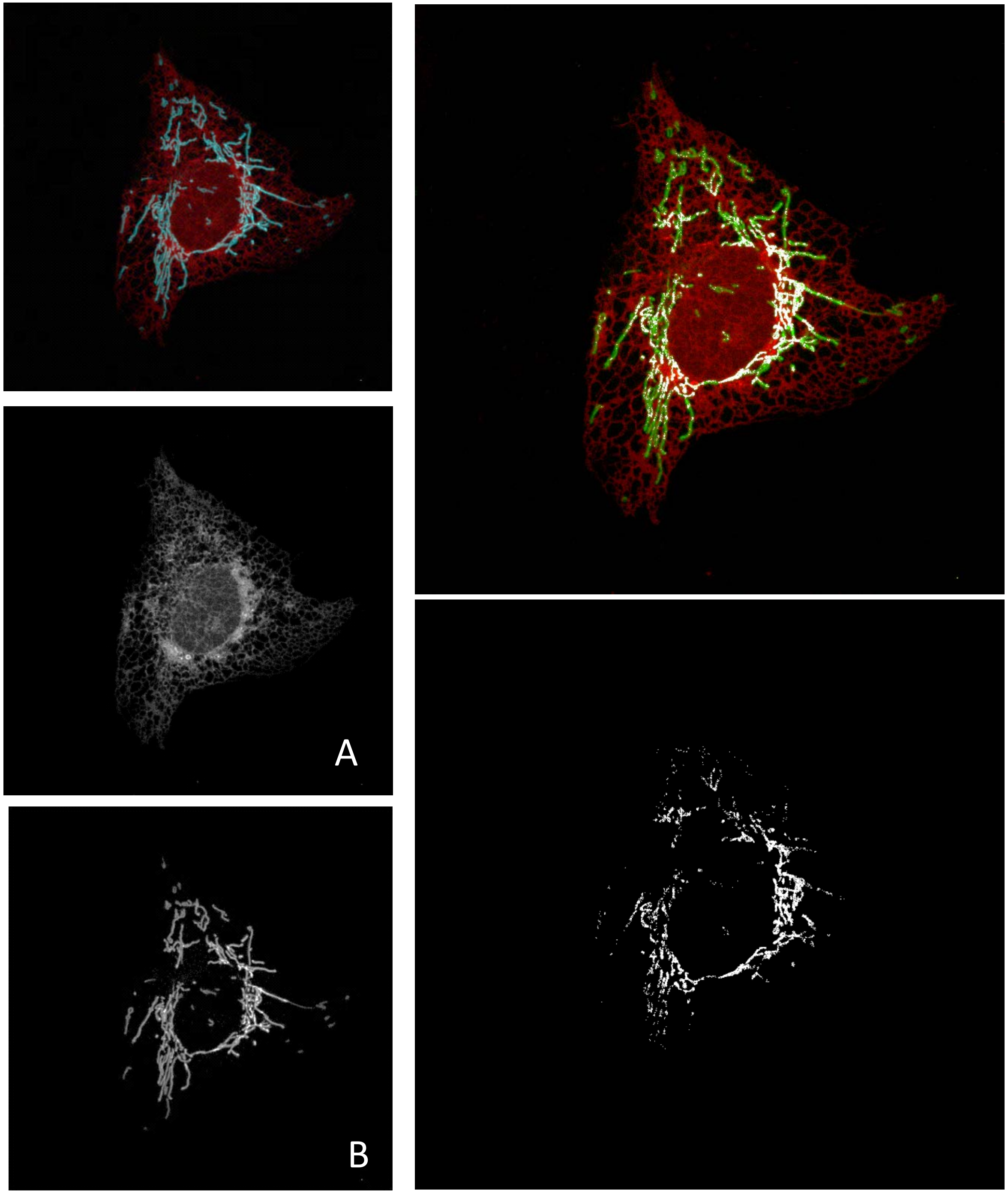

siControl

Image A: csi.tif (red)
Image B: csi.tif (blue)

Manders' Coefficients (using threshold value of 43 for image A and 43 for image B):

M2=0.461 (fraction of B overlapping A)

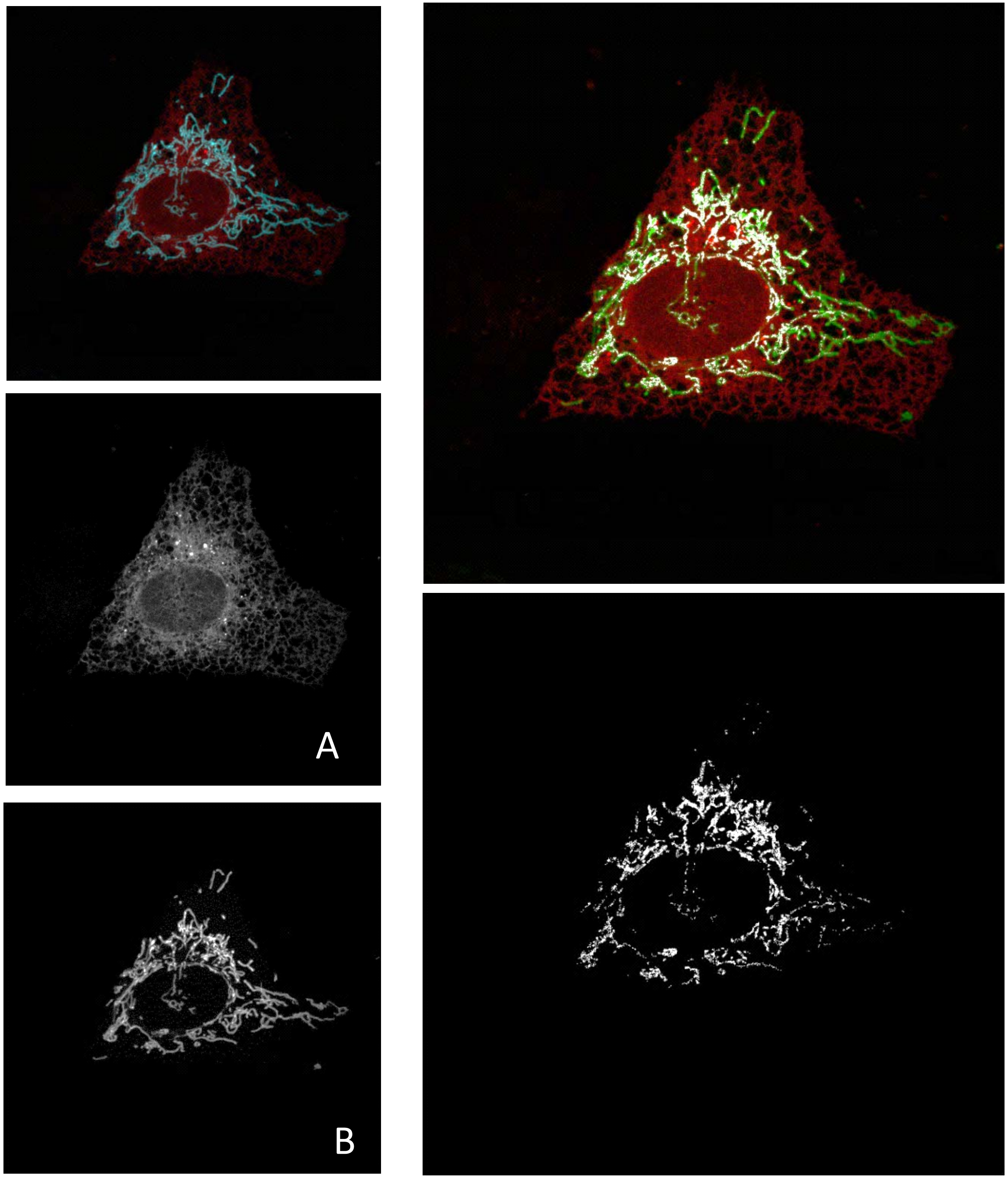

siNup358

Image A: nsi.tif (red)
Image B: nsi.tif (blue)

Manders' Coefficients (using threshold value of 41 for image A and 48 for image B):

M2=0.559 (fraction of B overlapping A)
