# Supplementary figures and images for: Nup358 restricts ER-mitochondria connectivity by modulating mTORC2/Akt/GSK3β signalling
Source: EMBO Rep. 2024 Jul 18;25(10):14. doi: 10.1038/s44319-024-00204-8 (PMC11466962; doi:10.1038/s44319-024-00204-8)

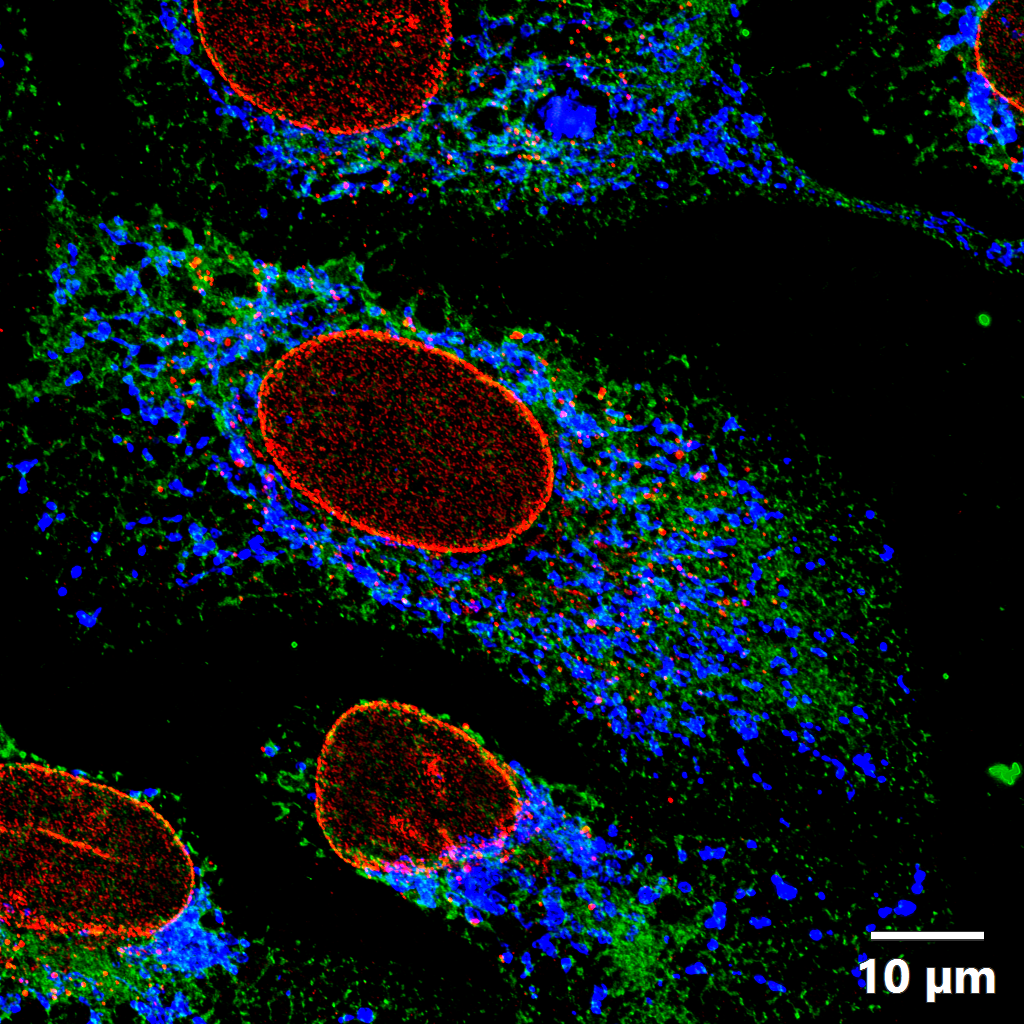

Supplement: Supplementary file 3 — Source data Fig. 1 [file 44319_2024_204_MOESM3_ESM.zip › Figure 1/1A/Figure 1A - Maxmum Intensity Projection.tif]

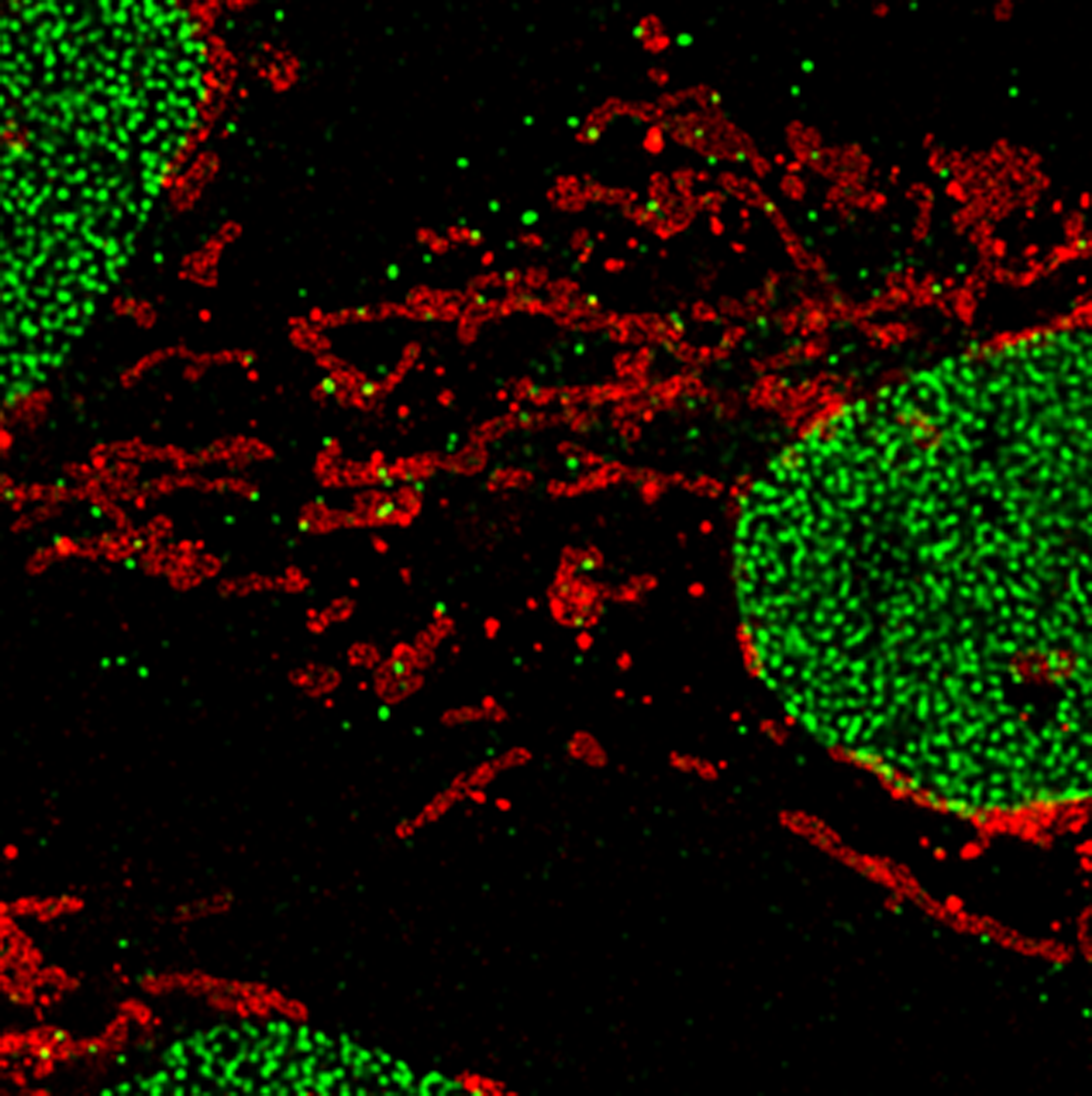

Supplement: Supplementary file 3 — Source data Fig. 1 [file 44319_2024_204_MOESM3_ESM.zip › Figure 1/1B/Figure 1B - STED-deconvoluted image.tif]

# Uncropped western blots related to Figure 1C

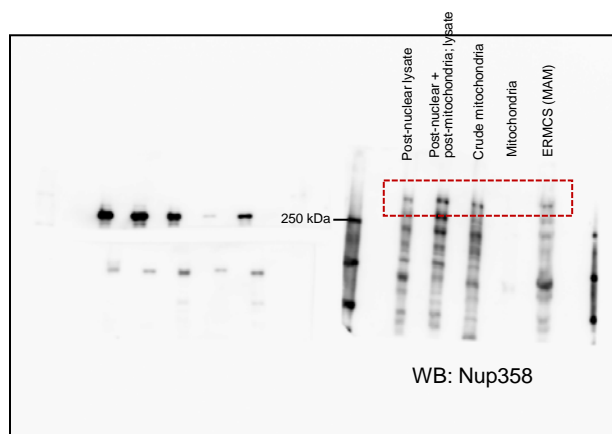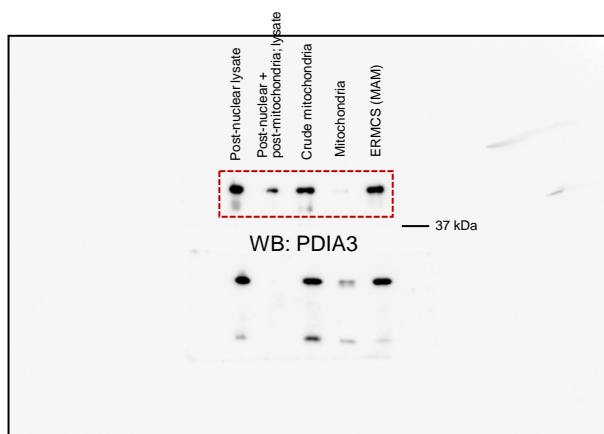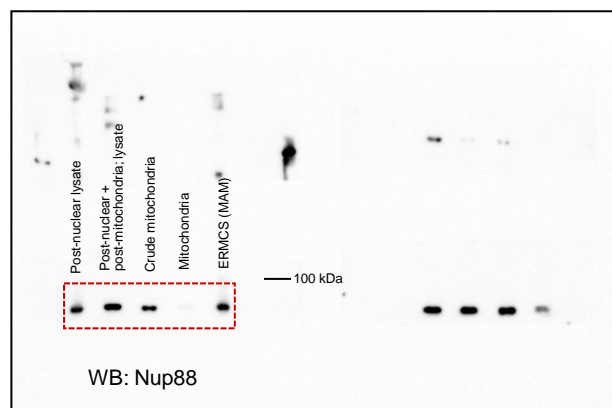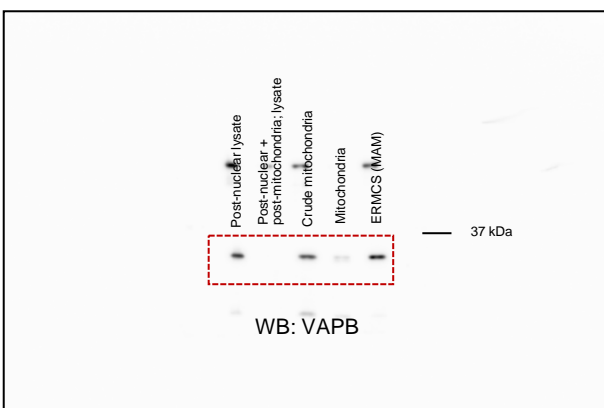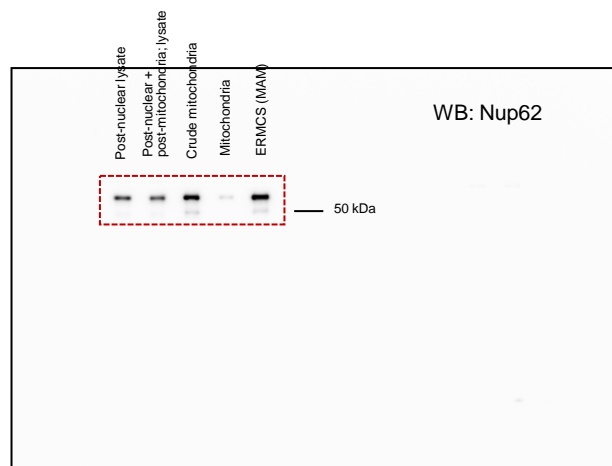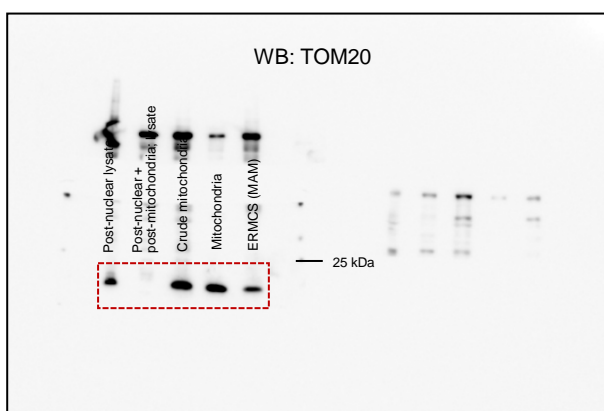

Supplement: Supplementary file 3 — Source data Fig. 1 [file 44319_2024_204_MOESM3_ESM.zip › Figure 1/1C/Figure 1C - Western Blots.pdf]

## Uncropped western blots related to Figure 1D (Right Top)

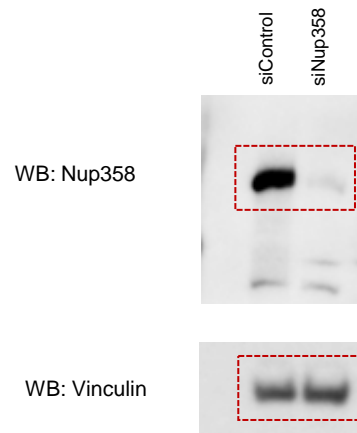

Supplement: Supplementary file 3 — Source data Fig. 1 [file 44319_2024_204_MOESM3_ESM.zip › Figure 1/1D/Figure 1D - Western Blots (Right Top).pdf]

# Related to Figure 1E (Top and Bottom Left)

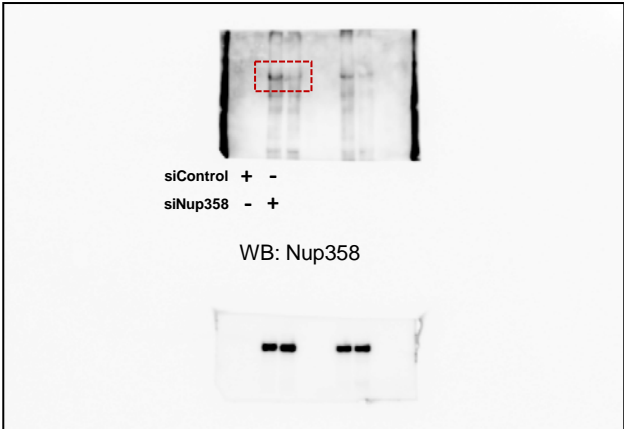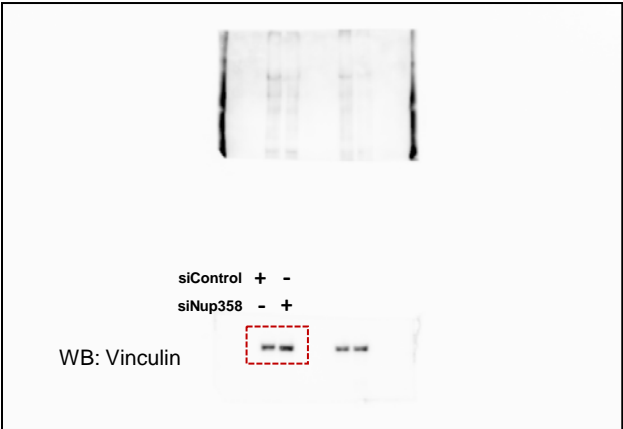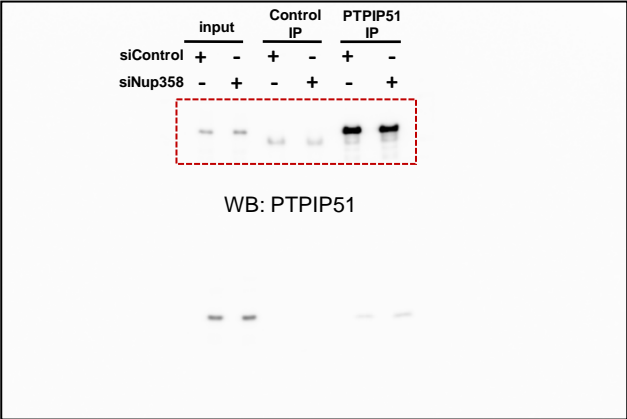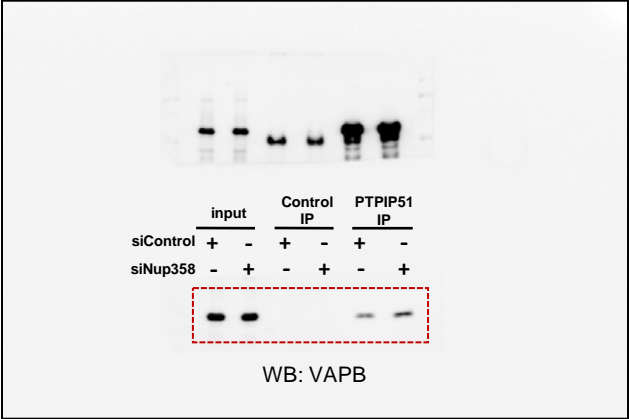

Supplement: Supplementary file 3 — Source data Fig. 1 [file 44319_2024_204_MOESM3_ESM.zip › Figure 1/1E/Figure 1E - Western Blots (Top and Bottom Left).pdf]

## Uncropped western blots related to Figure 1F (Right Top)

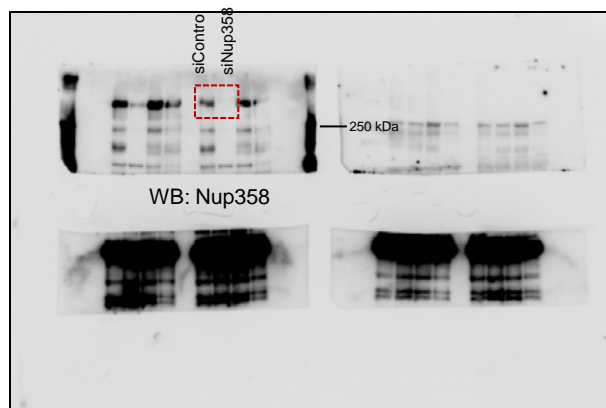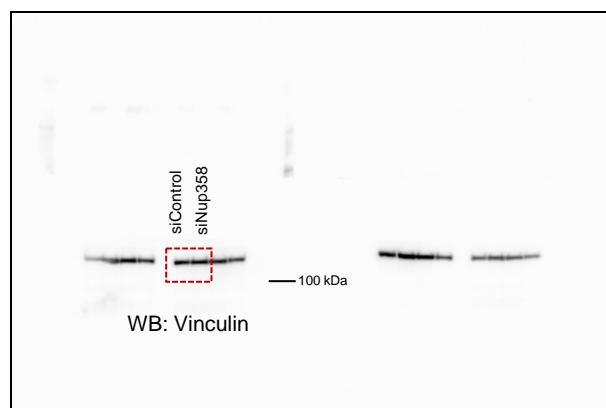

Supplement: Supplementary file 3 — Source data Fig. 1 [file 44319_2024_204_MOESM3_ESM.zip › Figure 1/1F/Figure 1F - Western Blots (Right Top).pdf]

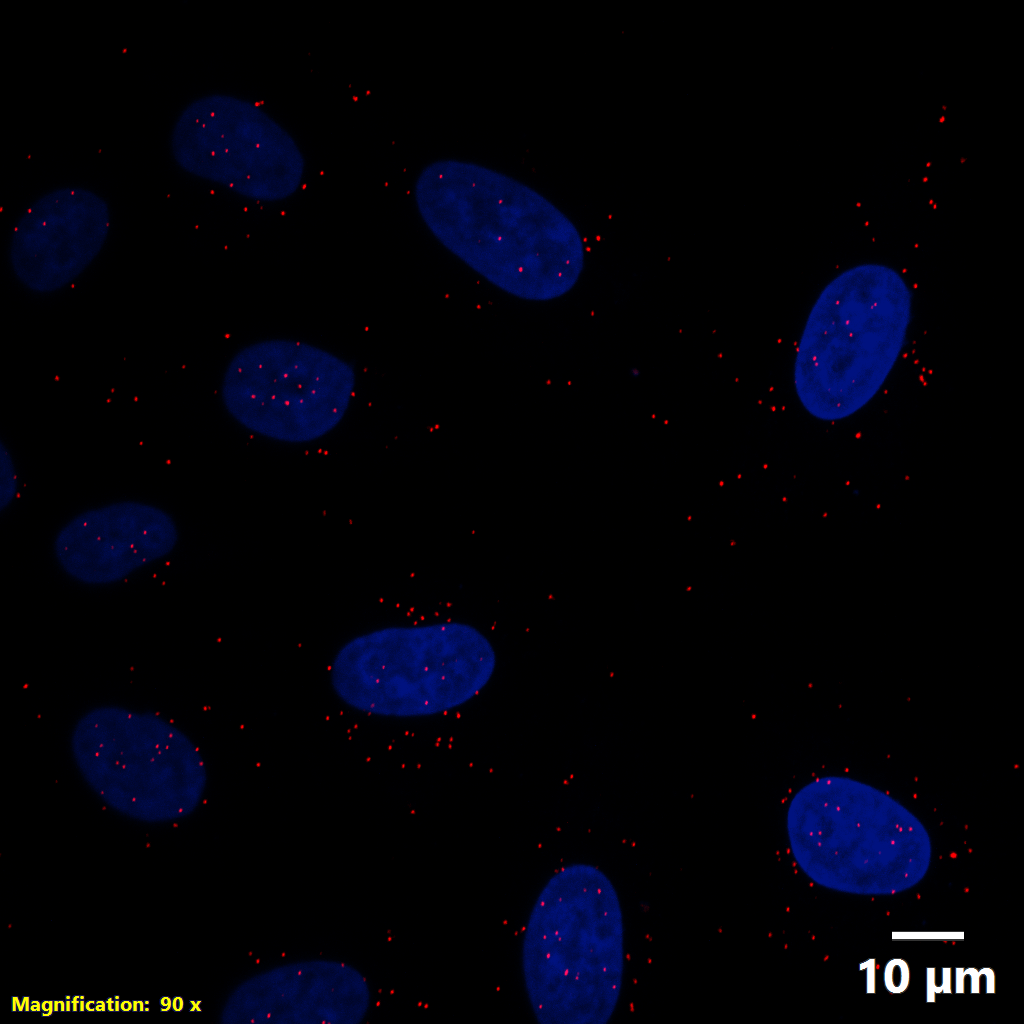

Supplement: Supplementary file 3 — Source data Fig. 1 [file 44319_2024_204_MOESM3_ESM.zip › Figure 1/1F/siControl-VAPB+PTPIP51 PLA-IF image.tif]

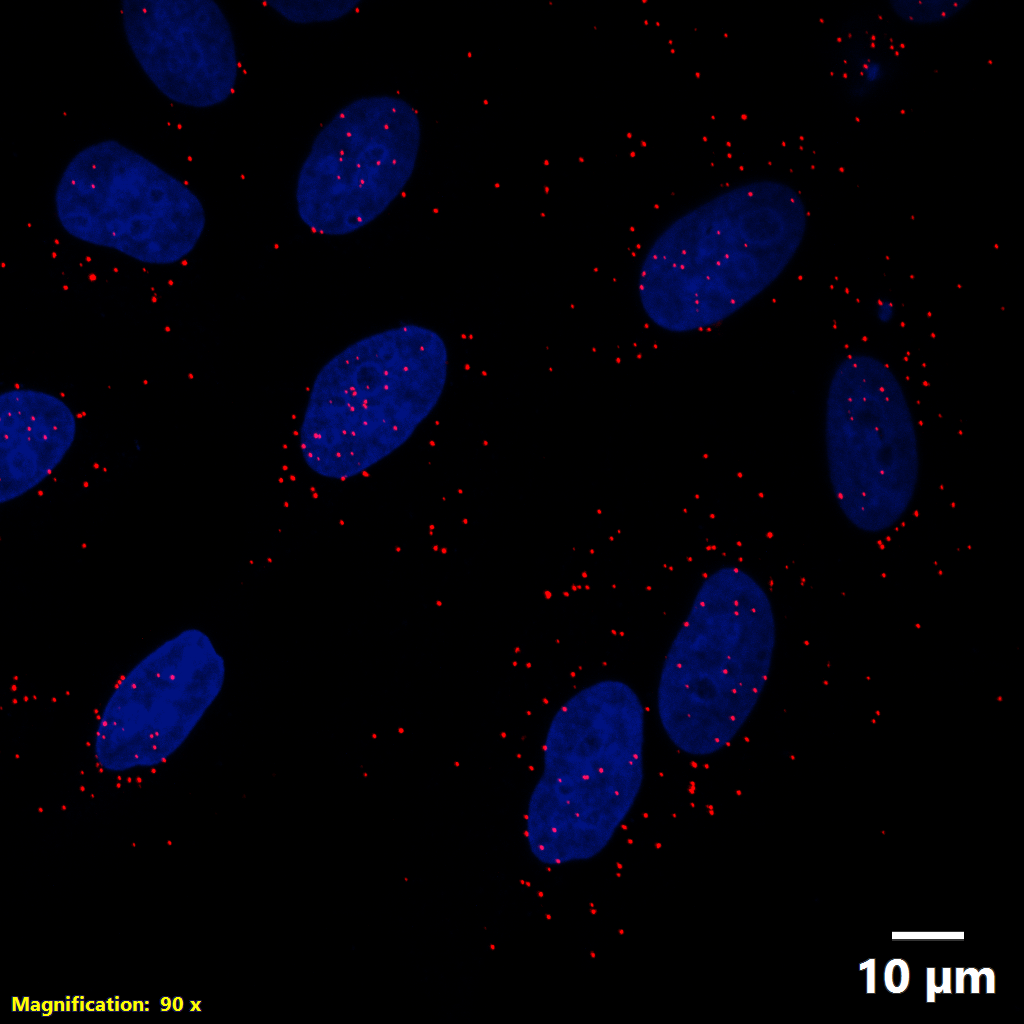

Supplement: Supplementary file 3 — Source data Fig. 1 [file 44319_2024_204_MOESM3_ESM.zip › Figure 1/1F/siNup358-VAPB+PTPIP51 PLA-IF image.tif]

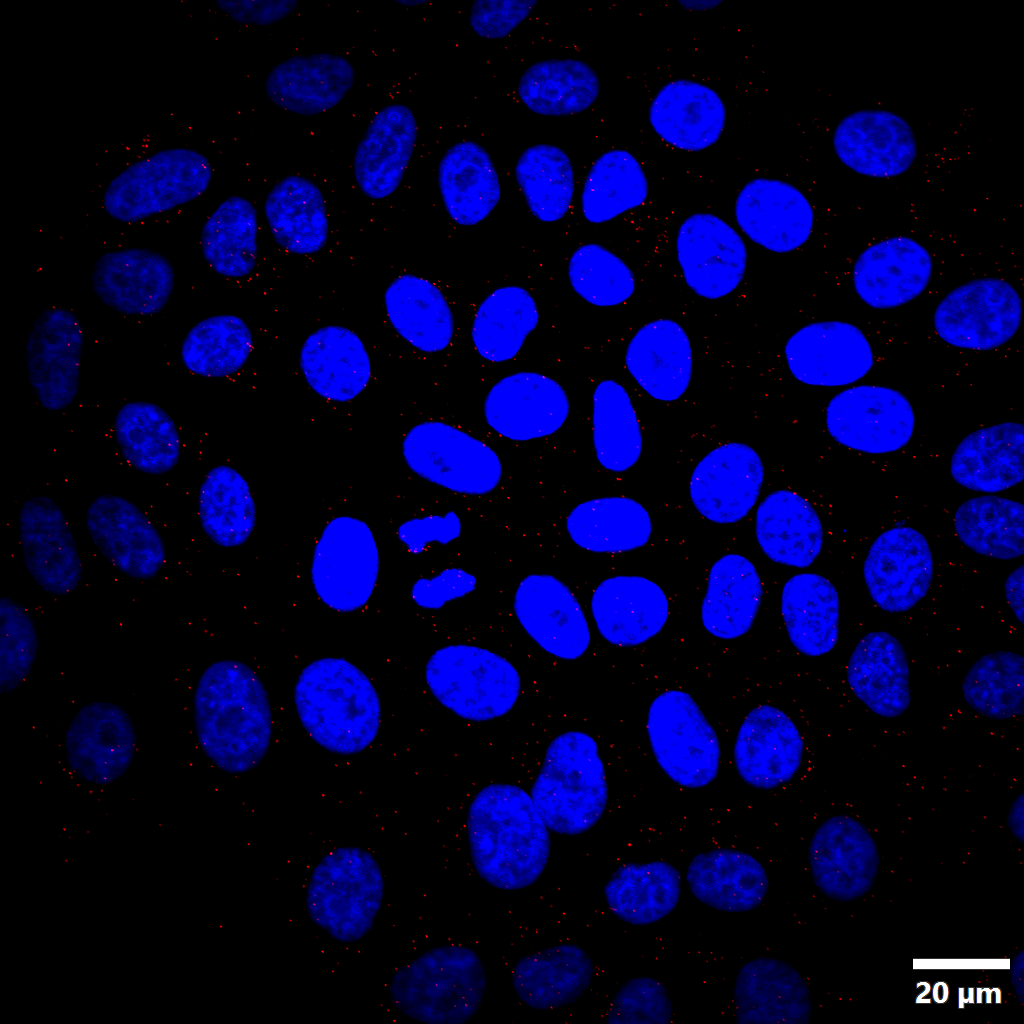

Supplement: Supplementary file 3 — Source data Fig. 1 [file 44319_2024_204_MOESM3_ESM.zip › Figure 1/1G/siControl-BAP31-Fis1 PLA Image.tif]

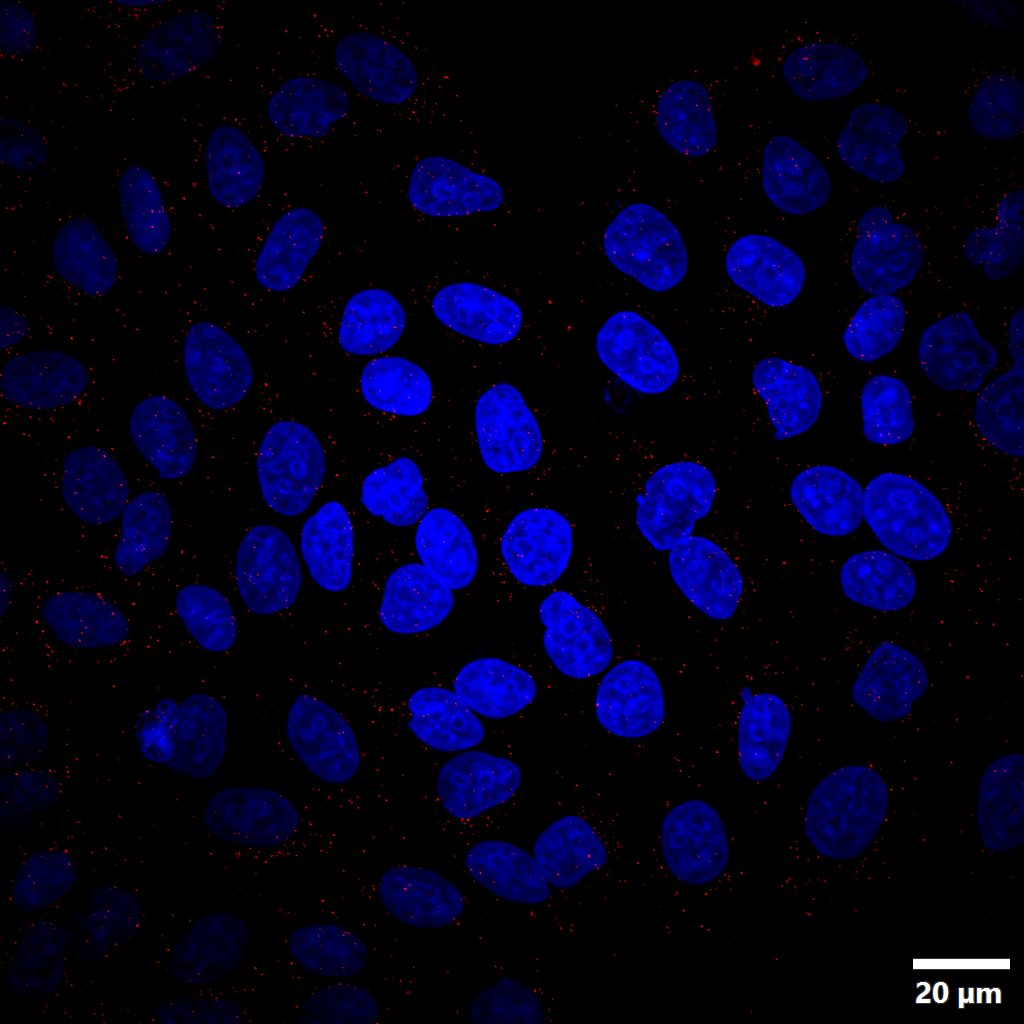

Supplement: Supplementary file 3 — Source data Fig. 1 [file 44319_2024_204_MOESM3_ESM.zip › Figure 1/1G/siNup358-BAP31-Fis1 PLA Image.tif]

## Uncropped western blots related to Figure 2A (top)

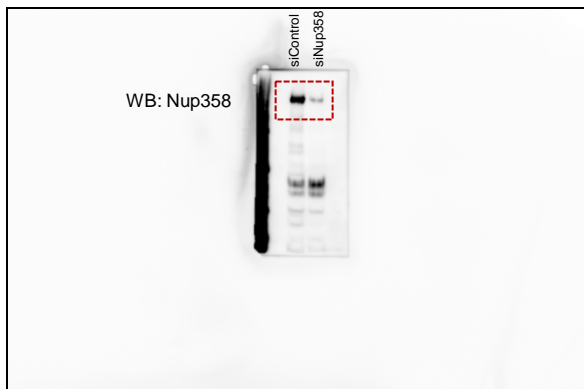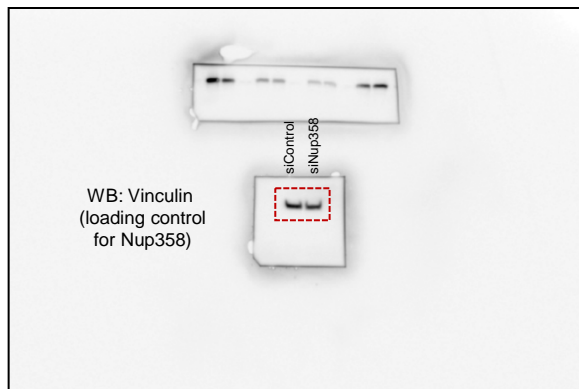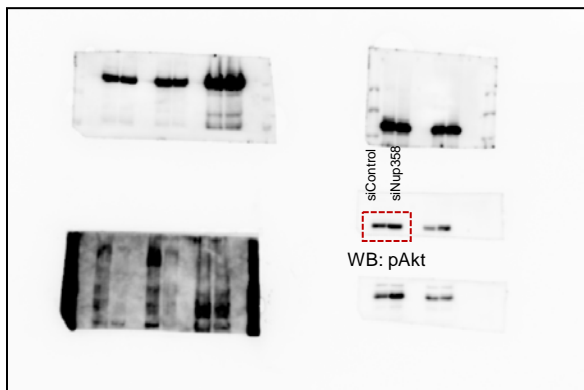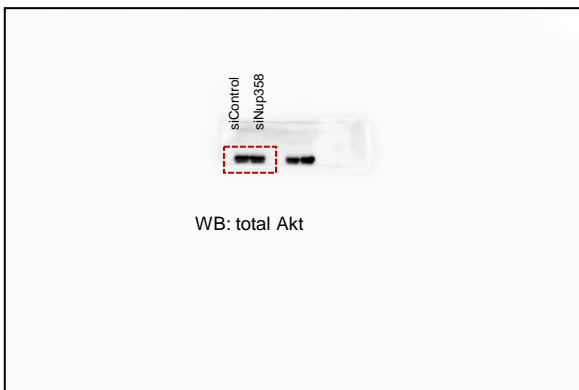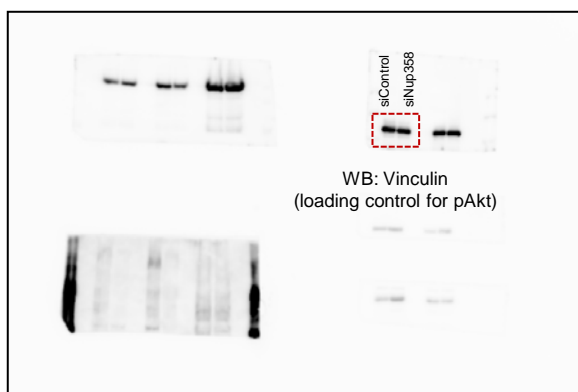

Supplement: Supplementary file 4 — Source data Fig. 2 [file 44319_2024_204_MOESM4_ESM.zip › Figure 2/2A/Figure 2A Western Blots (Top).pdf]

## Uncropped western blots related to Figure 2B (top)

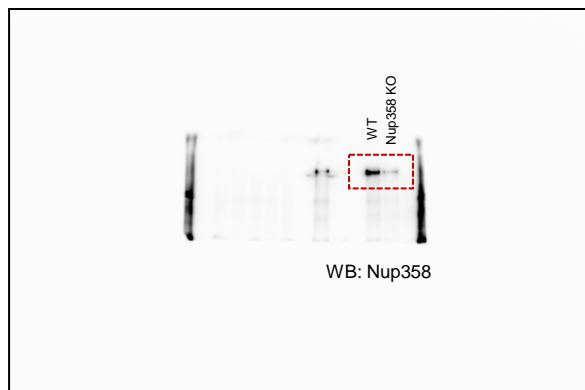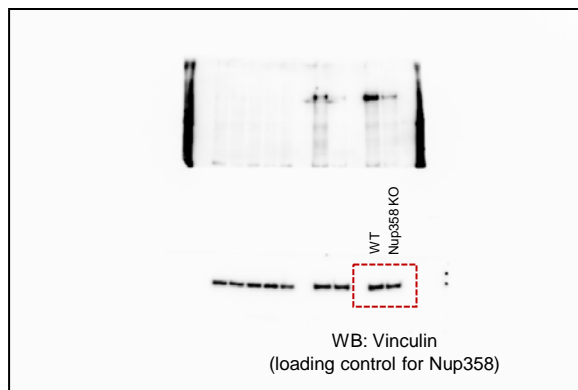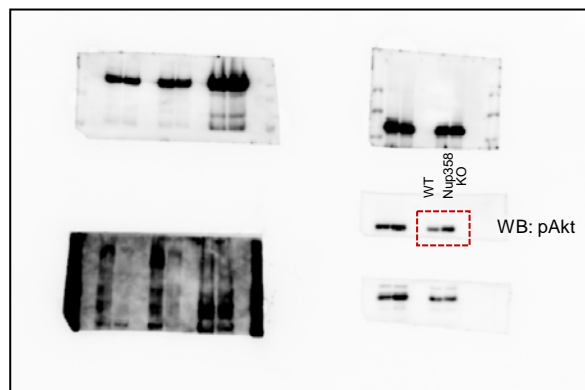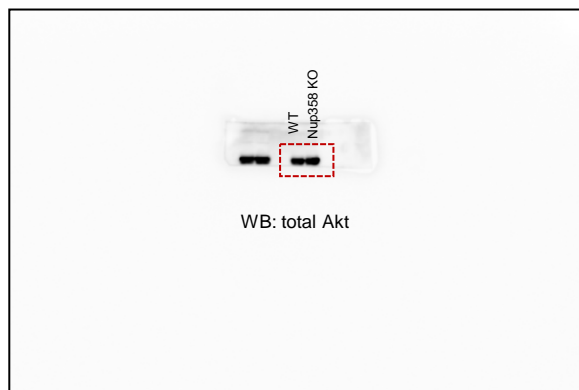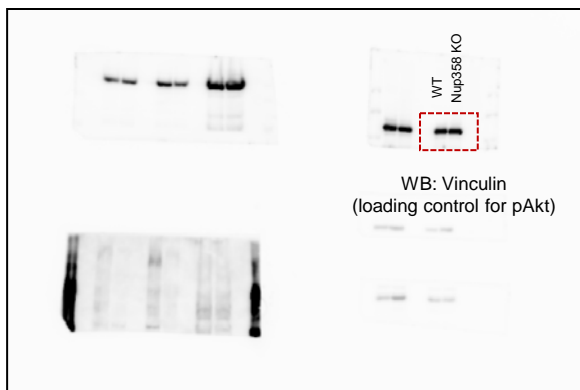

Supplement: Supplementary file 4 — Source data Fig. 2 [file 44319_2024_204_MOESM4_ESM.zip › Figure 2/2B/Figure 2B Western Blots (Top).pdf]

Uncropped western blots related to Figure 2A (Left Bottom)

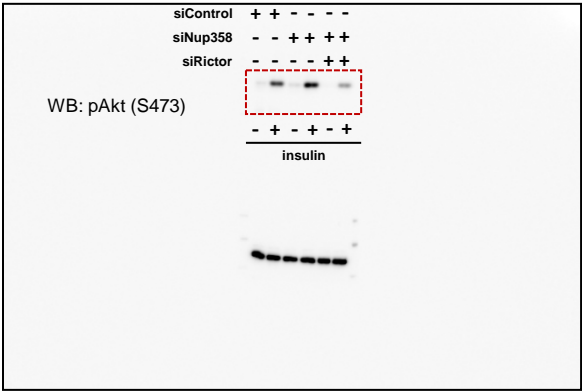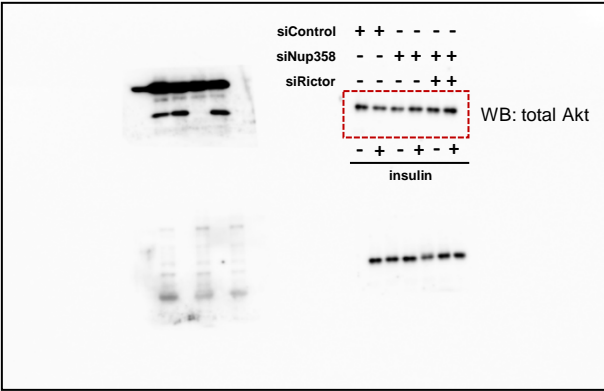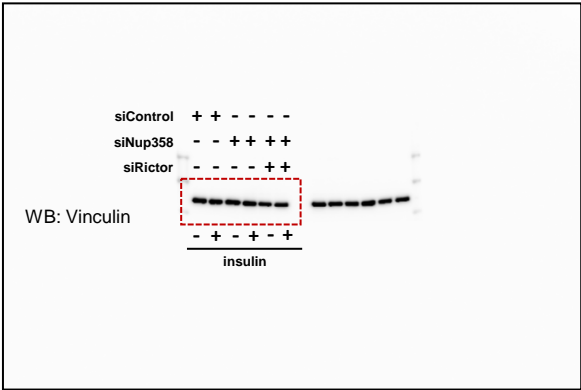

Supplement: Supplementary file 4 — Source data Fig. 2 [file 44319_2024_204_MOESM4_ESM.zip › Figure 2/2C/Figure 2C Western Blots (Left Bottom).pdf]

Uncropped western blots related to Figure 2C (top)

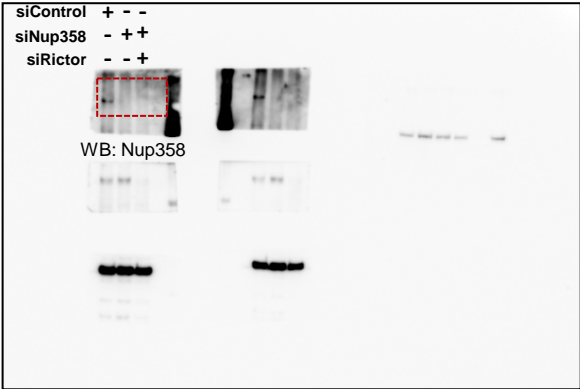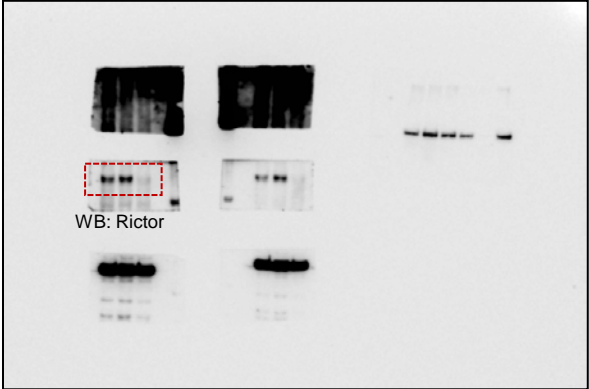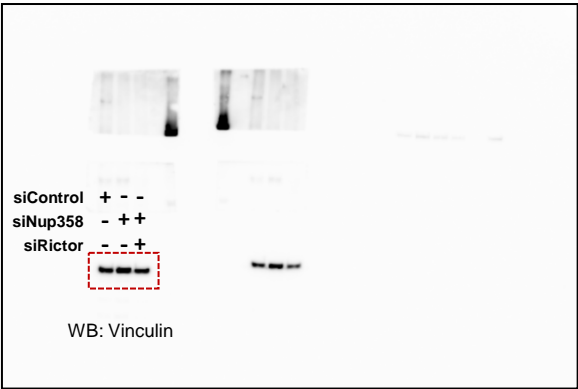

Supplement: Supplementary file 4 — Source data Fig. 2 [file 44319_2024_204_MOESM4_ESM.zip › Figure 2/2C/Figure 2C Western Blots (Top).pdf]

Uncropped western blots related to Figure 2D (Fourth Top Panel)

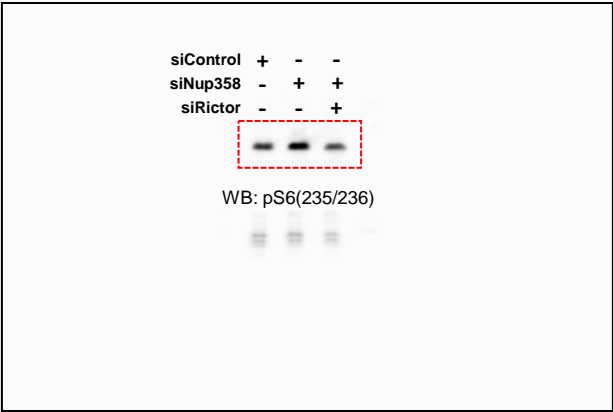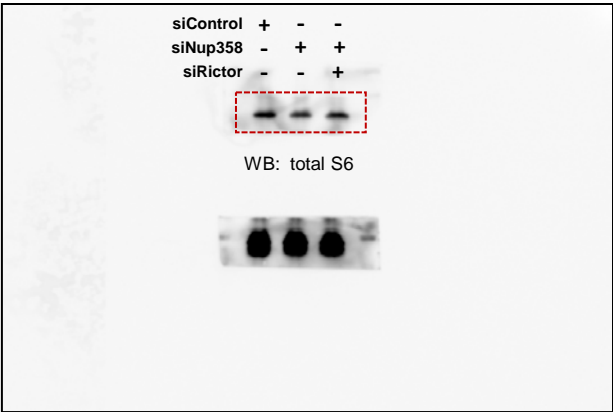

Supplement: Supplementary file 4 — Source data Fig. 2 [file 44319_2024_204_MOESM4_ESM.zip › Figure 2/2D/Figure 2D Western Blots (Fourth top panel).pdf]

Uncropped western blots related to Figure 2D (Left First Panel)

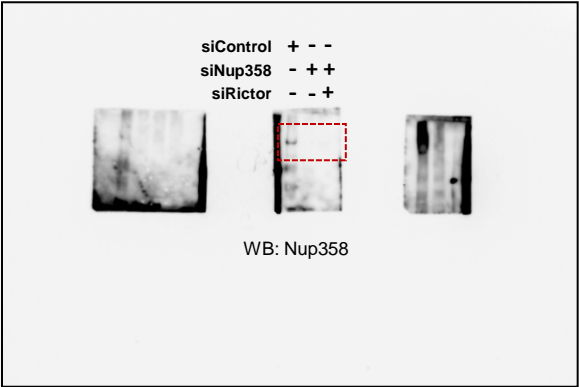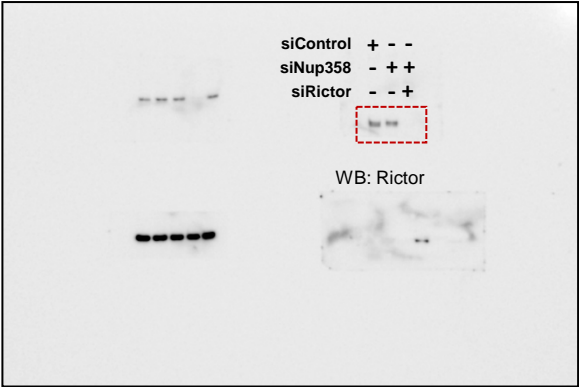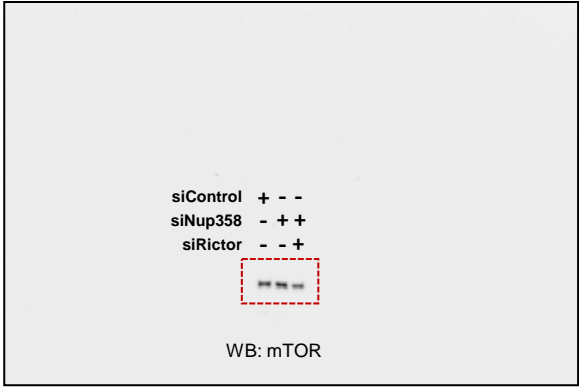

Supplement: Supplementary file 4 — Source data Fig. 2 [file 44319_2024_204_MOESM4_ESM.zip › Figure 2/2D/Figure 2D Western Blots (Left - First Panel).pdf]

Uncropped western blots related to Figure 2D (Second Top Panel)

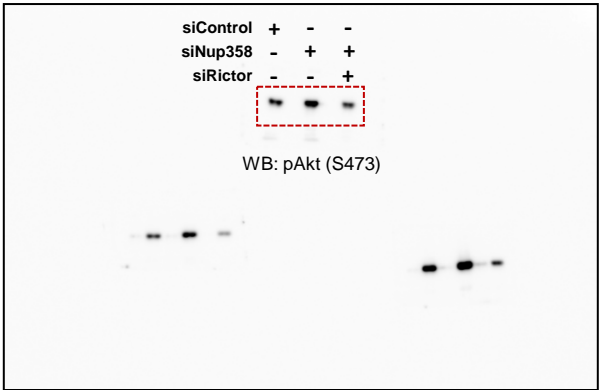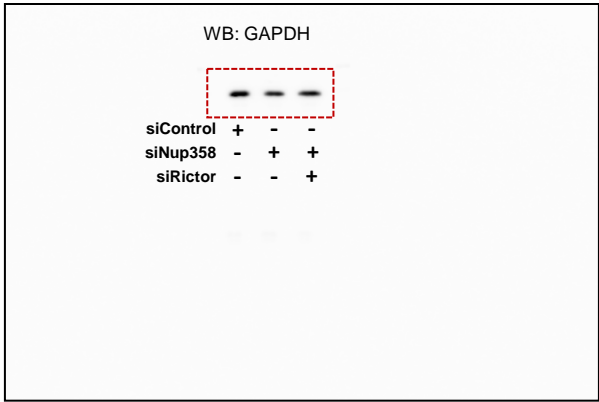

Supplement: Supplementary file 4 — Source data Fig. 2 [file 44319_2024_204_MOESM4_ESM.zip › Figure 2/2D/Figure 2D Western Blots (Second top panel).pdf]

Uncropped western blots related to Figure 2D (Third Top Panel)

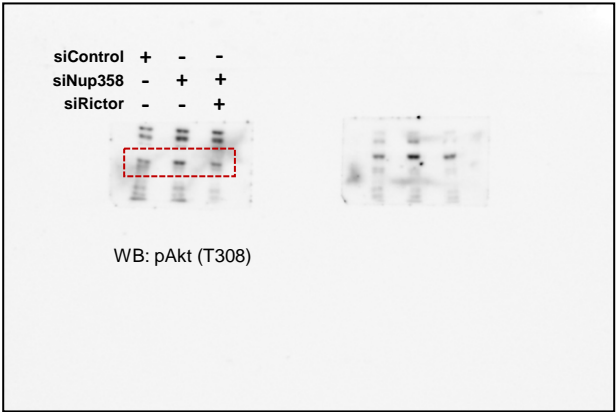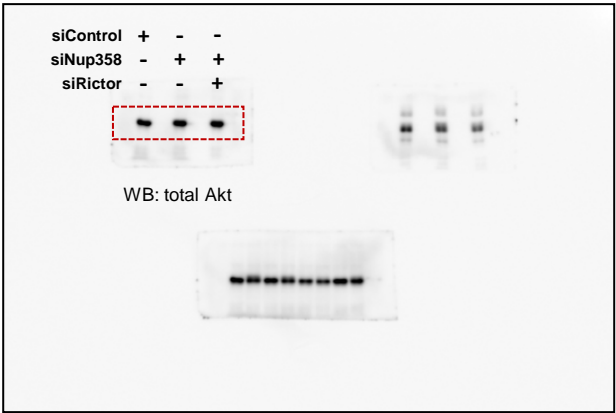

Supplement: Supplementary file 4 — Source data Fig. 2 [file 44319_2024_204_MOESM4_ESM.zip › Figure 2/2D/Figure 2D Western Blots (Third top panel).pdf]

## Uncropped western blots related to Figure 2E (Left)

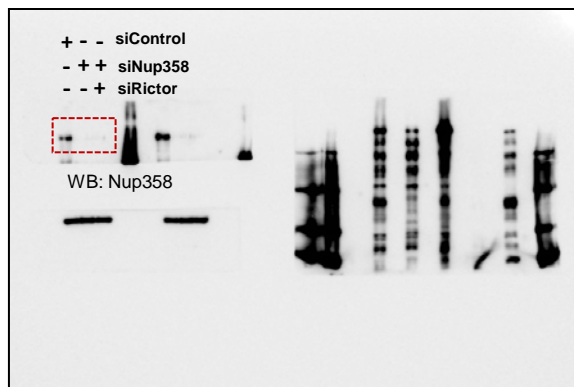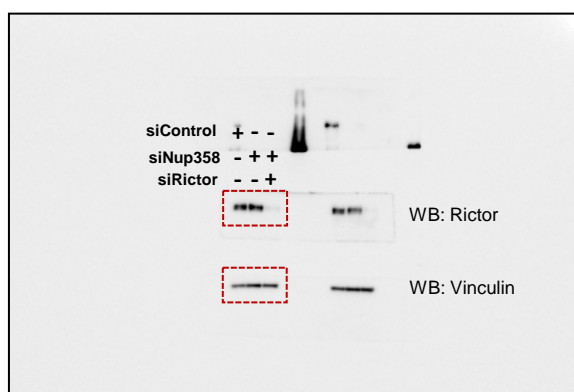

Supplement: Supplementary file 4 — Source data Fig. 2 [file 44319_2024_204_MOESM4_ESM.zip › Figure 2/2E/Figure 2E Western Blots (Right Top).pdf]

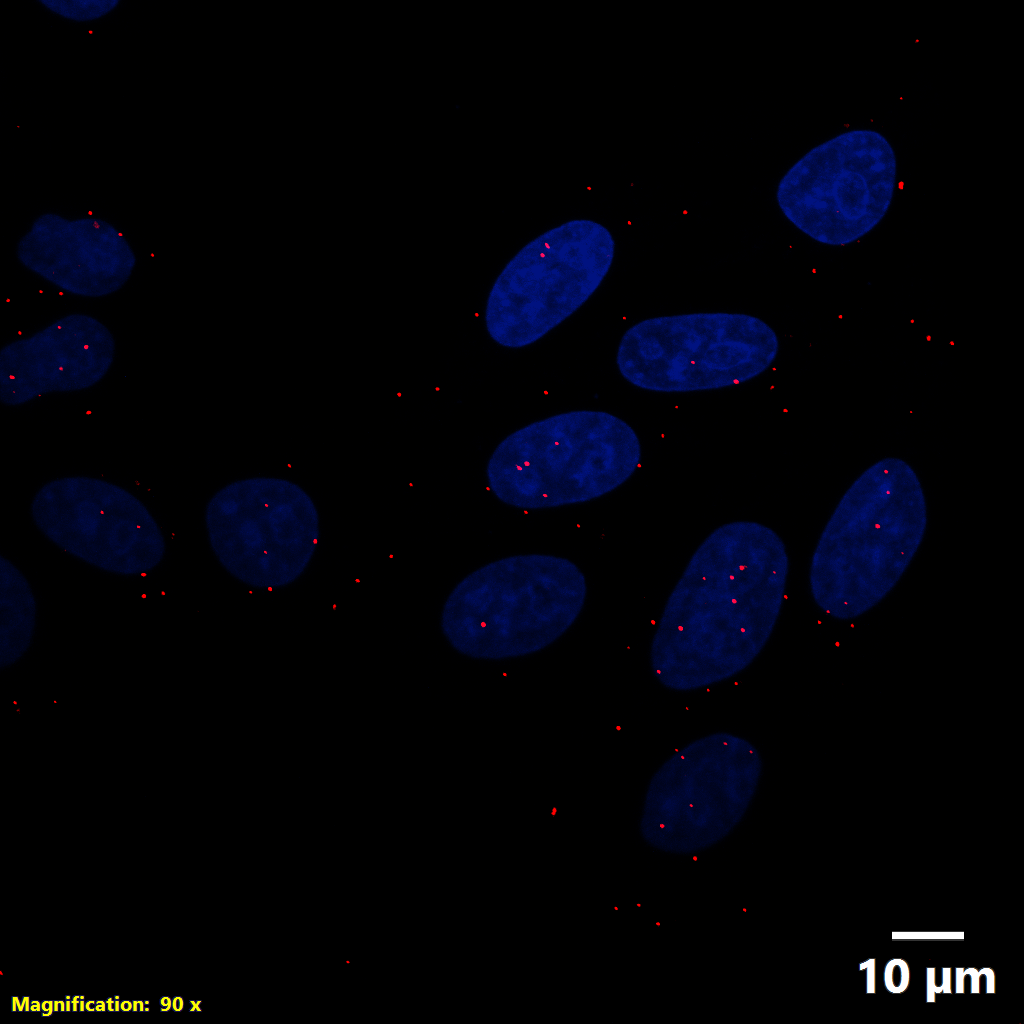

Supplement: Supplementary file 4 — Source data Fig. 2 [file 44319_2024_204_MOESM4_ESM.zip › Figure 2/2E/siControl PLA image.tif]

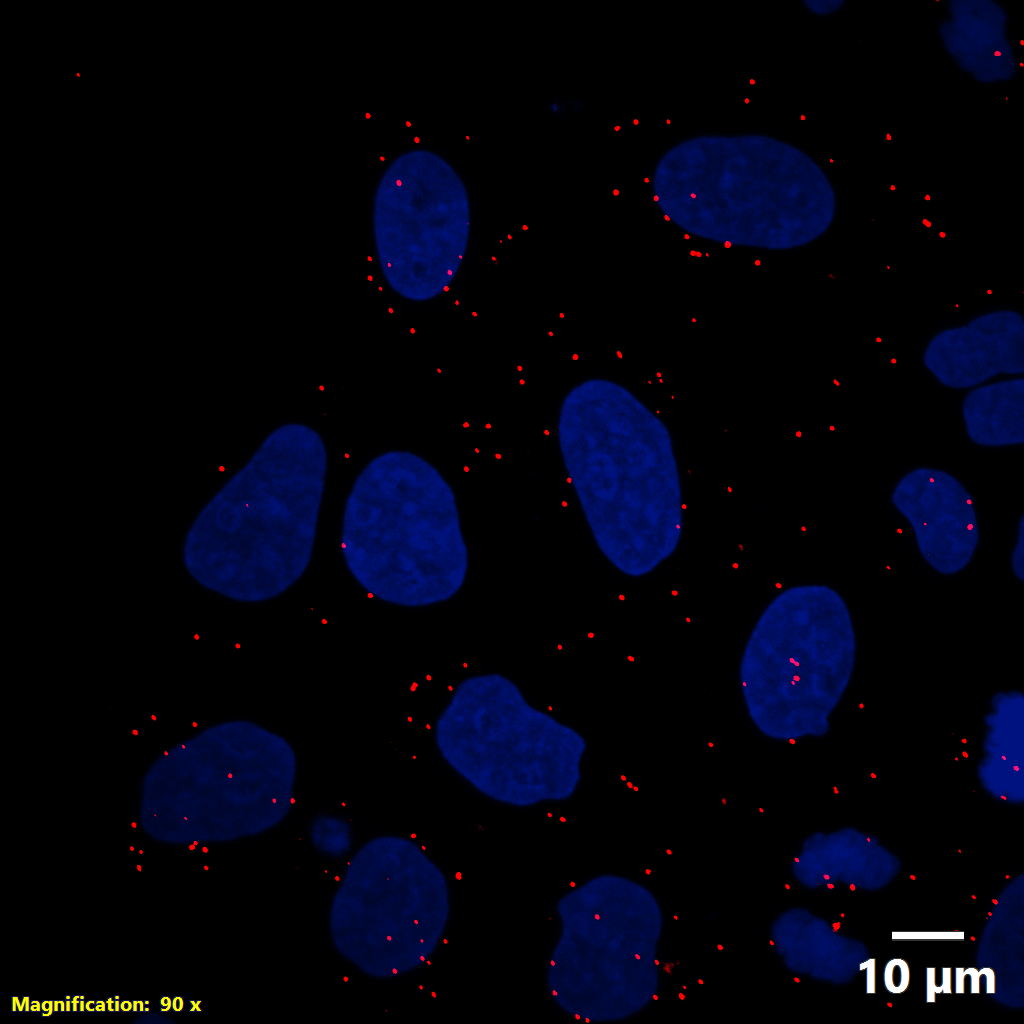

Supplement: Supplementary file 4 — Source data Fig. 2 [file 44319_2024_204_MOESM4_ESM.zip › Figure 2/2E/siNup358 PLA image.tif]

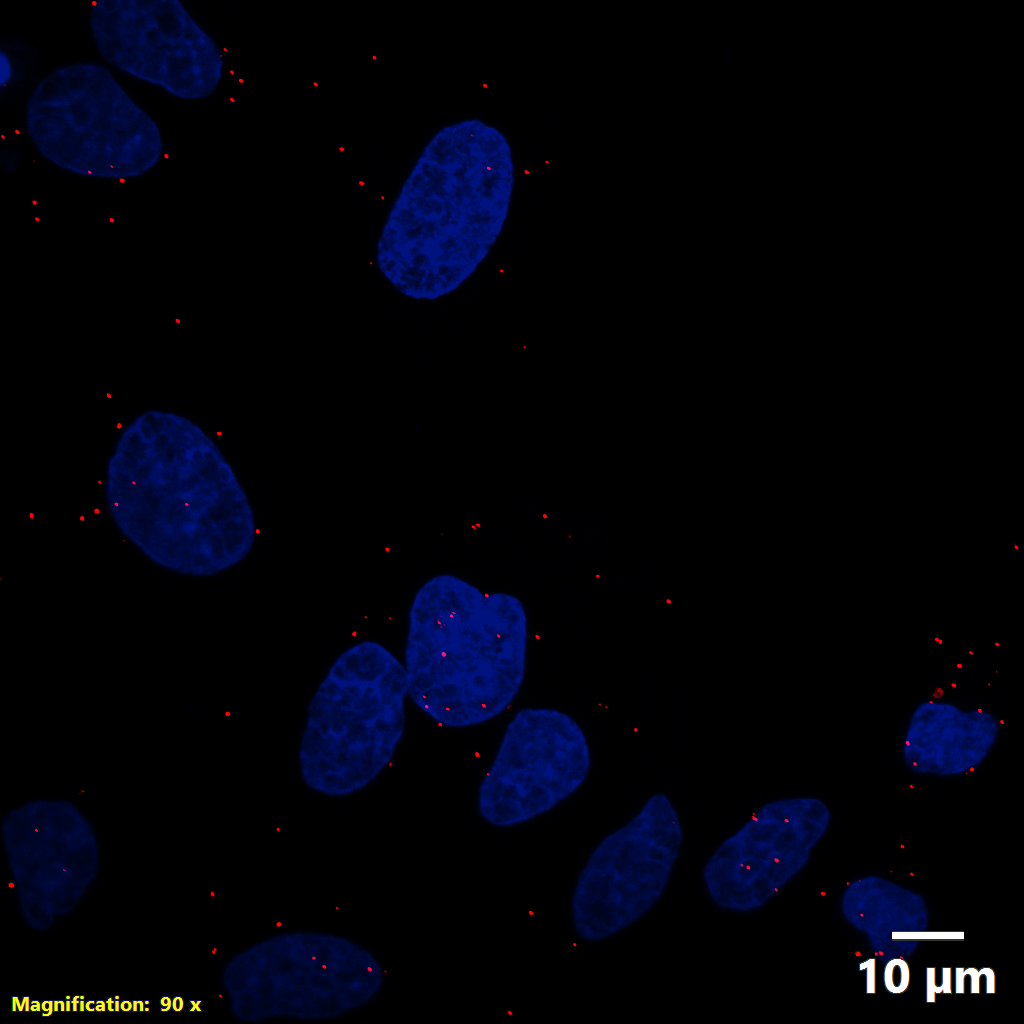

Supplement: Supplementary file 4 — Source data Fig. 2 [file 44319_2024_204_MOESM4_ESM.zip › Figure 2/2E/siNup358+siRictor PLA image.tif]

## Uncropped western blots related to Figure 2F (Left)

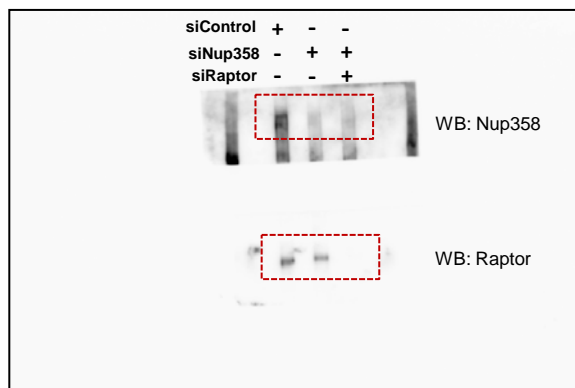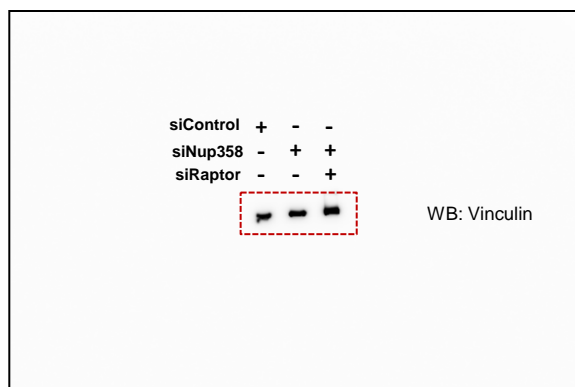

Supplement: Supplementary file 4 — Source data Fig. 2 [file 44319_2024_204_MOESM4_ESM.zip › Figure 2/2F/Figure 2F Western Blots (Right Top).pdf]

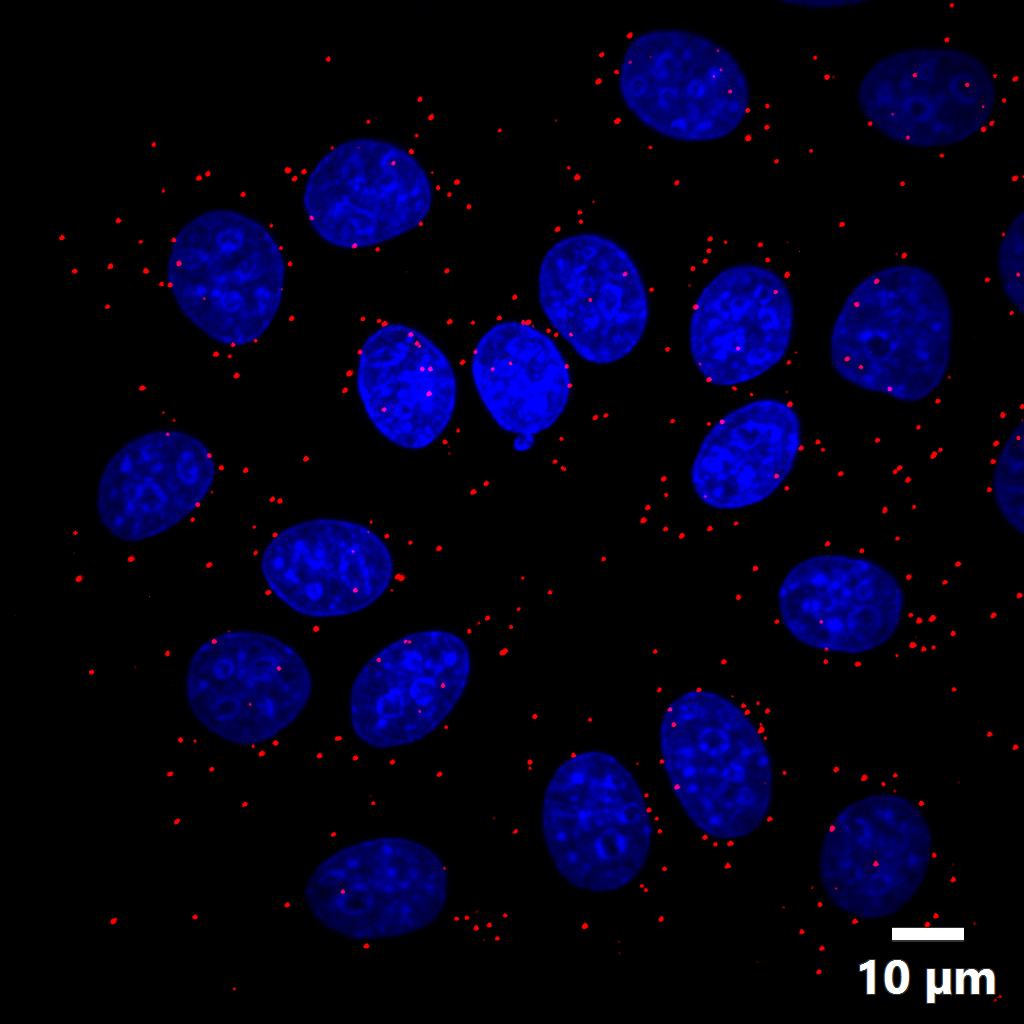

Supplement: Supplementary file 4 — Source data Fig. 2 [file 44319_2024_204_MOESM4_ESM.zip › Figure 2/2F/siControl PLA image.tif]

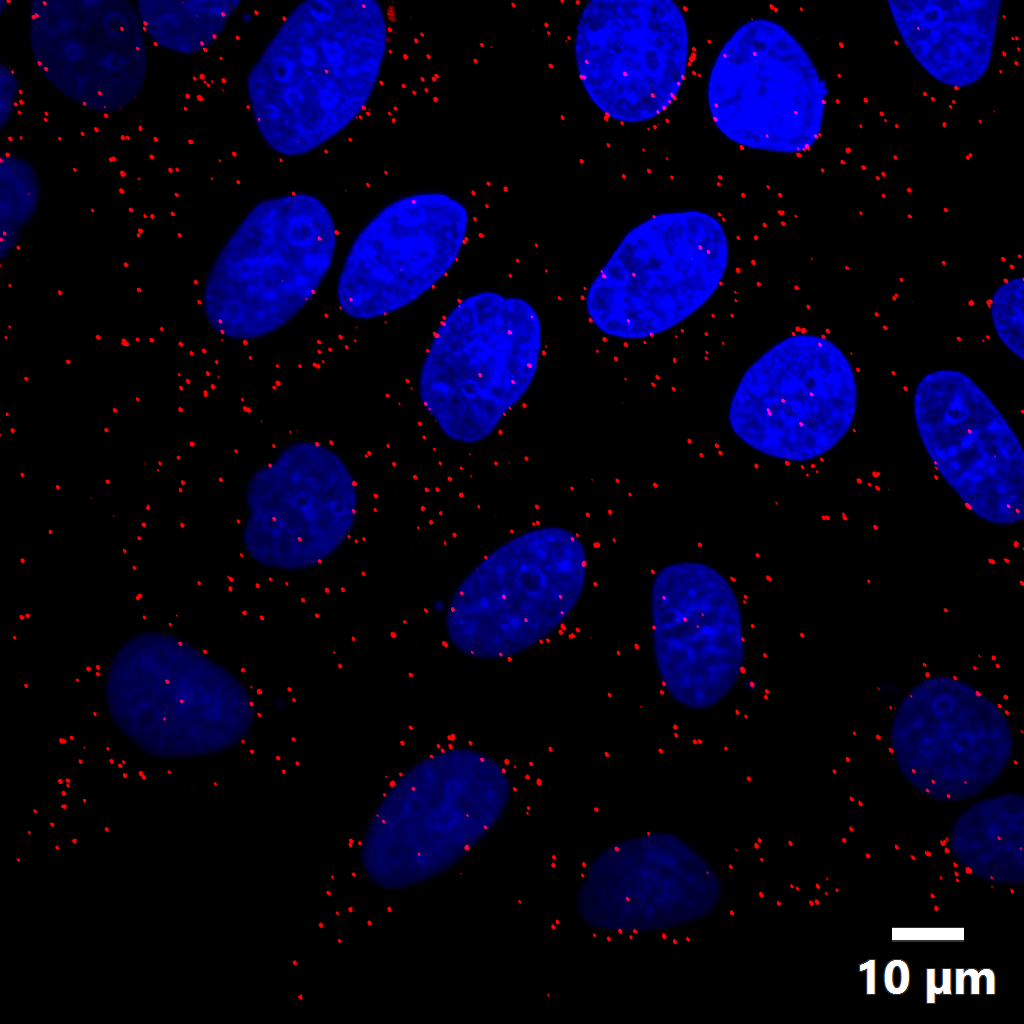

Supplement: Supplementary file 4 — Source data Fig. 2 [file 44319_2024_204_MOESM4_ESM.zip › Figure 2/2F/siNup358 PLA image.tif]

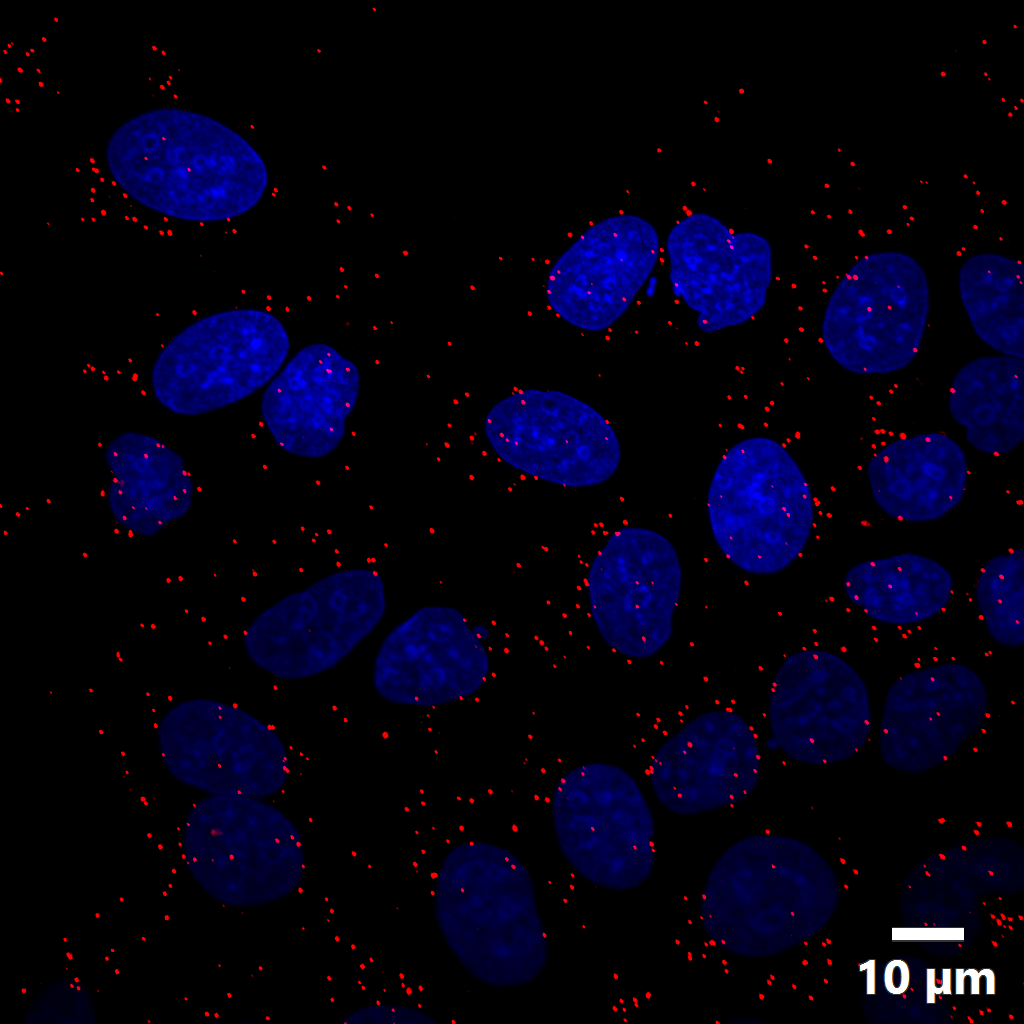

Supplement: Supplementary file 4 — Source data Fig. 2 [file 44319_2024_204_MOESM4_ESM.zip › Figure 2/2F/siNup358+siRaptor PLA image.tif]

## Uncropped western blots related to Figure 1A (Left Bottom)

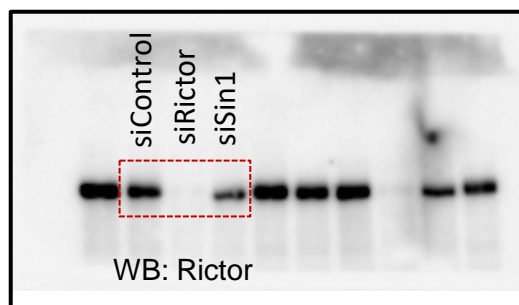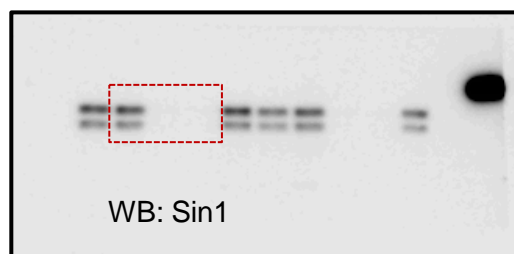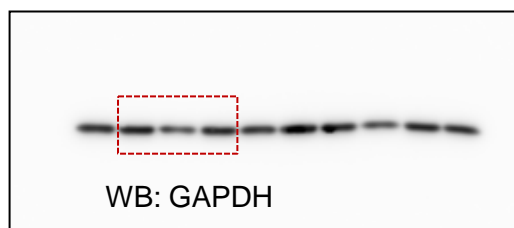

## WB: GAPDH

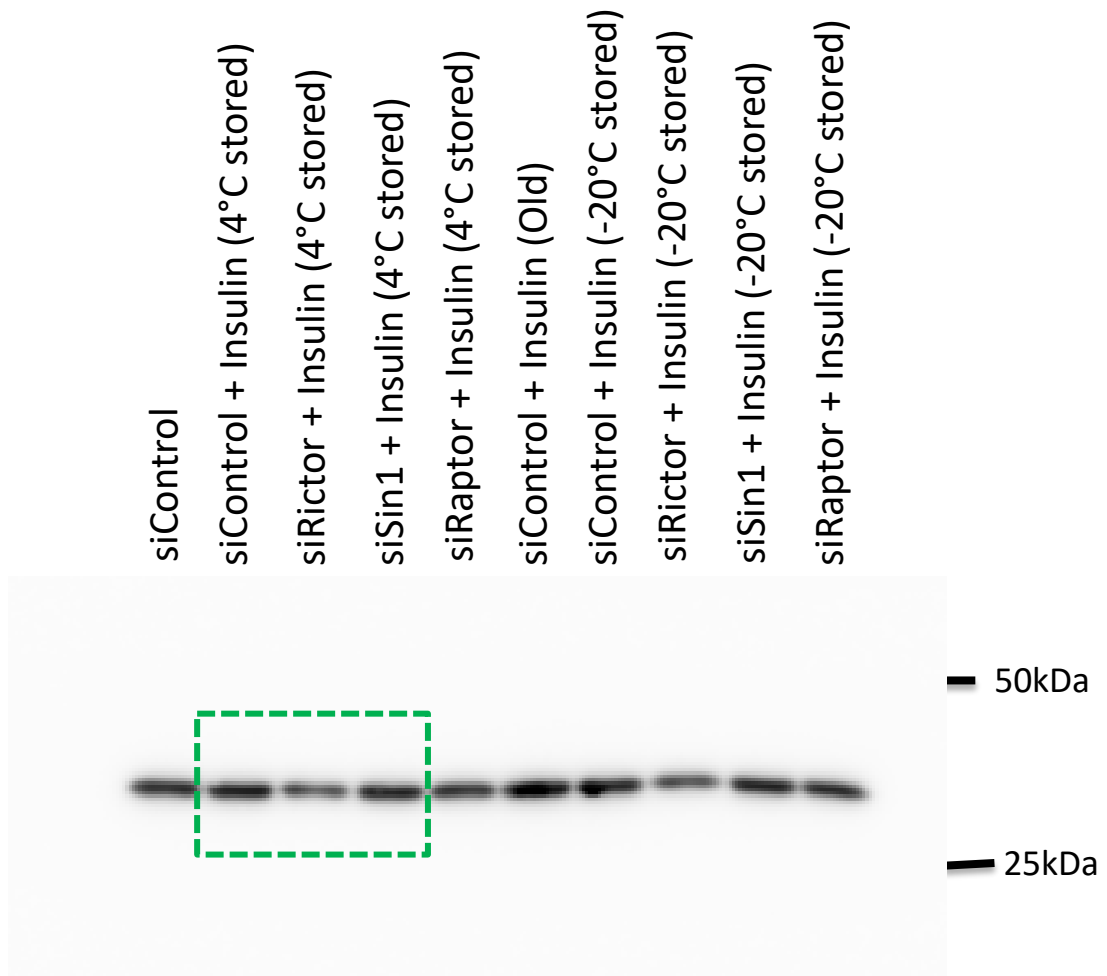

Supplement: Supplementary file 5 — Source data Fig. 3 [file 44319_2024_204_MOESM5_ESM.zip › Figure 3/3A/Figure 3A_Western Blots (Bottom Left).pdf]

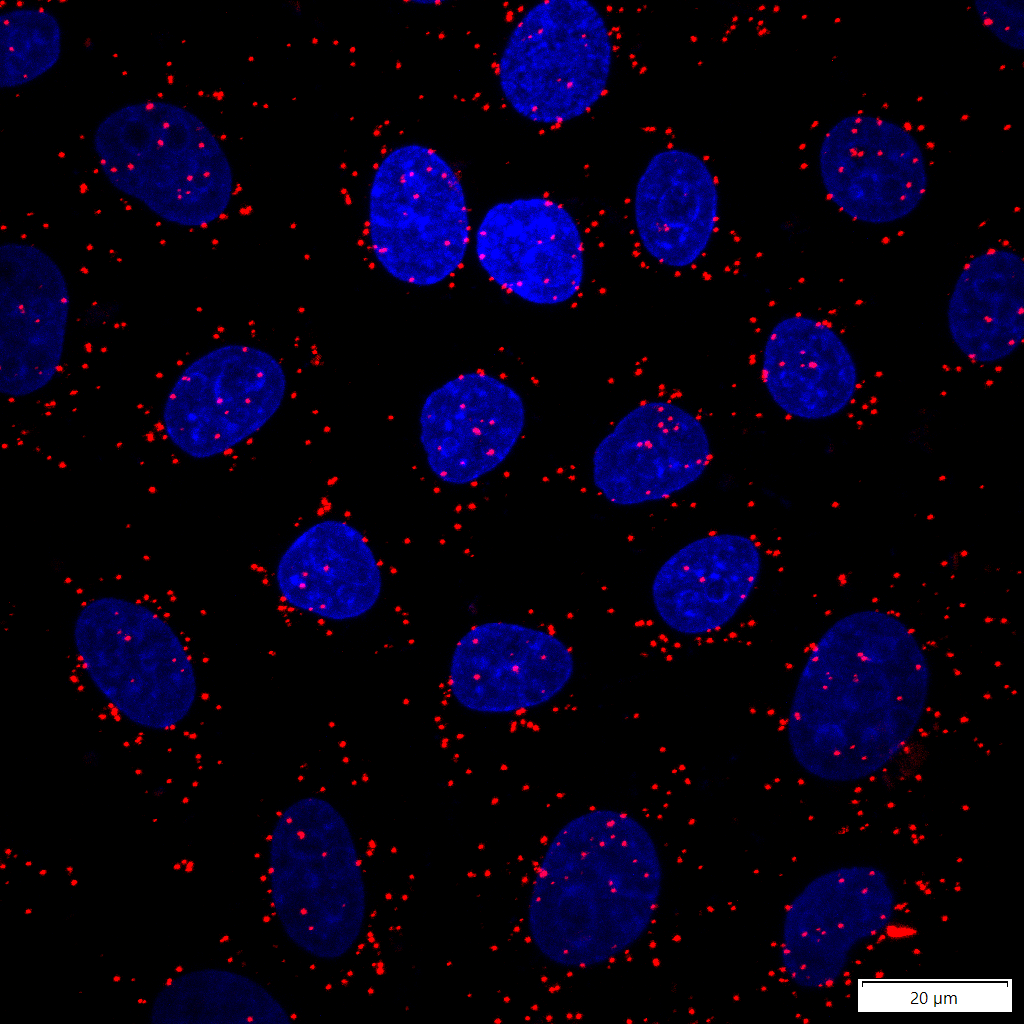

Supplement: Supplementary file 5 — Source data Fig. 3 [file 44319_2024_204_MOESM5_ESM.zip › Figure 3/3A/siControl - Insulin.tif]

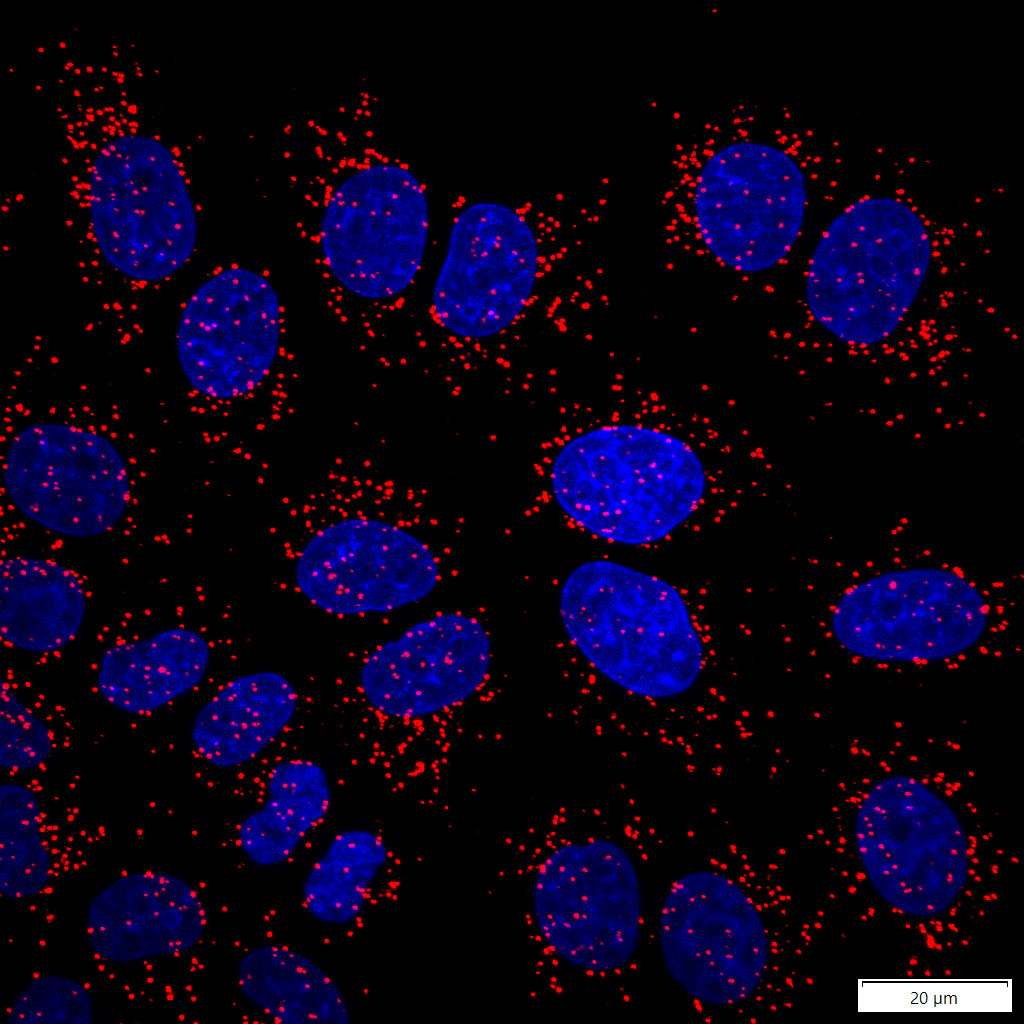

Supplement: Supplementary file 5 — Source data Fig. 3 [file 44319_2024_204_MOESM5_ESM.zip › Figure 3/3A/siControl + Insulin.tif]

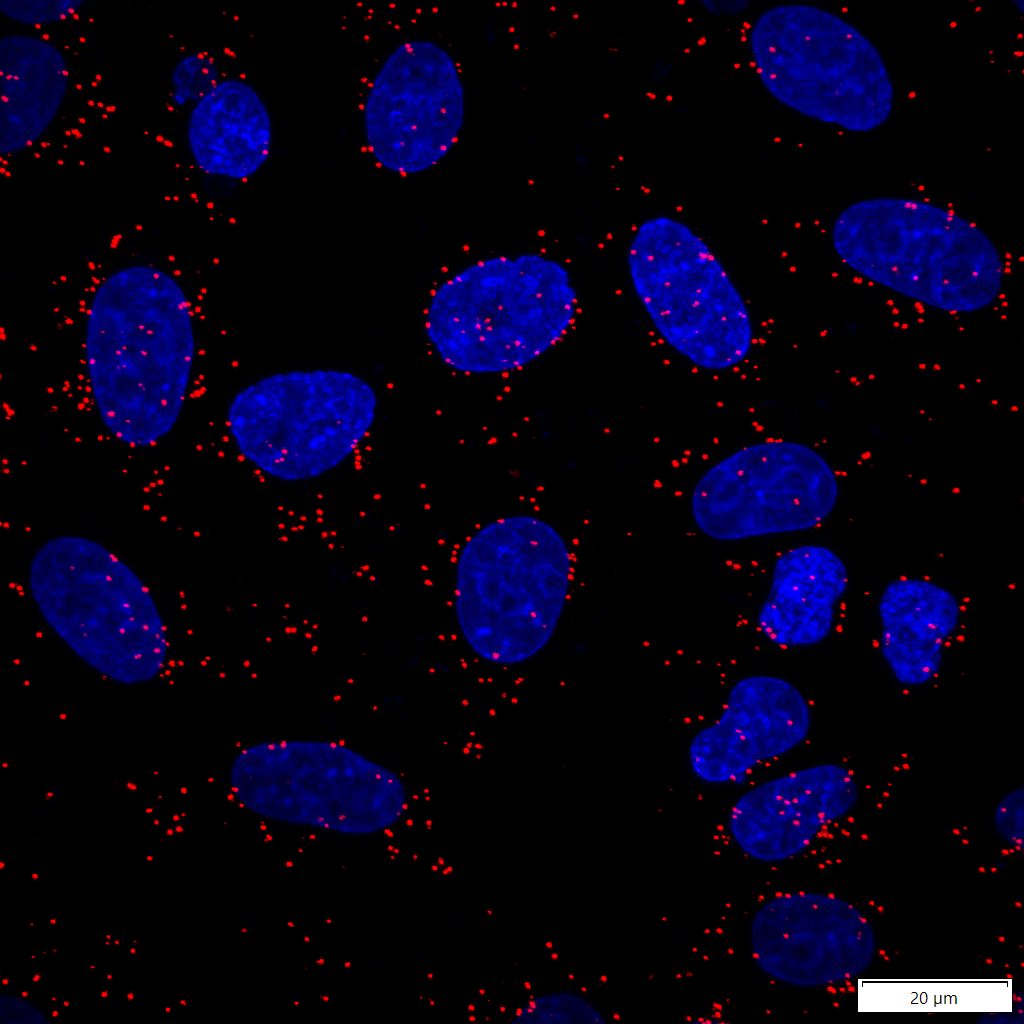

Supplement: Supplementary file 5 — Source data Fig. 3 [file 44319_2024_204_MOESM5_ESM.zip › Figure 3/3A/siRictor + Insulin.tif]

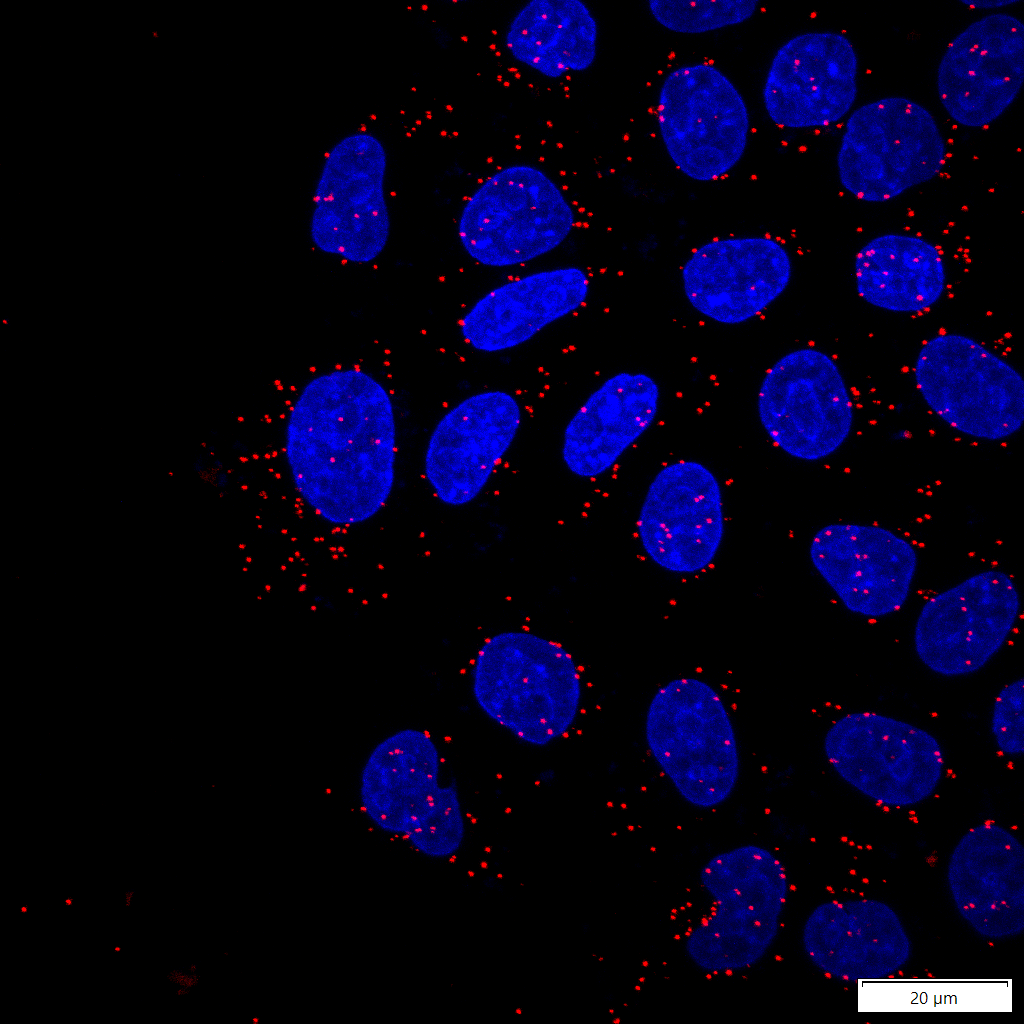

Supplement: Supplementary file 5 — Source data Fig. 3 [file 44319_2024_204_MOESM5_ESM.zip › Figure 3/3A/siSin1 + Insulin.tif]

## Uncropped western blots related to Figure 3B (Left Bottom)

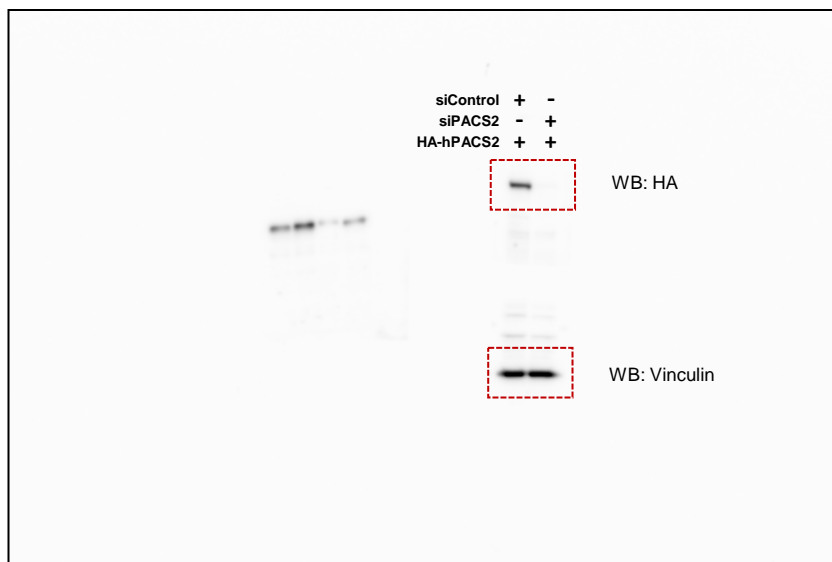

Supplement: Supplementary file 5 — Source data Fig. 3 [file 44319_2024_204_MOESM5_ESM.zip › Figure 3/3B/Figure 3B Western Blots (Left Bottom).pdf]

## Uncropped western blots related to Figure 3B (Left Top)

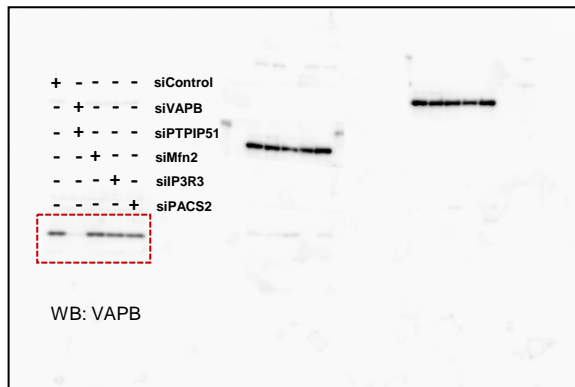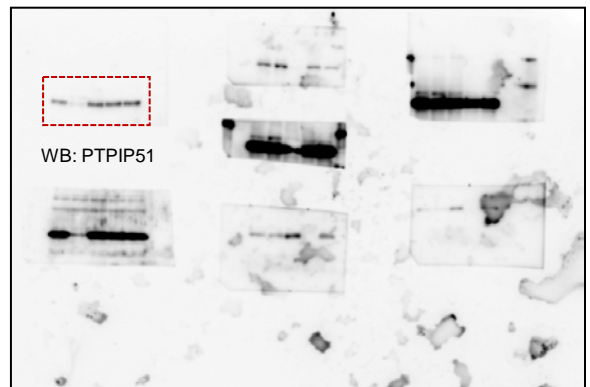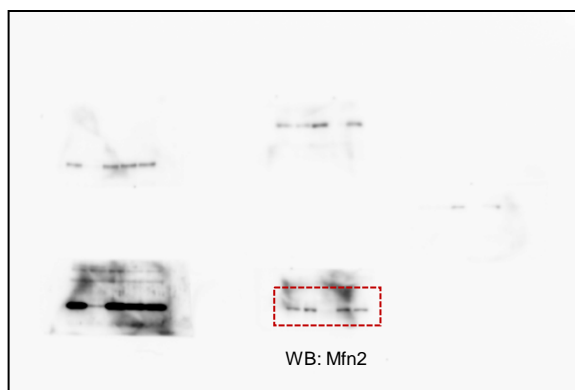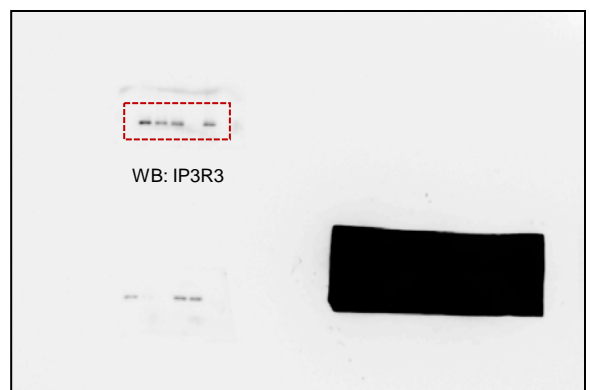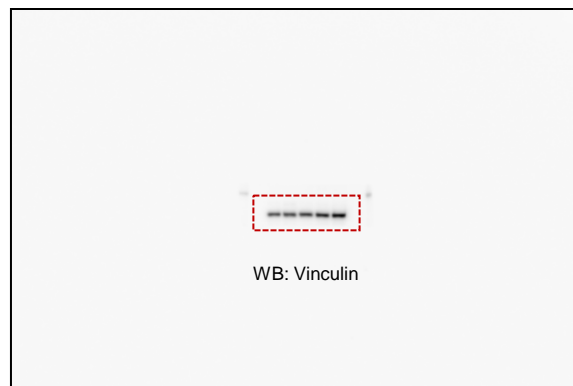

Supplement: Supplementary file 5 — Source data Fig. 3 [file 44319_2024_204_MOESM5_ESM.zip › Figure 3/3B/Figure 3B Western Blots (Left Top).pdf]

Uncropped western blots related to Figure 2E (Right Top)

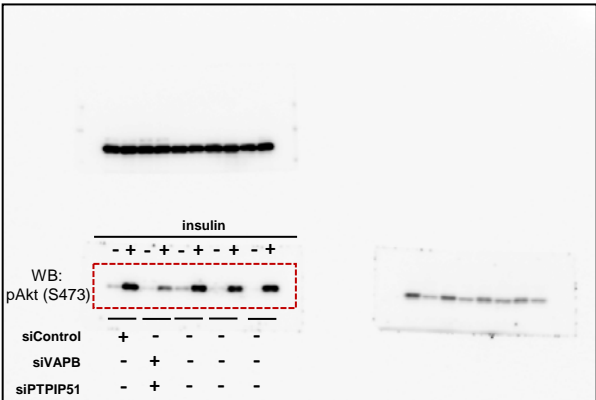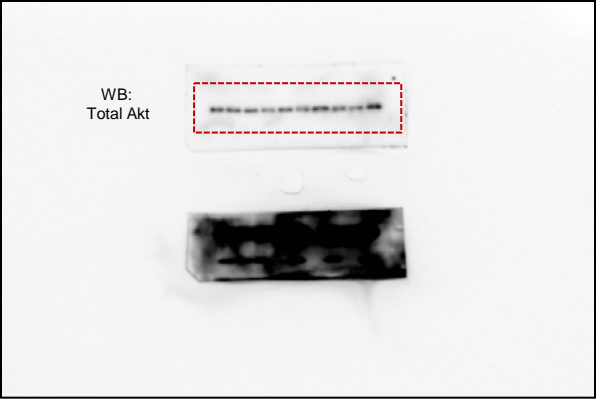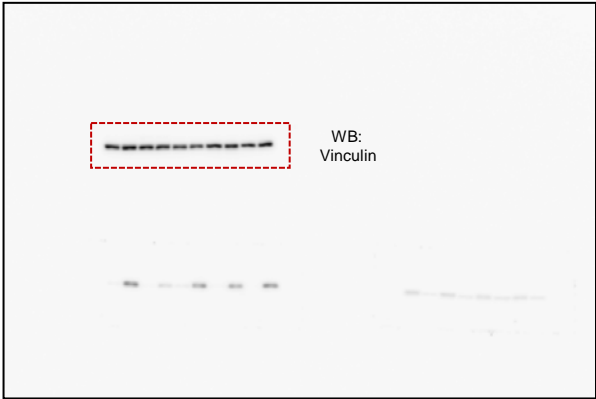

Supplement: Supplementary file 5 — Source data Fig. 3 [file 44319_2024_204_MOESM5_ESM.zip › Figure 3/3B/Figure 3B Western Blots (Right Top).pdf]

Uncropped western blots related to Figure 3C (Left)

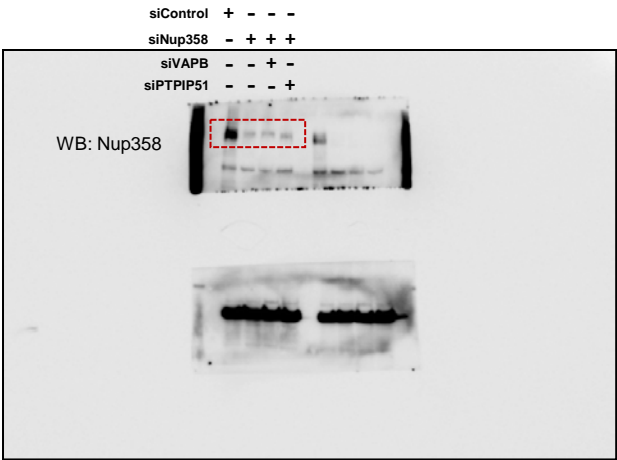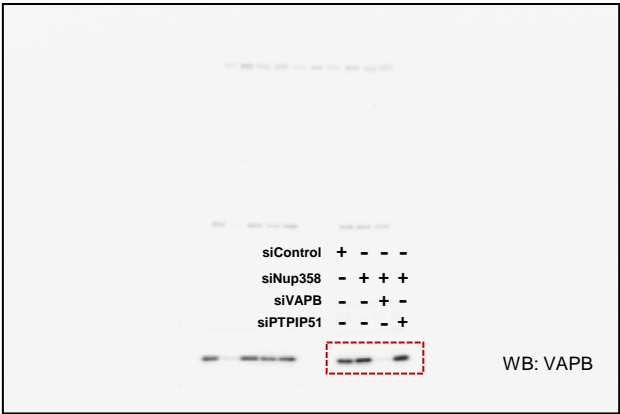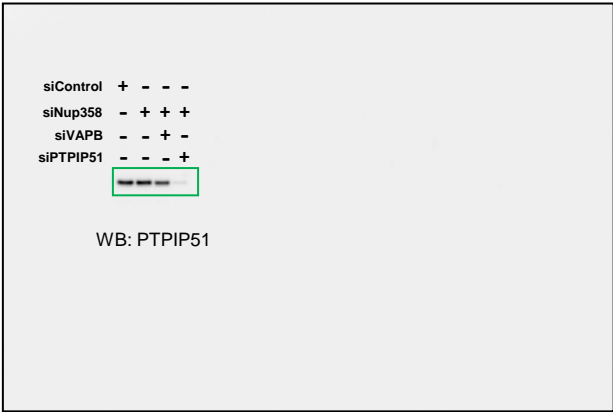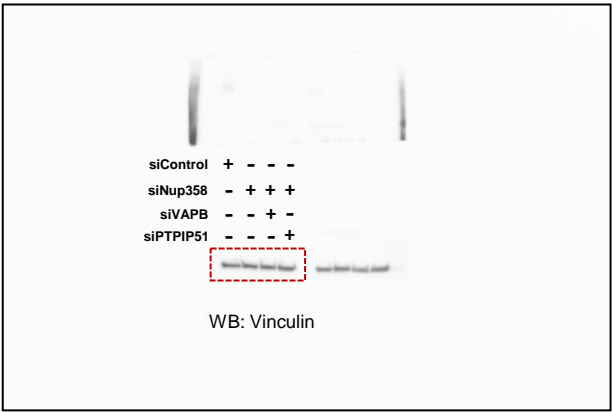

Supplement: Supplementary file 5 — Source data Fig. 3 [file 44319_2024_204_MOESM5_ESM.zip › Figure 3/3C/Figure 3C Western Blots (Left).pdf]

## Uncropped western blots related to Figure 3C (Middle)

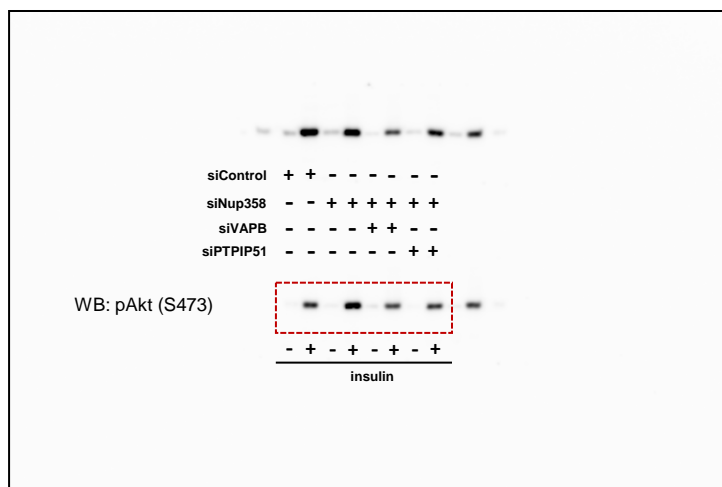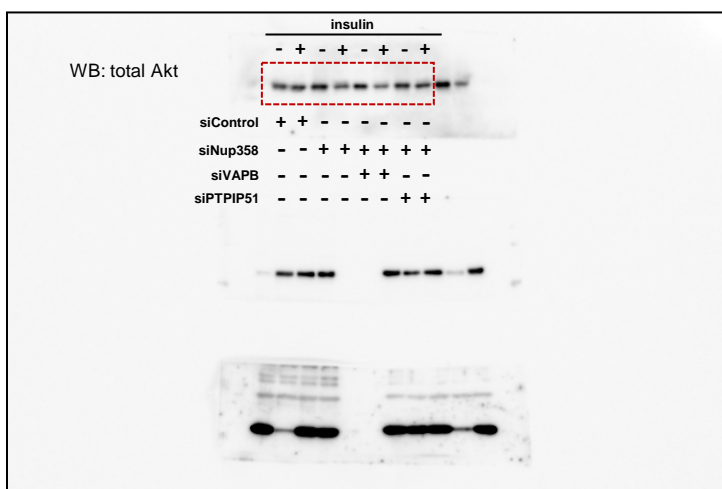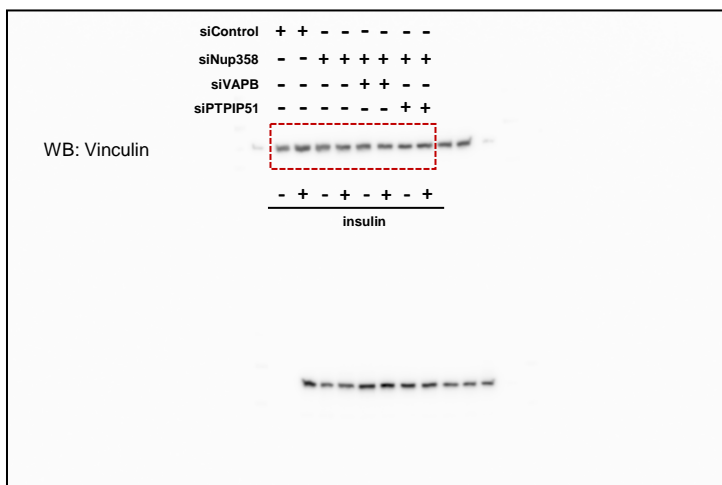

Supplement: Supplementary file 5 — Source data Fig. 3 [file 44319_2024_204_MOESM5_ESM.zip › Figure 3/3C/Figure 3C Western Blots (Middle).pdf]

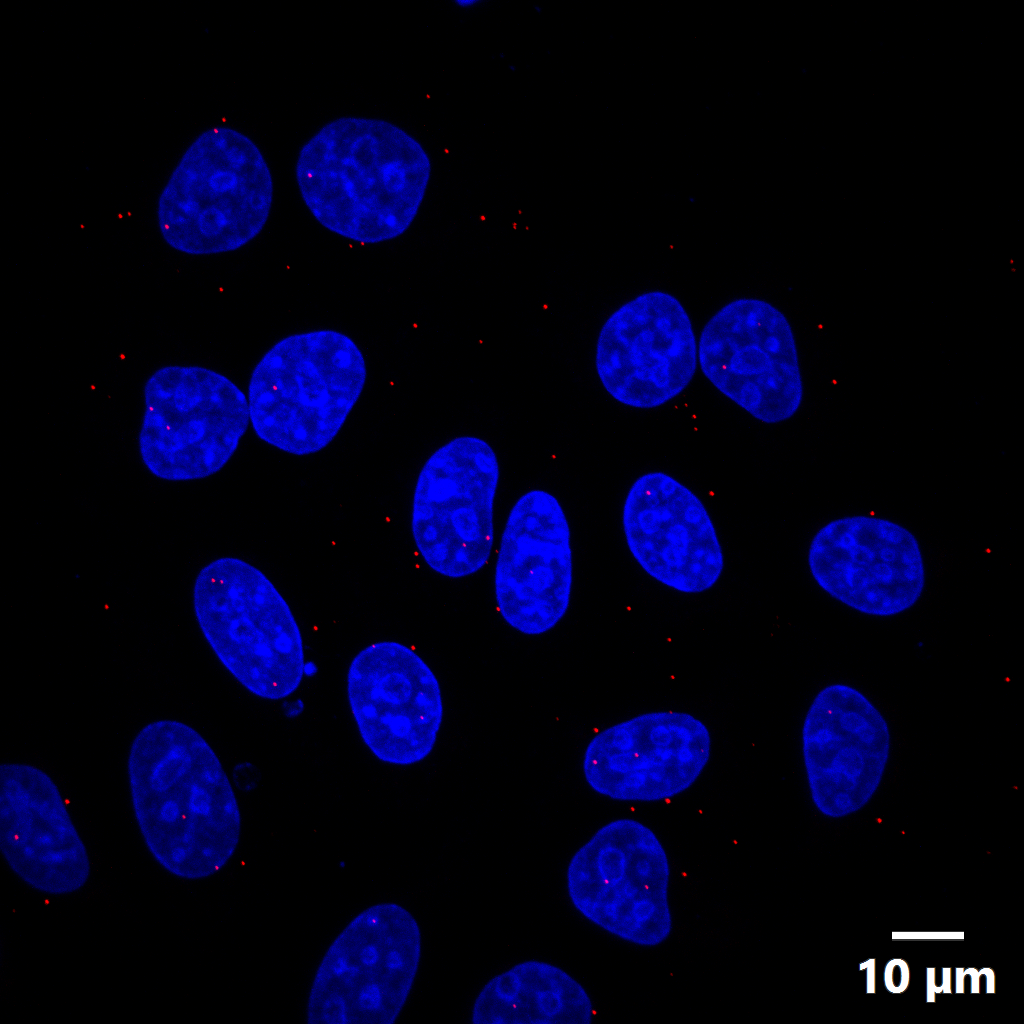

Supplement: Supplementary file 6 — Source data Fig. 4 [file 44319_2024_204_MOESM6_ESM.zip › Figure 4/4A/Figure 4A Right Bottom_Rictor VAPB PLA/Rictor + VAPB PLA image.tif]

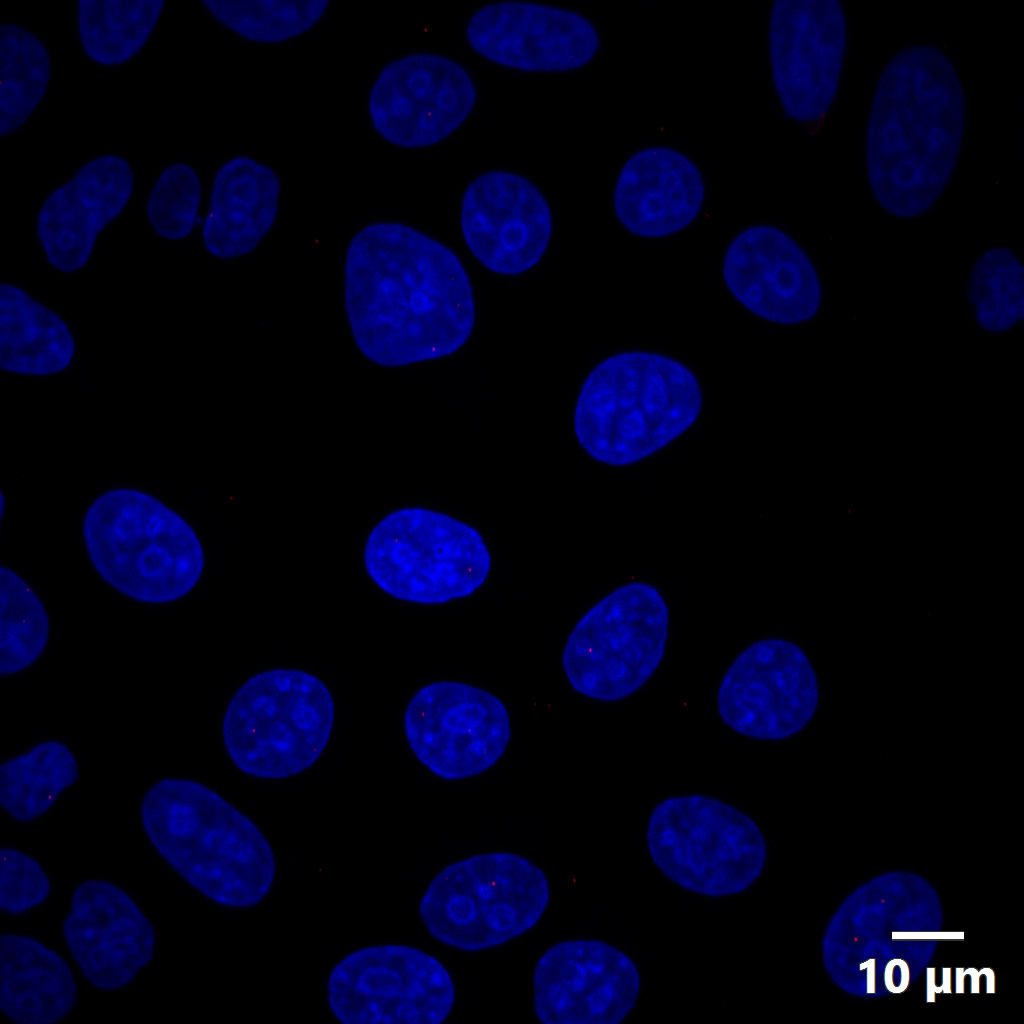

Supplement: Supplementary file 6 — Source data Fig. 4 [file 44319_2024_204_MOESM6_ESM.zip › Figure 4/4A/Figure 4A Right Bottom_Rictor VAPB PLA/Rictor control PLA image.tif]

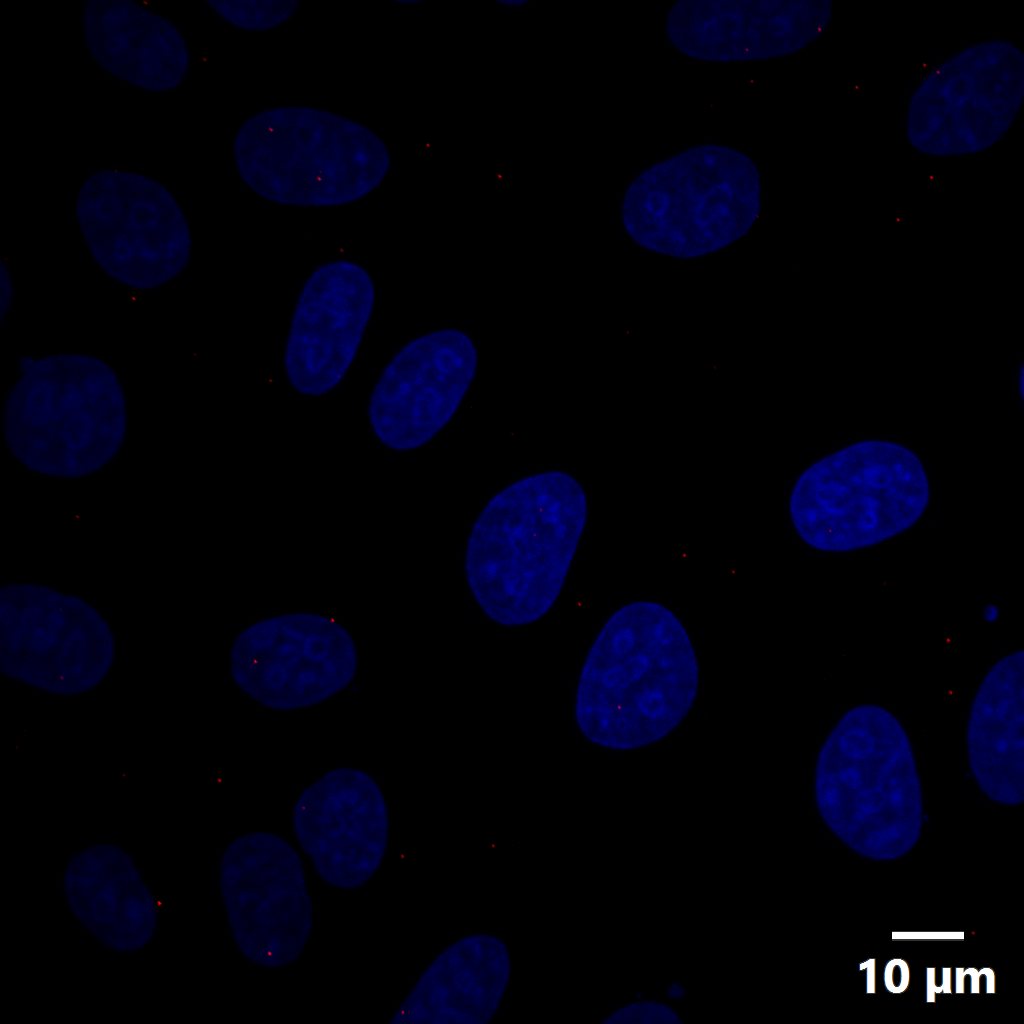

Supplement: Supplementary file 6 — Source data Fig. 4 [file 44319_2024_204_MOESM6_ESM.zip › Figure 4/4A/Figure 4A Right Bottom_Rictor VAPB PLA/VAPB control PLA image.tif]

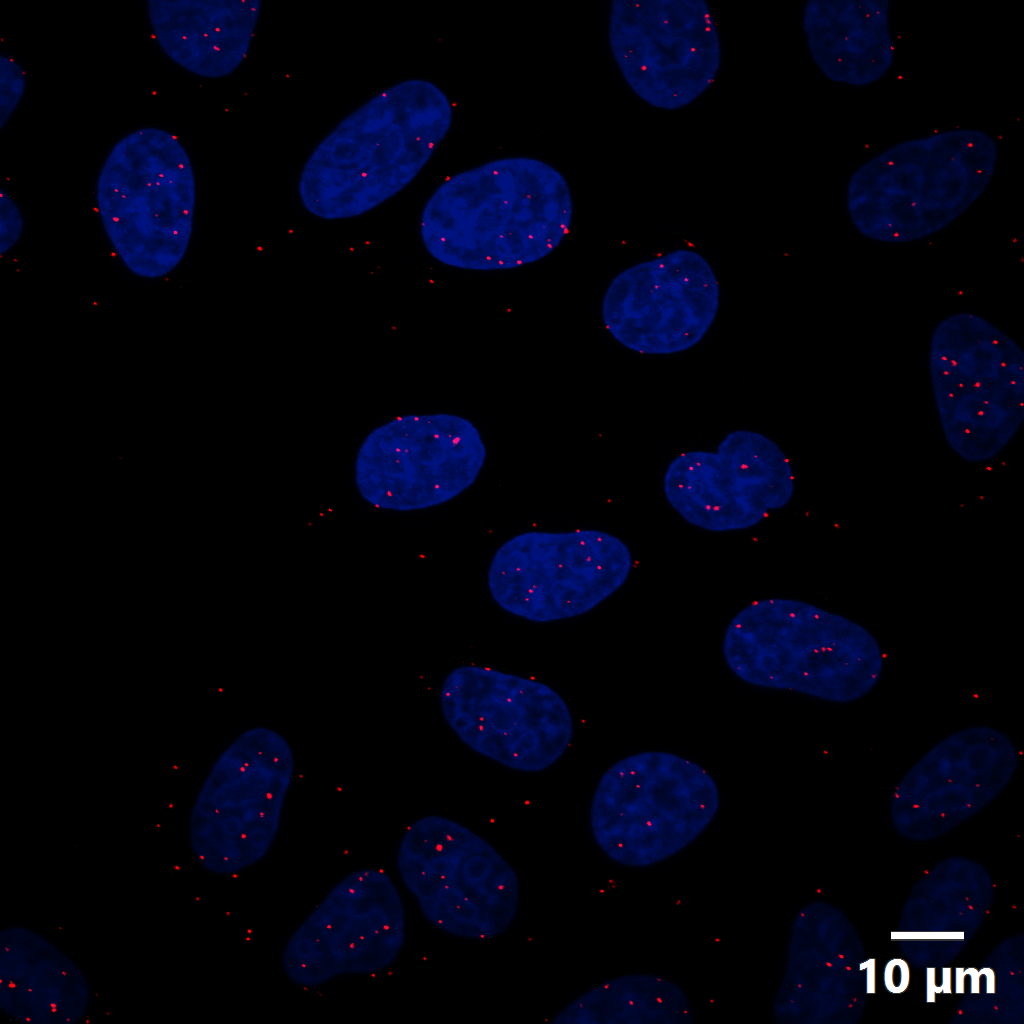

Supplement: Supplementary file 6 — Source data Fig. 4 [file 44319_2024_204_MOESM6_ESM.zip › Figure 4/4A/Figure 4A Right Top_mTOR VAPB PLA/mTOR + VAPB PLA image.tif]

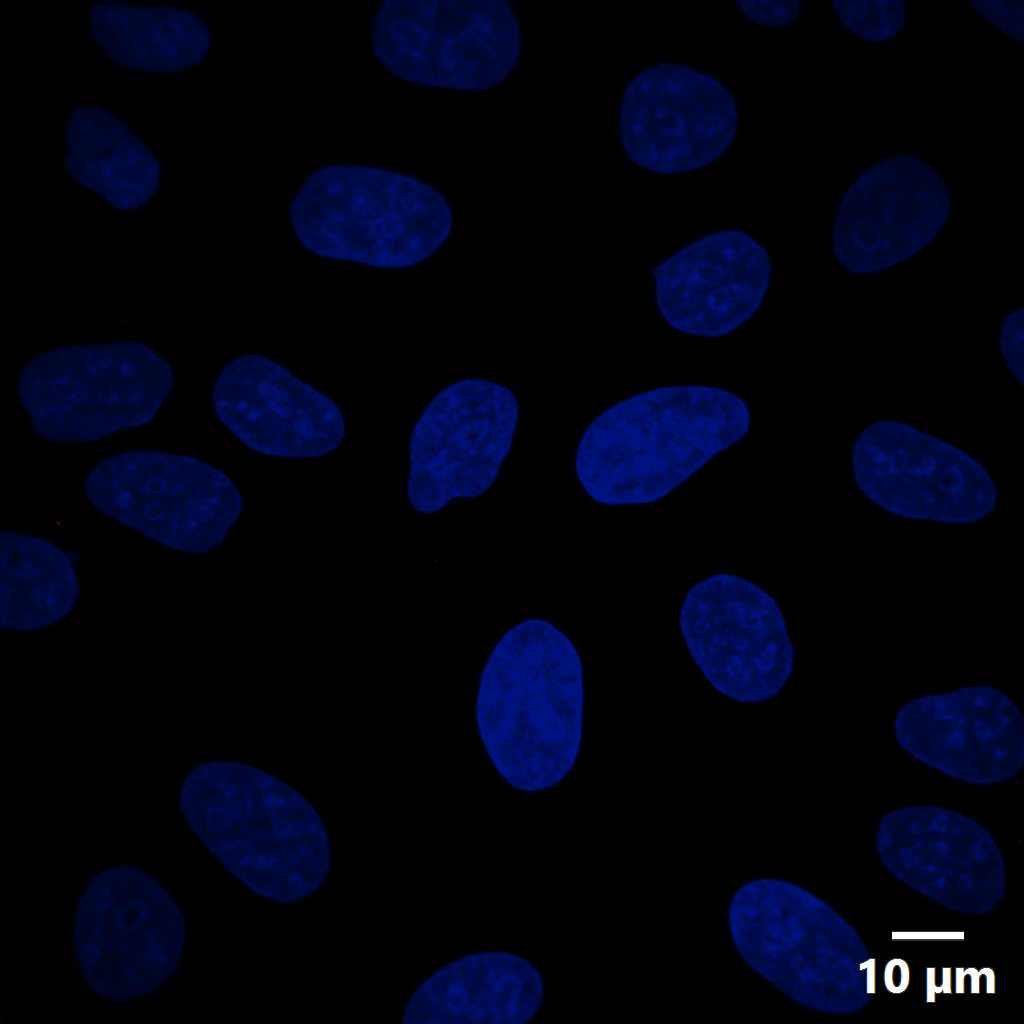

Supplement: Supplementary file 6 — Source data Fig. 4 [file 44319_2024_204_MOESM6_ESM.zip › Figure 4/4A/Figure 4A Right Top_mTOR VAPB PLA/mTOR control PLA image.tif]

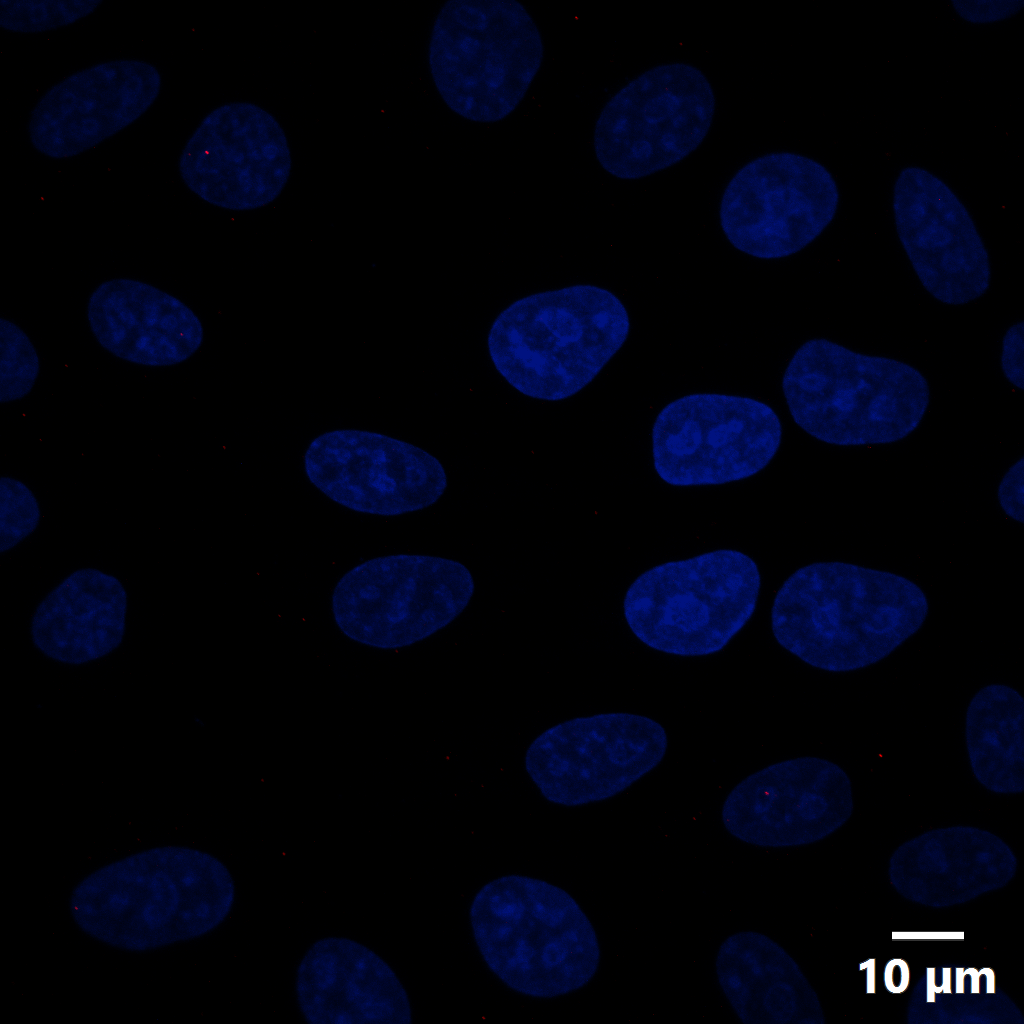

Supplement: Supplementary file 6 — Source data Fig. 4 [file 44319_2024_204_MOESM6_ESM.zip › Figure 4/4A/Figure 4A Right Top_mTOR VAPB PLA/VAPB control PLA image.tif]

## Uncropped western blots related to Figure 4A (Left)

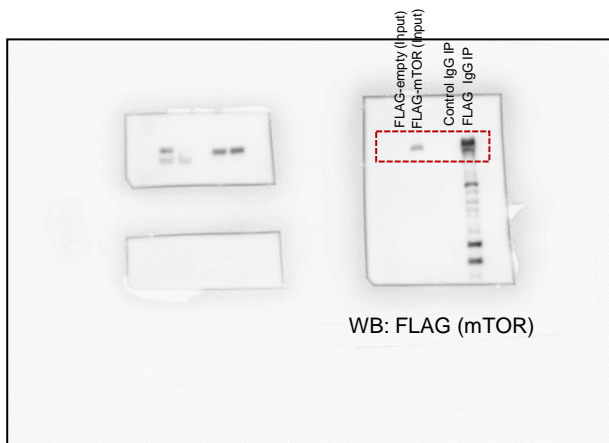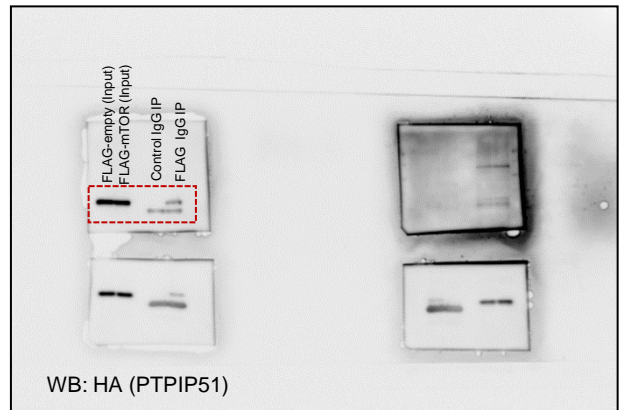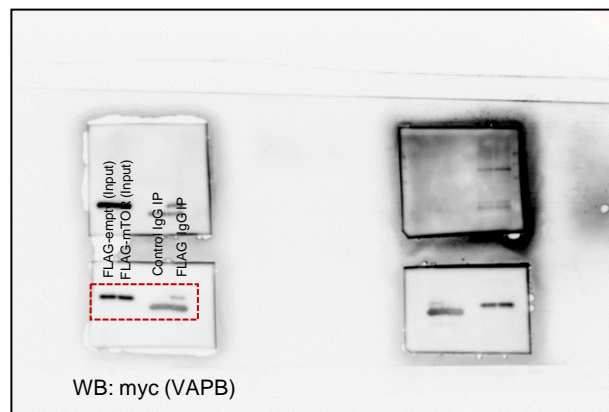

Supplement: Supplementary file 6 — Source data Fig. 4 [file 44319_2024_204_MOESM6_ESM.zip › Figure 4/4A/Figure 4A Western Blots (Left).pdf]

Uncropped western blots related to Figure 4B (Left)

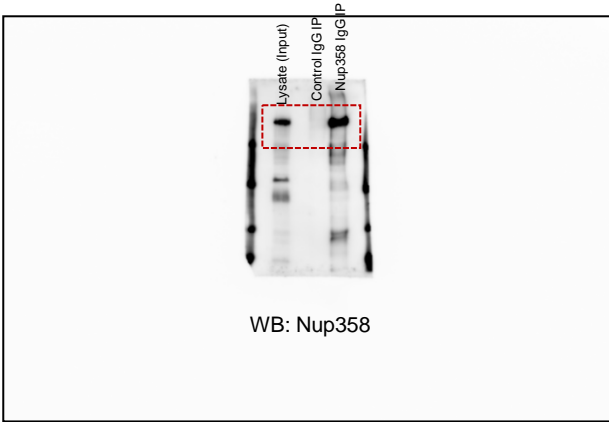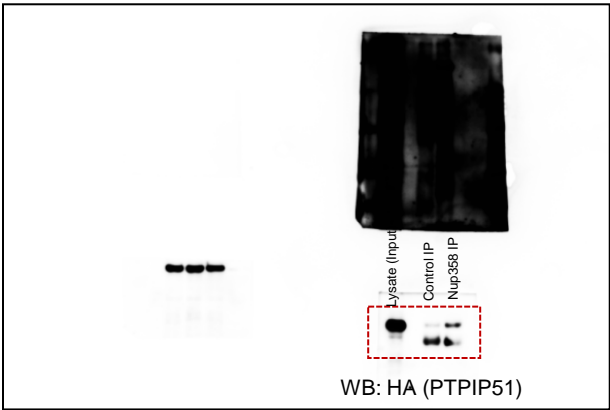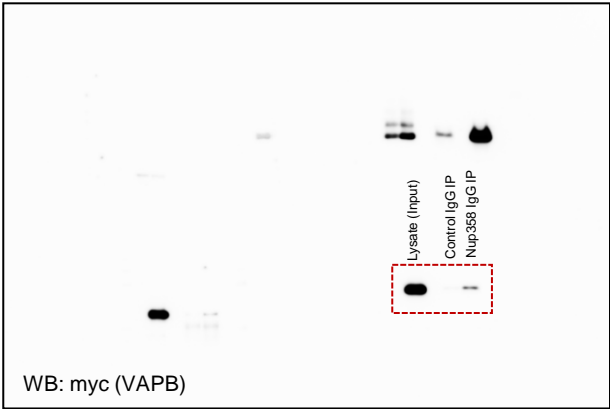

Supplement: Supplementary file 6 — Source data Fig. 4 [file 44319_2024_204_MOESM6_ESM.zip › Figure 4/4B/Figure 4B Western Blots (Left).pdf]

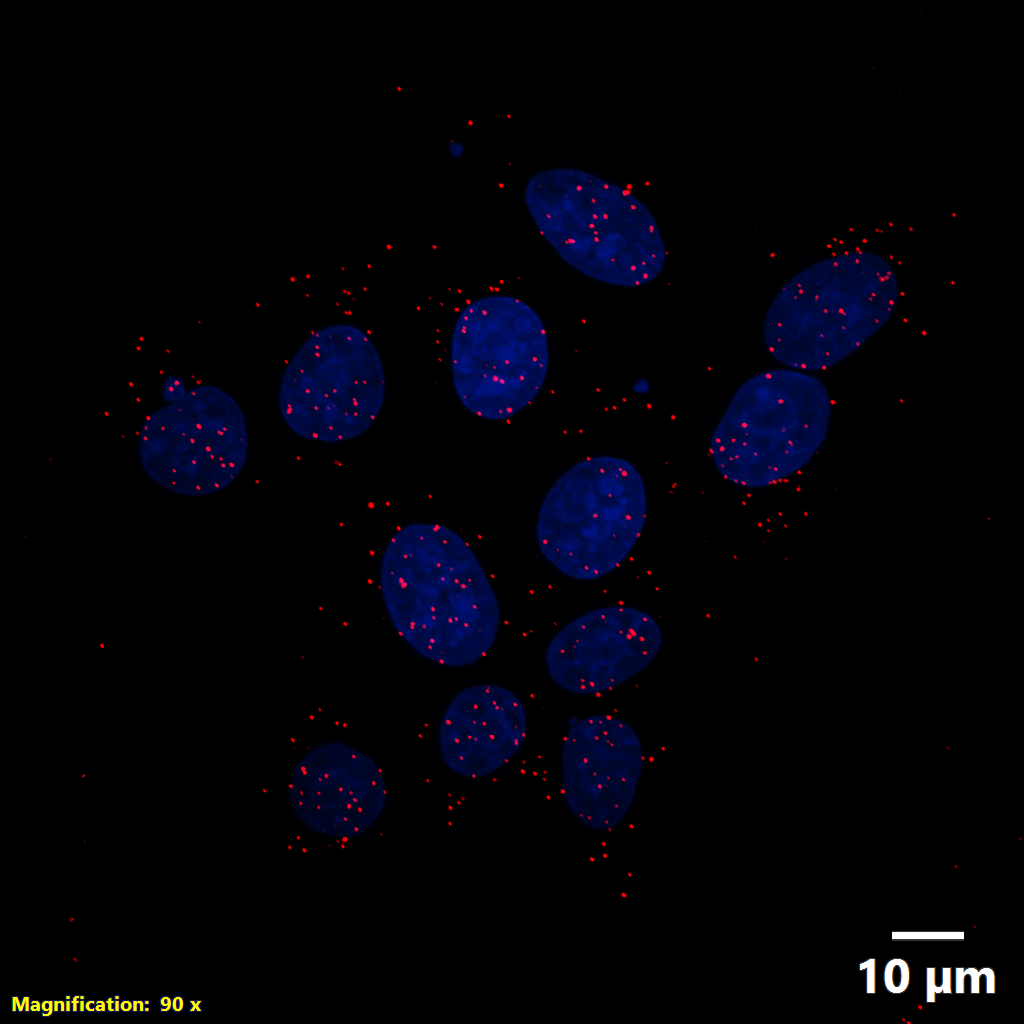

Supplement: Supplementary file 6 — Source data Fig. 4 [file 44319_2024_204_MOESM6_ESM.zip › Figure 4/4B/Nup358 + VAPB PLA image.tif]

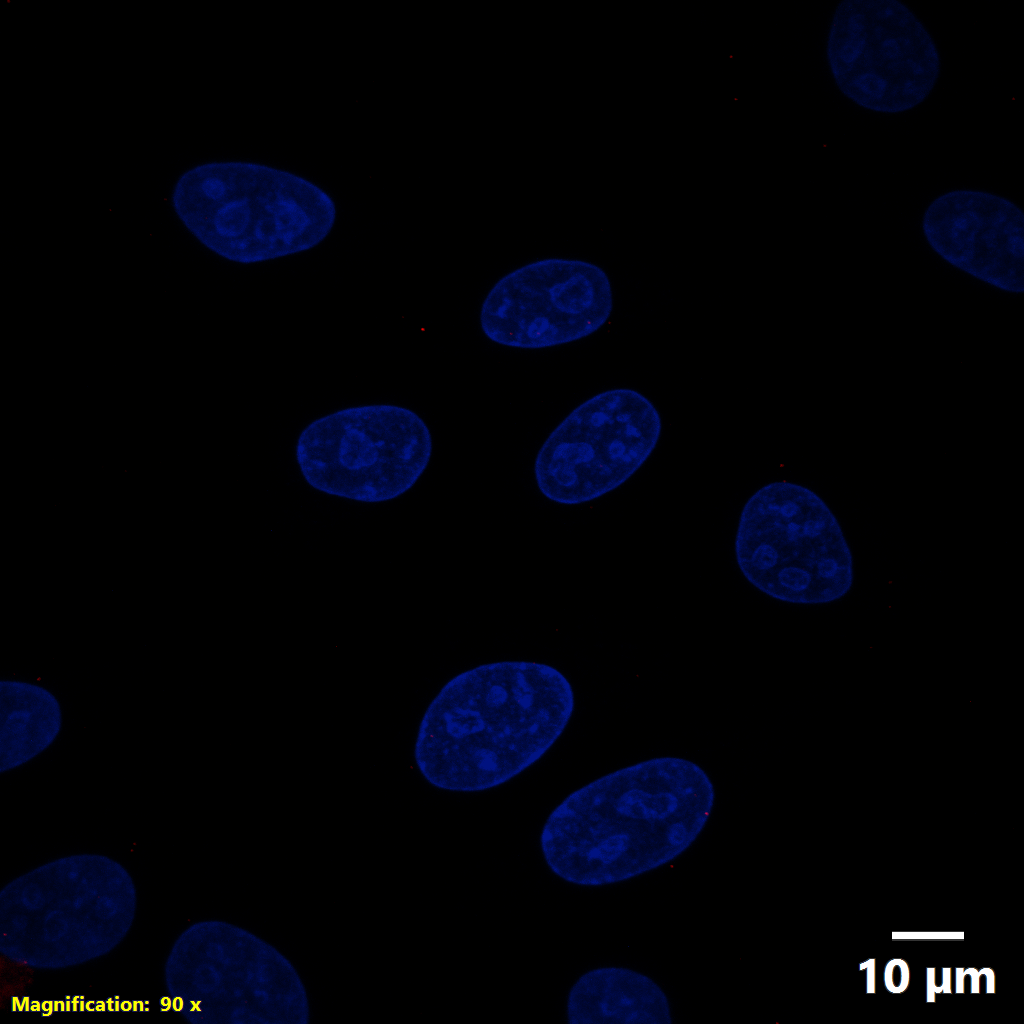

Supplement: Supplementary file 6 — Source data Fig. 4 [file 44319_2024_204_MOESM6_ESM.zip › Figure 4/4B/Nup358 control PLA image.tif]

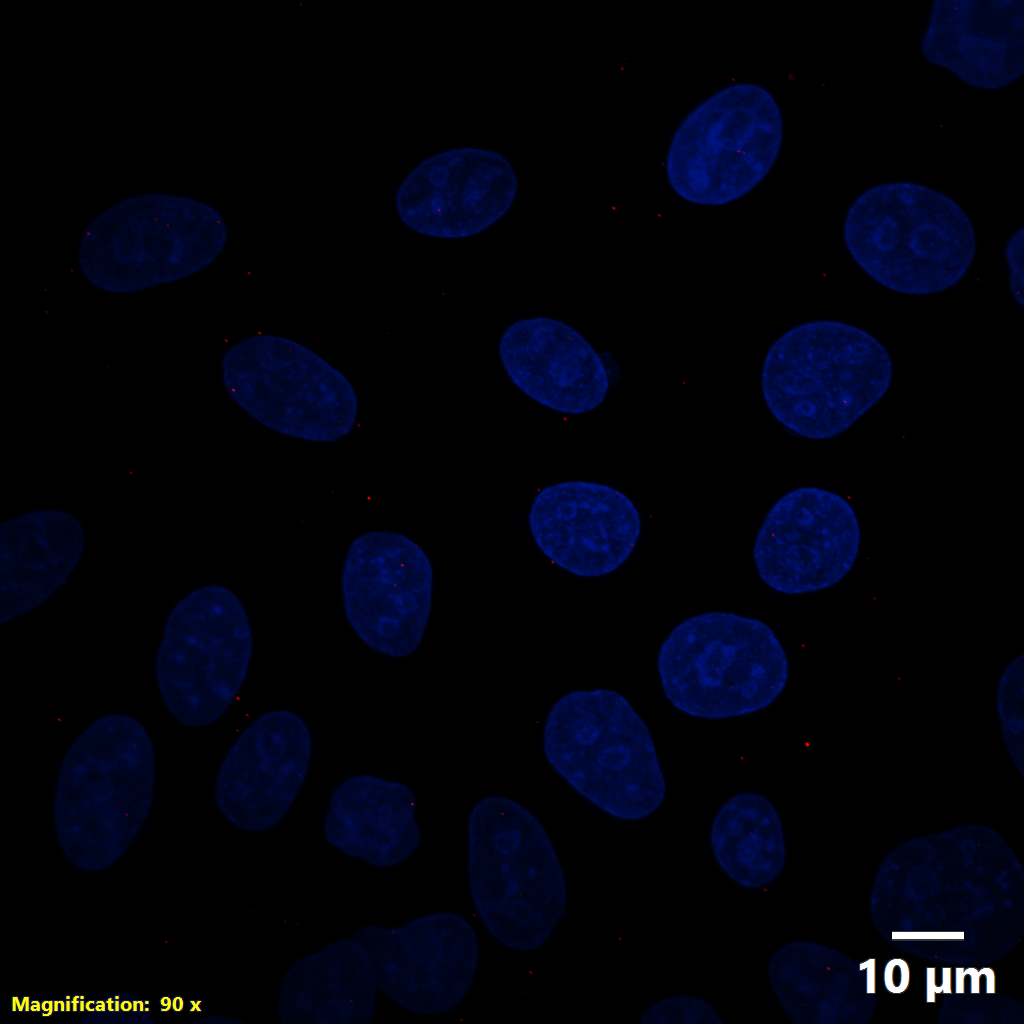

Supplement: Supplementary file 6 — Source data Fig. 4 [file 44319_2024_204_MOESM6_ESM.zip › Figure 4/4B/VAPB control PLA image.tif]

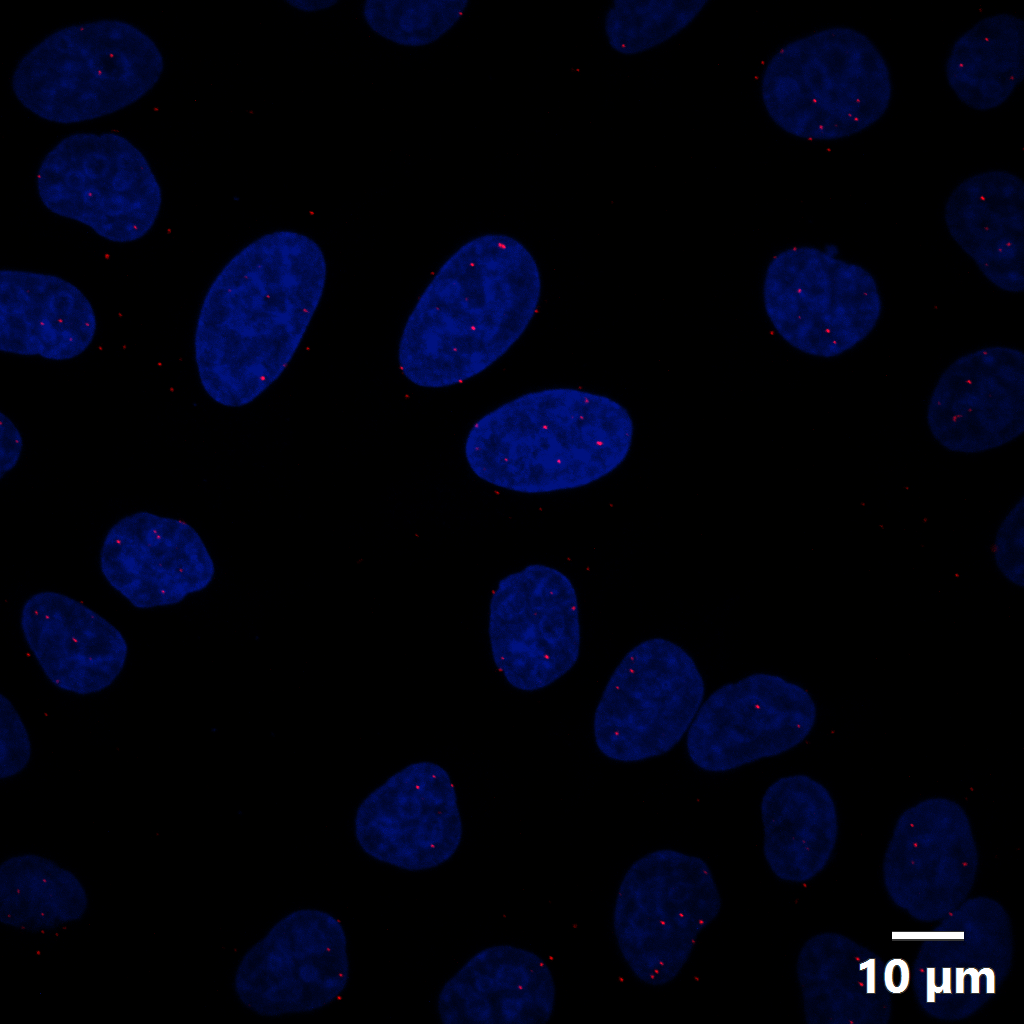

Supplement: Supplementary file 6 — Source data Fig. 4 [file 44319_2024_204_MOESM6_ESM.zip › Figure 4/4C/Figure 4C VAPB-mTOR PLA data (Second from Left)/- Insulin (VAPB + mTOR) PLA image.tif]

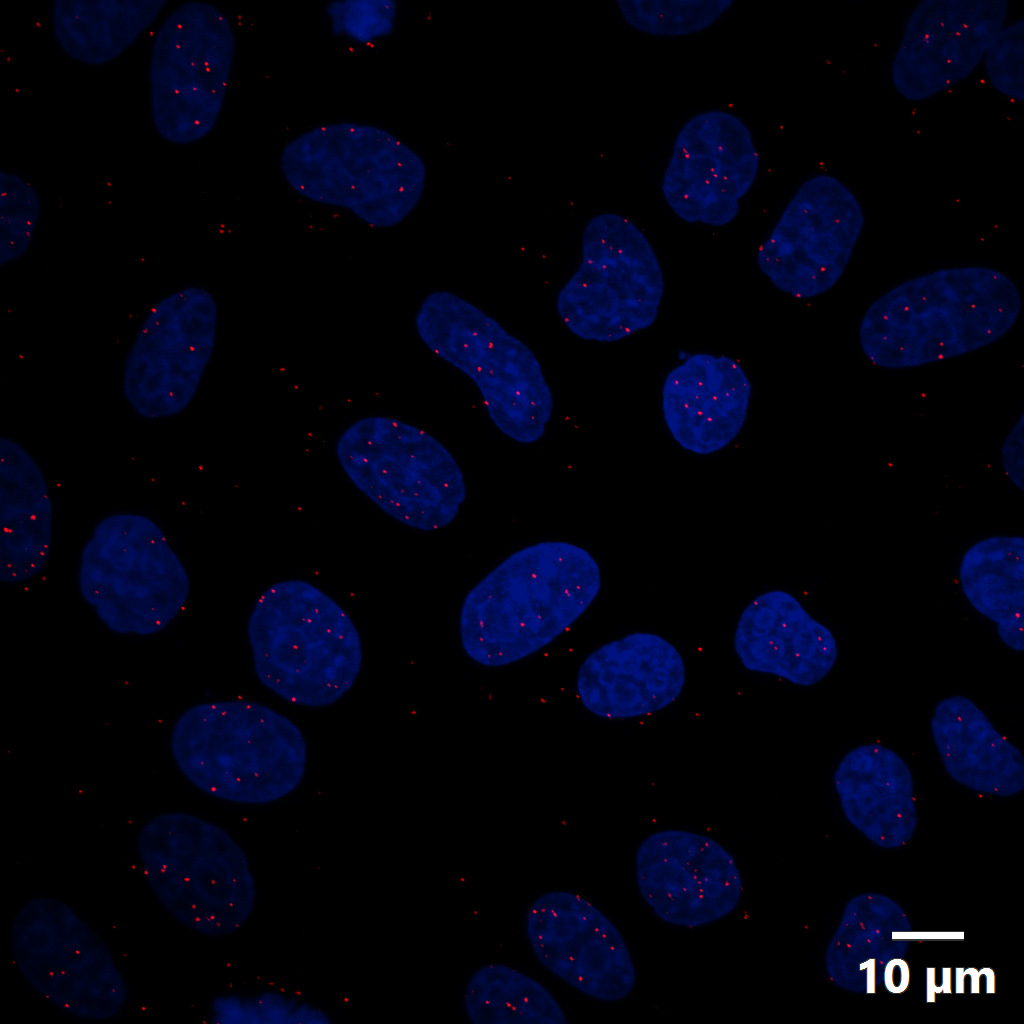

Supplement: Supplementary file 6 — Source data Fig. 4 [file 44319_2024_204_MOESM6_ESM.zip › Figure 4/4C/Figure 4C VAPB-mTOR PLA data (Second from Left)/+ Insulin (VAPB + mTOR) PLA image.tif]

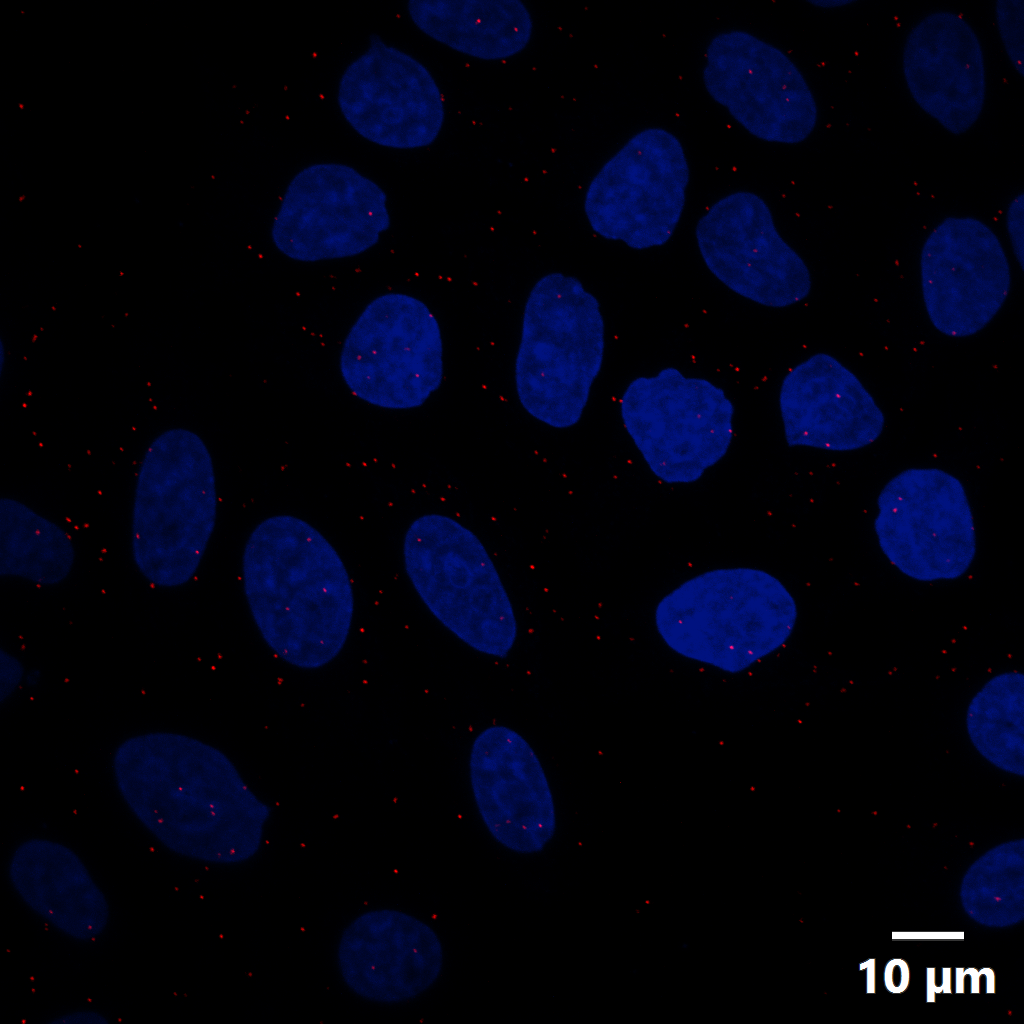

Supplement: Supplementary file 6 — Source data Fig. 4 [file 44319_2024_204_MOESM6_ESM.zip › Figure 4/4C/Figure 4C VAPB-Nup358 PLA data (First from Left)/- Insulin (VAPB + Nup358 PLA).tif]

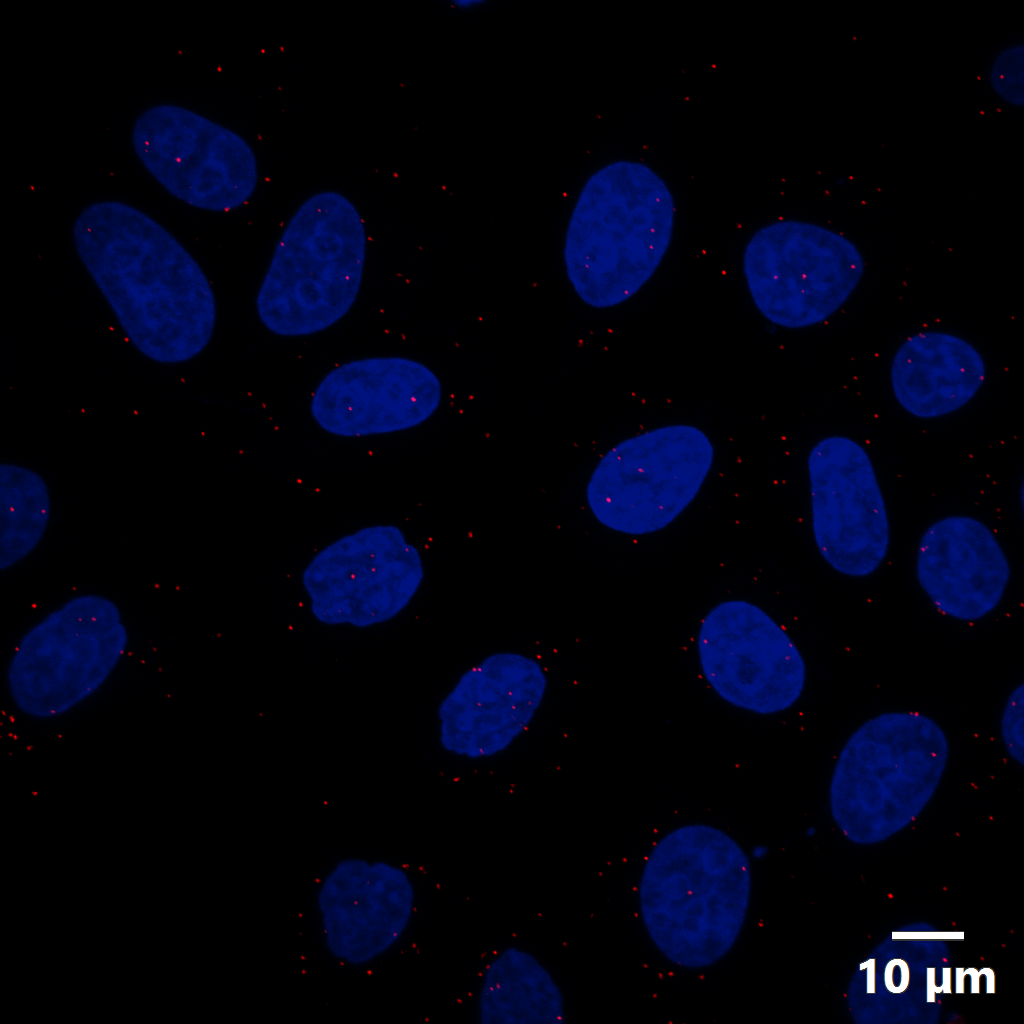

Supplement: Supplementary file 6 — Source data Fig. 4 [file 44319_2024_204_MOESM6_ESM.zip › Figure 4/4C/Figure 4C VAPB-Nup358 PLA data (First from Left)/+ Insulin (VAPB + Nup358 PLA).tif]

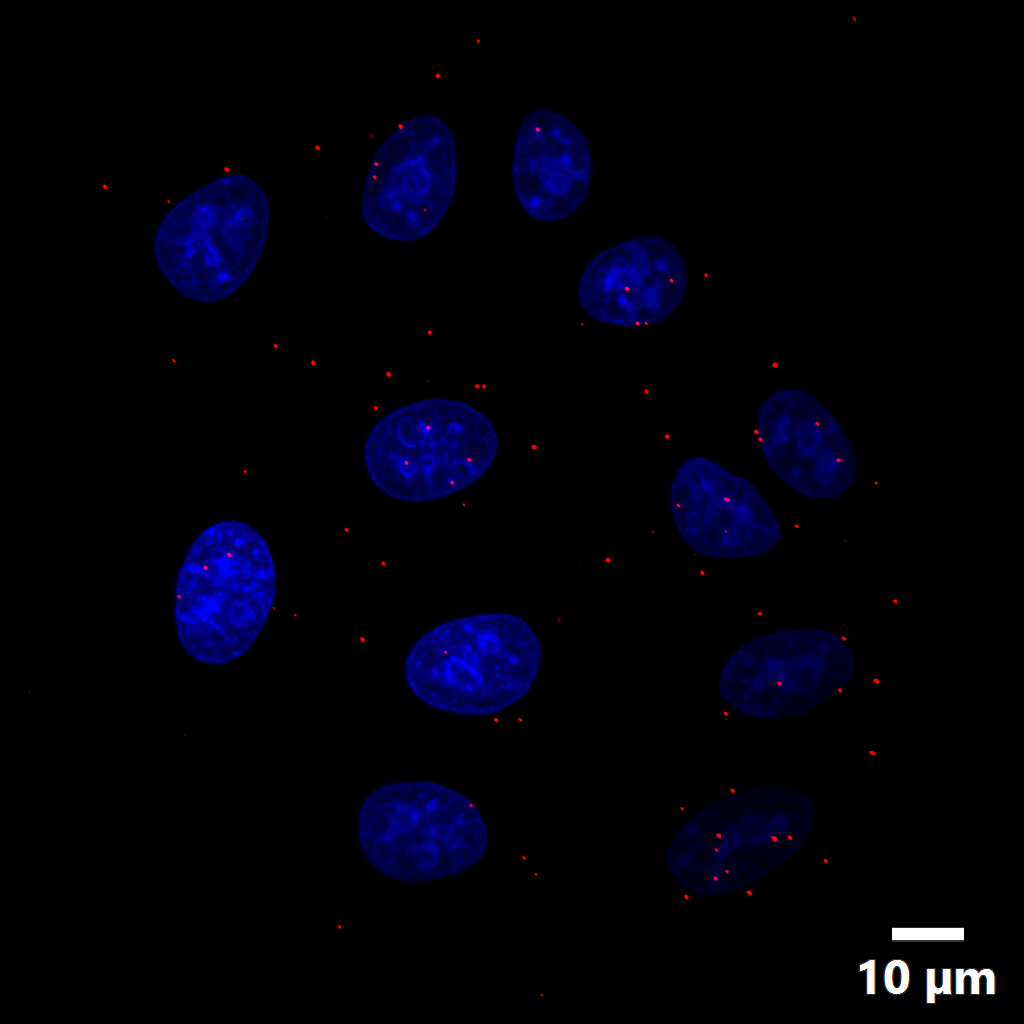

Supplement: Supplementary file 6 — Source data Fig. 4 [file 44319_2024_204_MOESM6_ESM.zip › Figure 4/4C/Figure 4C VAPB-Rictor PLA data (Third from Left)/- Insulin (VAPB+Rictor) PLA image.tif]

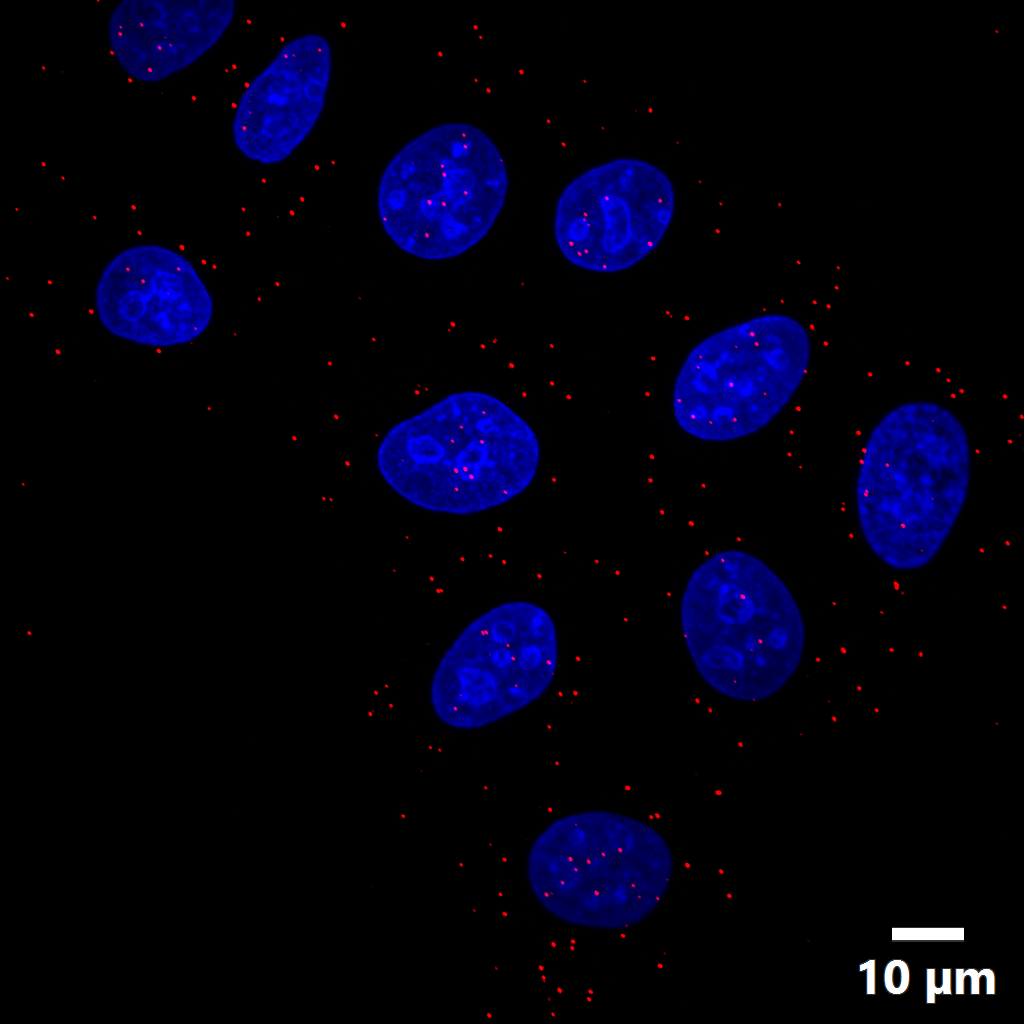

Supplement: Supplementary file 6 — Source data Fig. 4 [file 44319_2024_204_MOESM6_ESM.zip › Figure 4/4C/Figure 4C VAPB-Rictor PLA data (Third from Left)/+ Insulin (VAPB+Rictor) PLA image.tif]

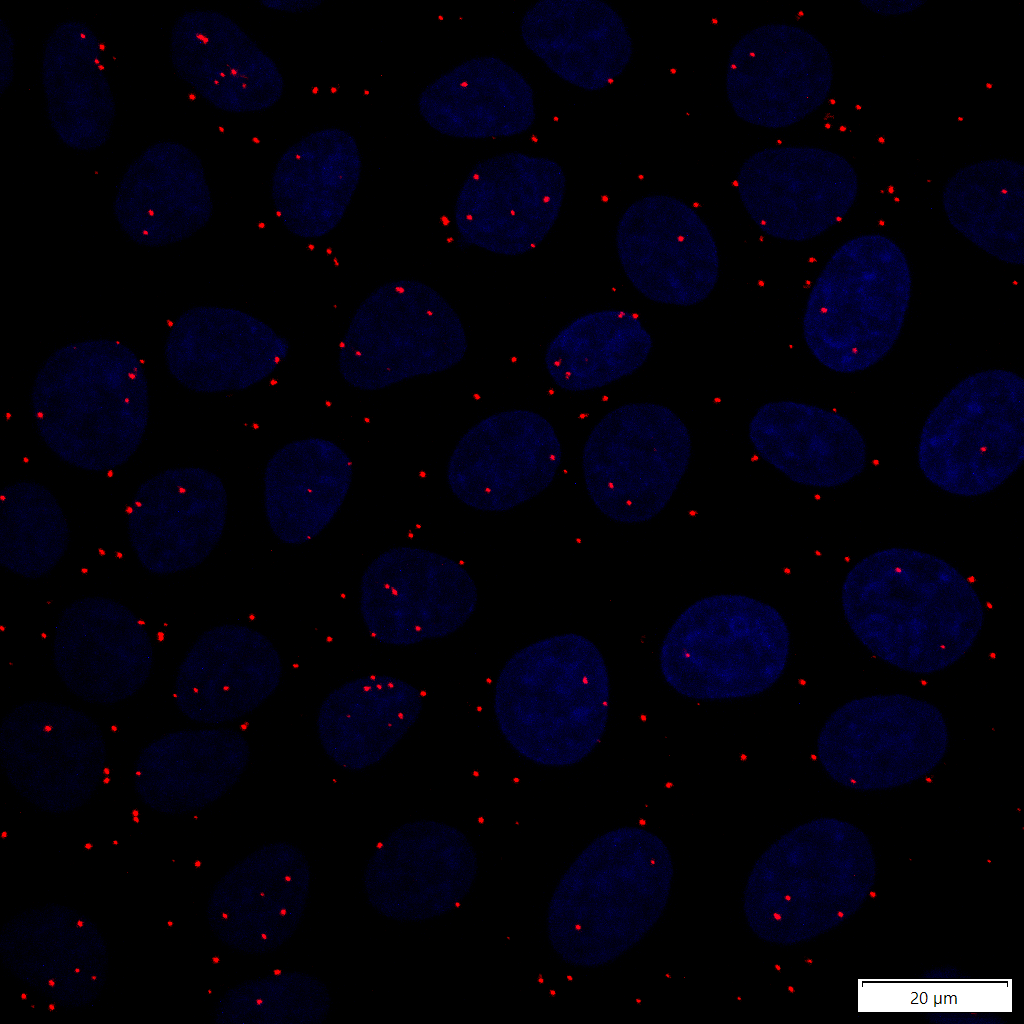

Supplement: Supplementary file 6 — Source data Fig. 4 [file 44319_2024_204_MOESM6_ESM.zip › Figure 4/4C/Figure 4C VAPB-Sin1 PLA data (Fourth from Left)/- Insulin (VAPB+Sin1 PLA) image.tif]

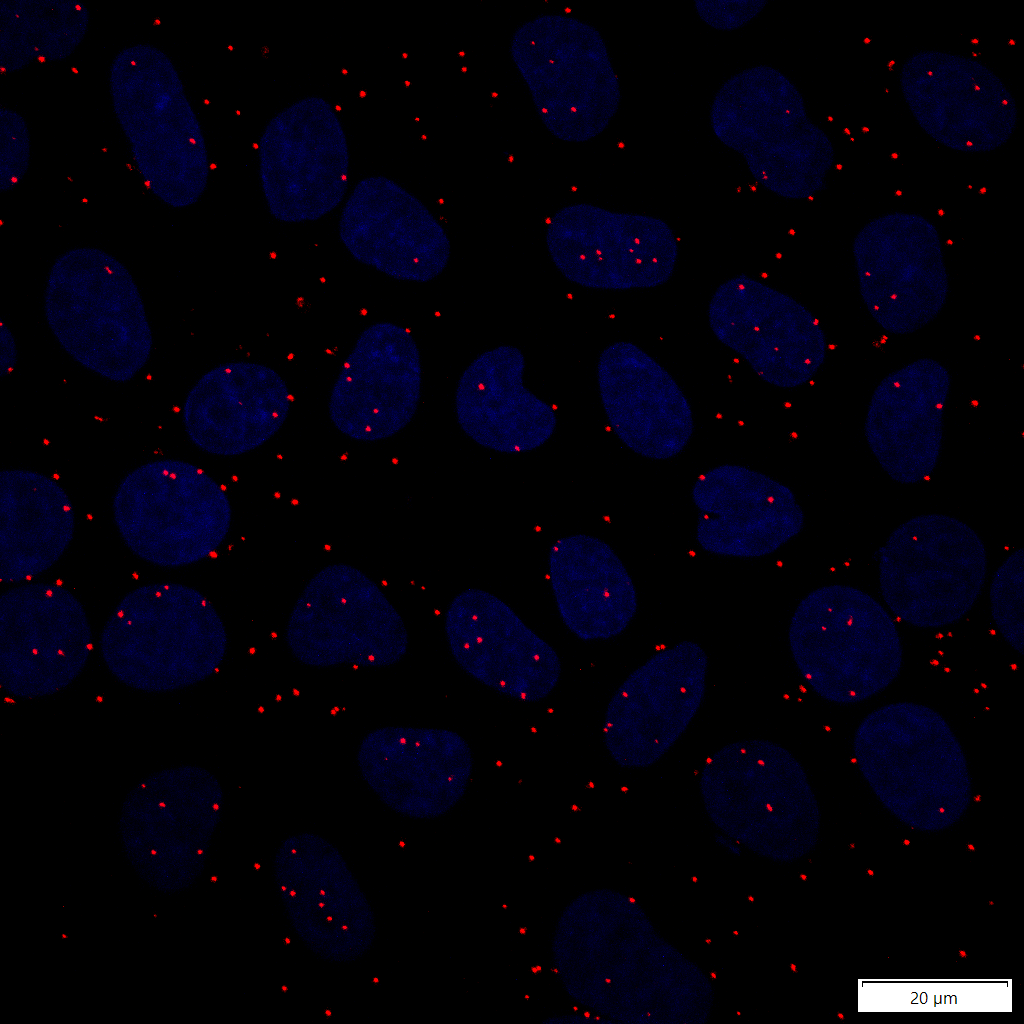

Supplement: Supplementary file 6 — Source data Fig. 4 [file 44319_2024_204_MOESM6_ESM.zip › Figure 4/4C/Figure 4C VAPB-Sin1 PLA data (Fourth from Left)/+ Insulin (VAPB+Sin1 PLA) image.tif]

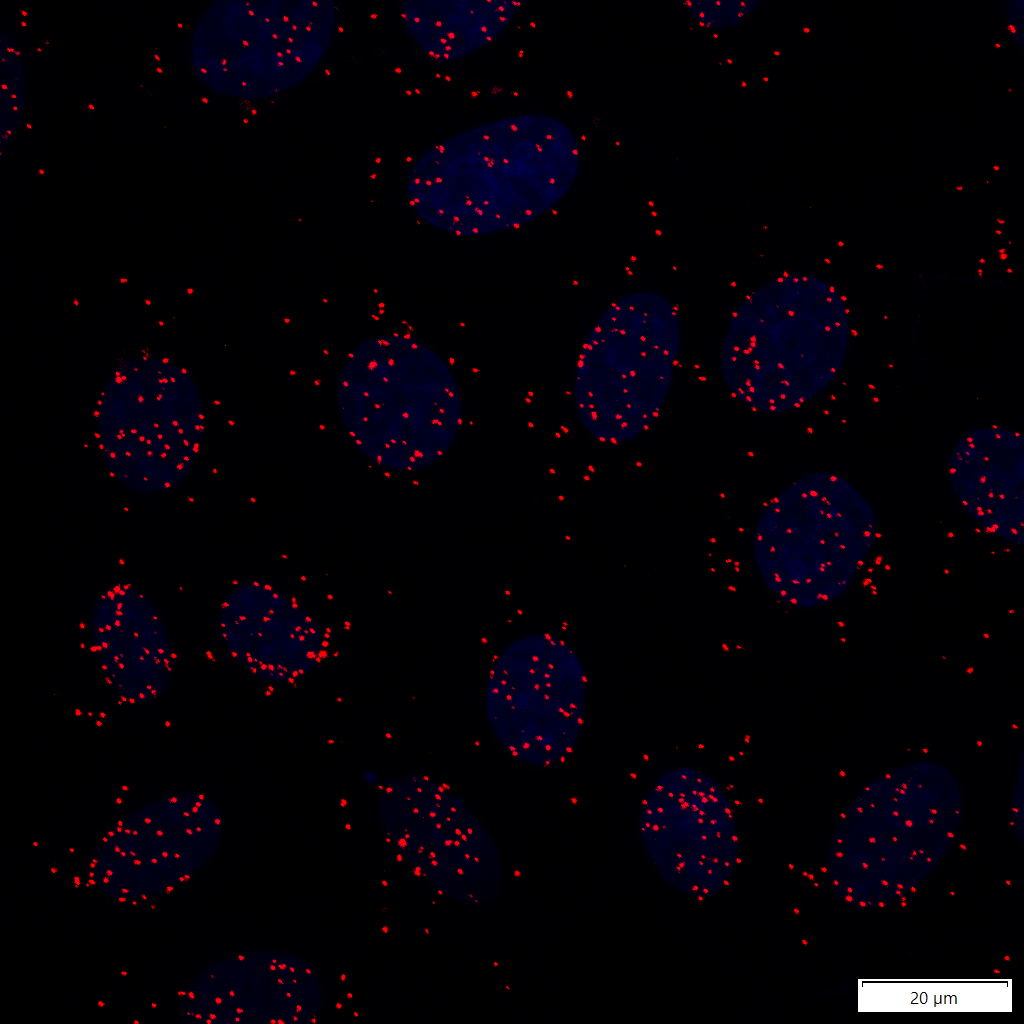

Supplement: Supplementary file 6 — Source data Fig. 4 [file 44319_2024_204_MOESM6_ESM.zip › Figure 4/4D/- Insulin DMSO control (VAPB+PTPIP51 PLA).tif]

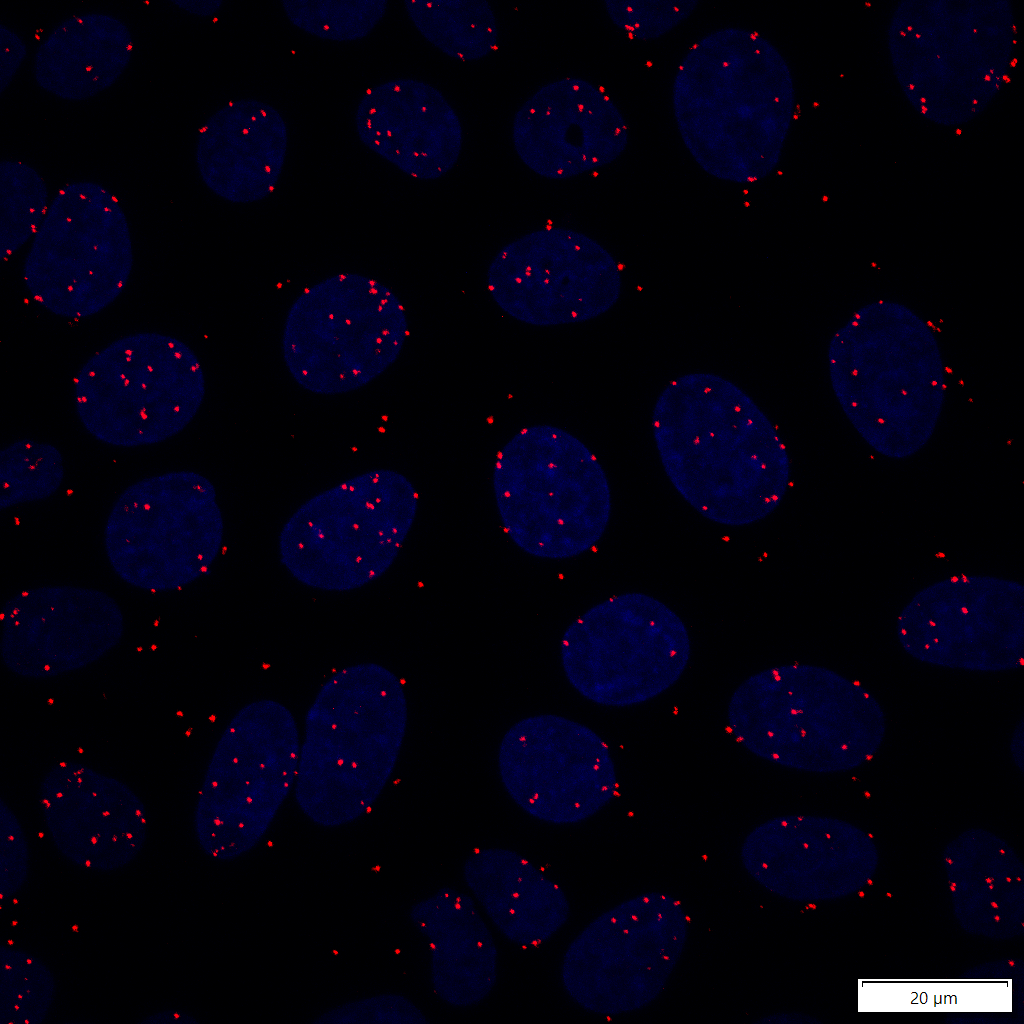

Supplement: Supplementary file 6 — Source data Fig. 4 [file 44319_2024_204_MOESM6_ESM.zip › Figure 4/4D/+ Insulin + Wortmannin (VAPB+PTPIP51 PLA).tif]

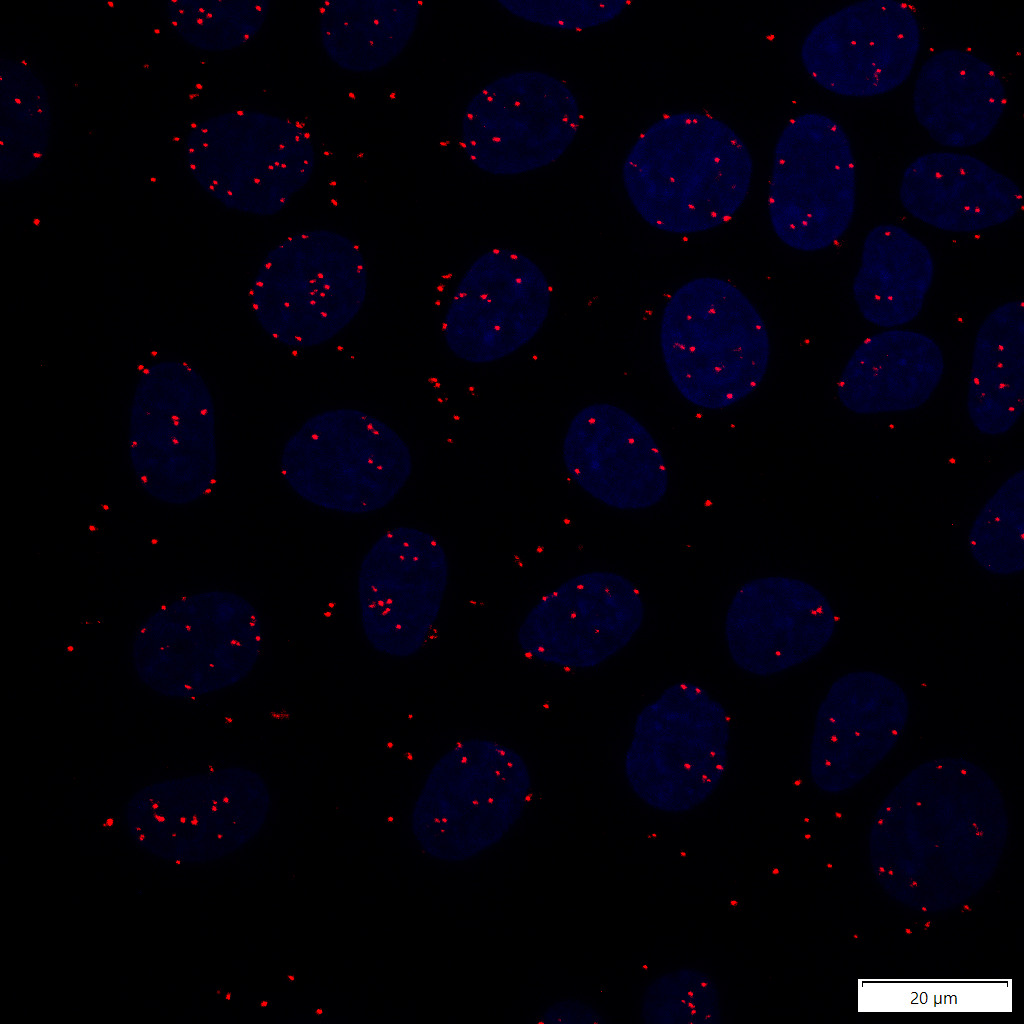

Supplement: Supplementary file 6 — Source data Fig. 4 [file 44319_2024_204_MOESM6_ESM.zip › Figure 4/4D/+ Insulin DMSO control (VAPB+PTPIP51 PLA).tif]

Uncropped western blots related to Figure 4D (Left)

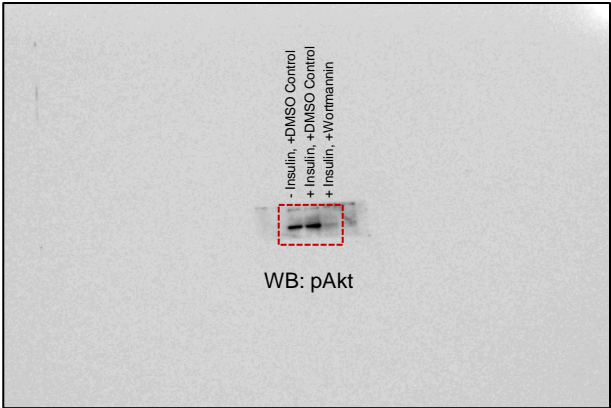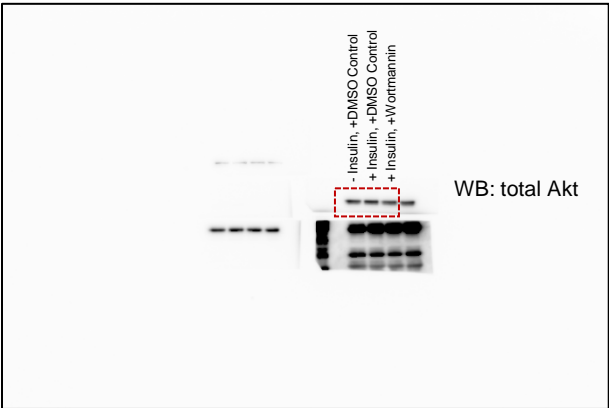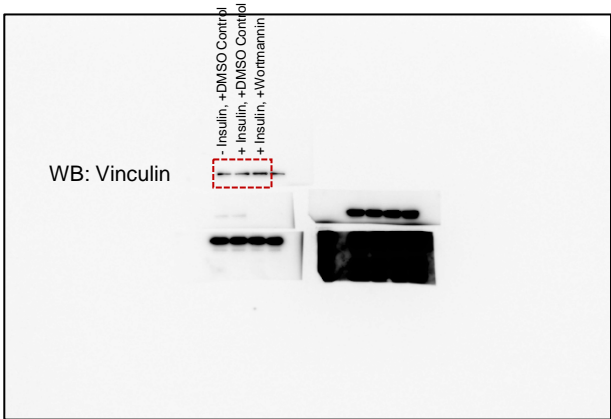

Supplement: Supplementary file 6 — Source data Fig. 4 [file 44319_2024_204_MOESM6_ESM.zip › Figure 4/4D/Figure 4D Western Blots (Left).pdf]

# Uncropped western blots related to Figure 5A (Top)

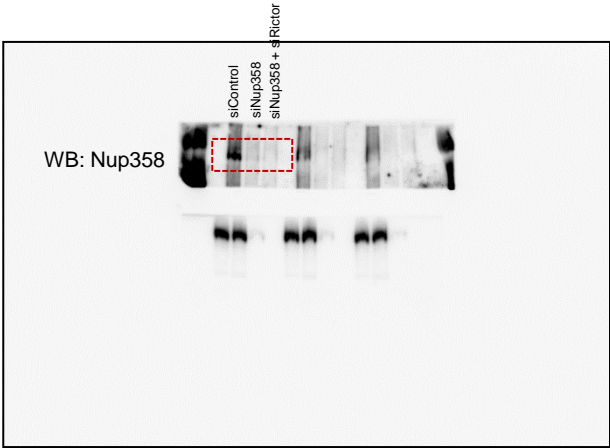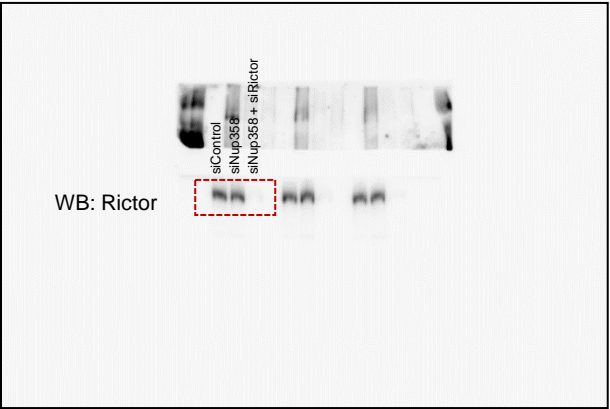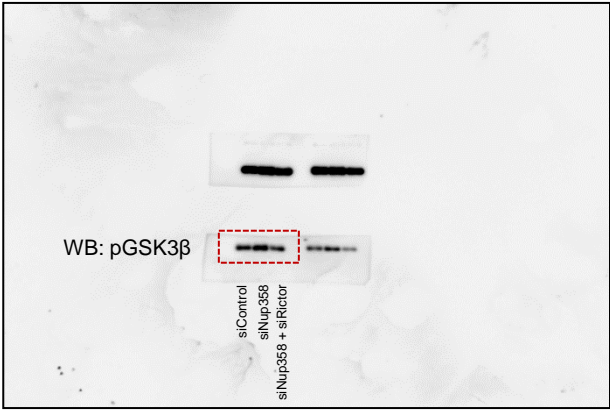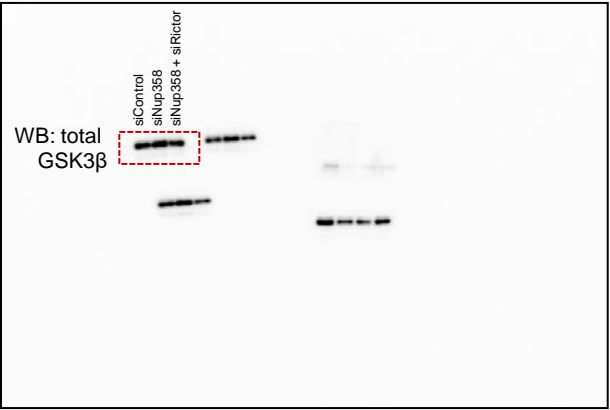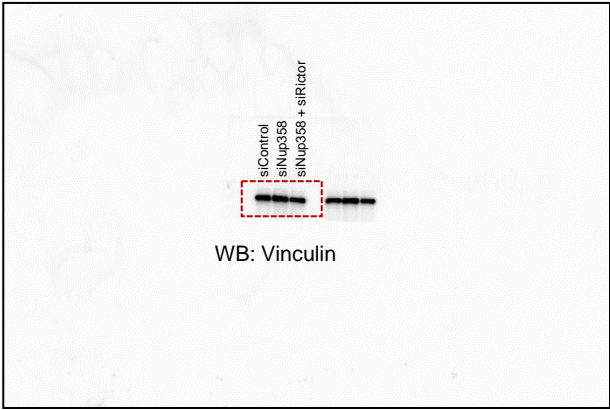

Supplement: Supplementary file 7 — Source data Fig. 5 [file 44319_2024_204_MOESM7_ESM.zip › Figure 5/5A/Figure 5A Western Blots (Top).pdf]

## Uncropped western blots related to Figure 5B (Bottom Left)

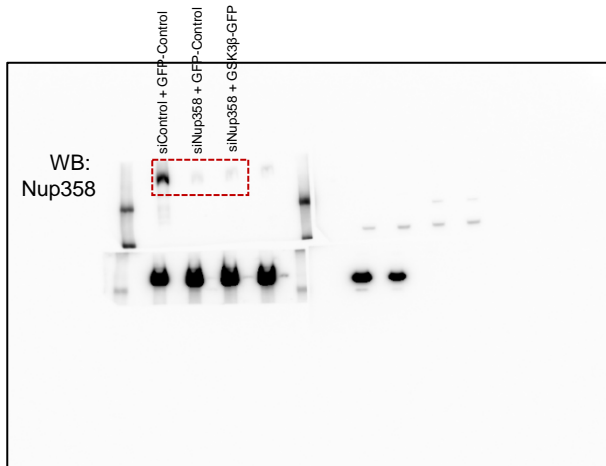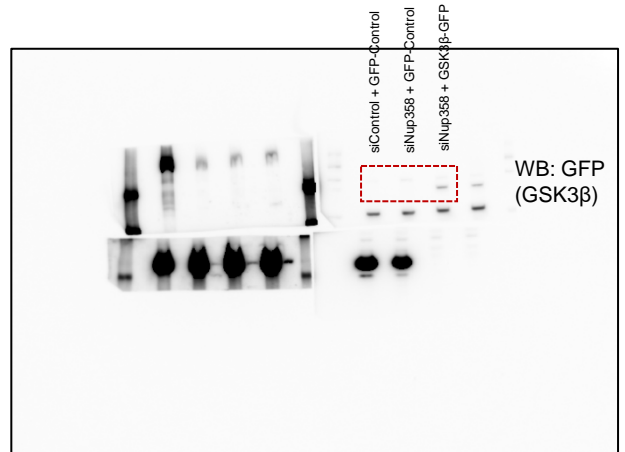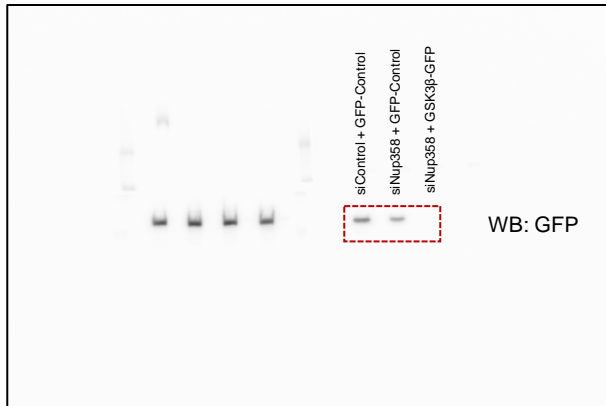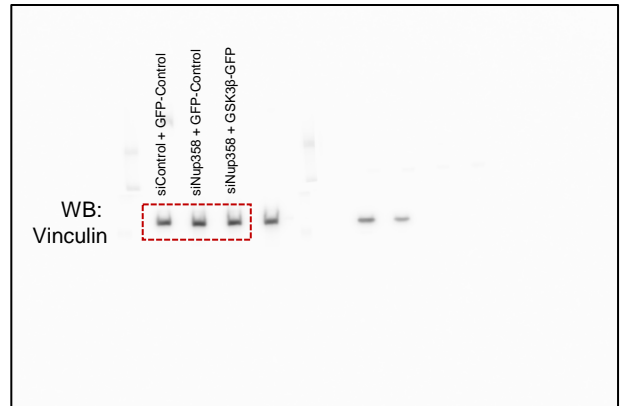

Supplement: Supplementary file 7 — Source data Fig. 5 [file 44319_2024_204_MOESM7_ESM.zip › Figure 5/5B/Figure 5B Western Blots (Bottom Left).pdf]

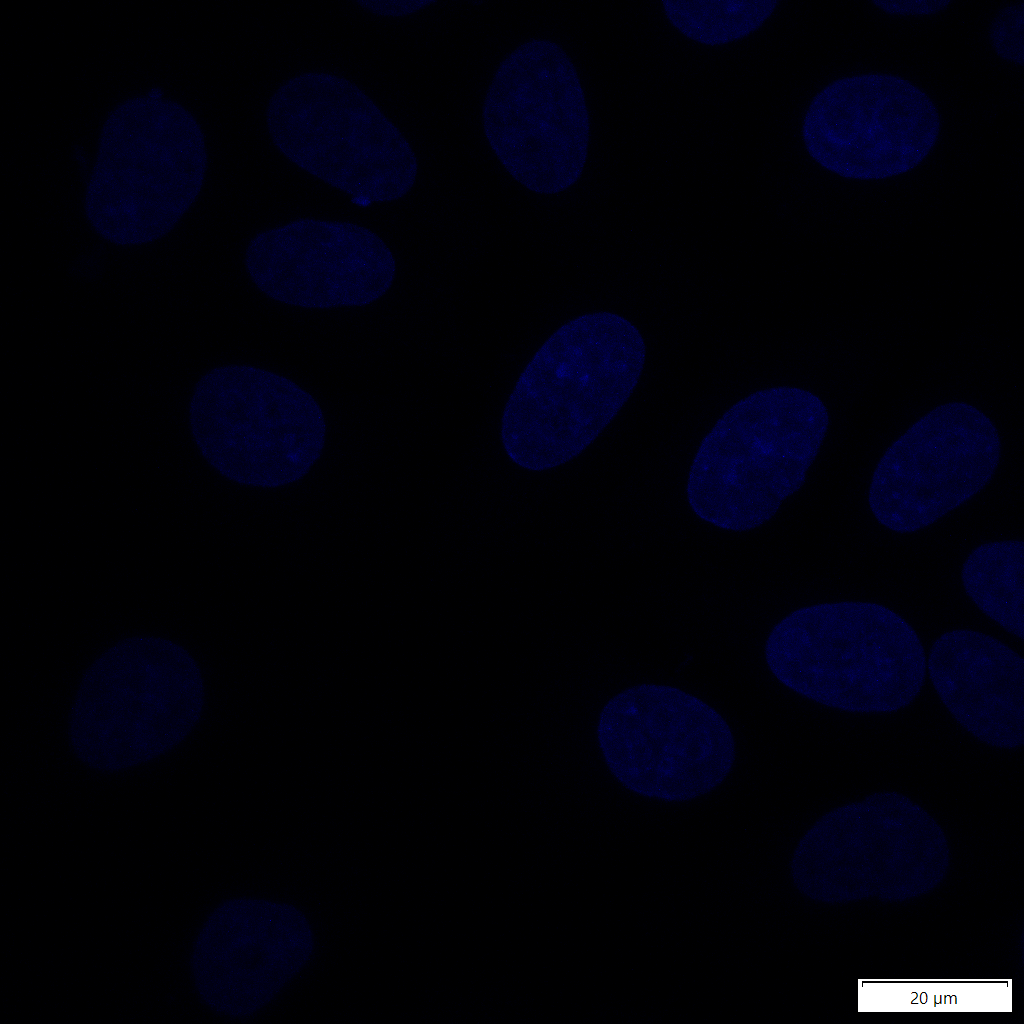

Supplement: Supplementary file 7 — Source data Fig. 5 [file 44319_2024_204_MOESM7_ESM.zip › Figure 5/5B/siControl + GFP-Control (IF images)/DAPI.tif]

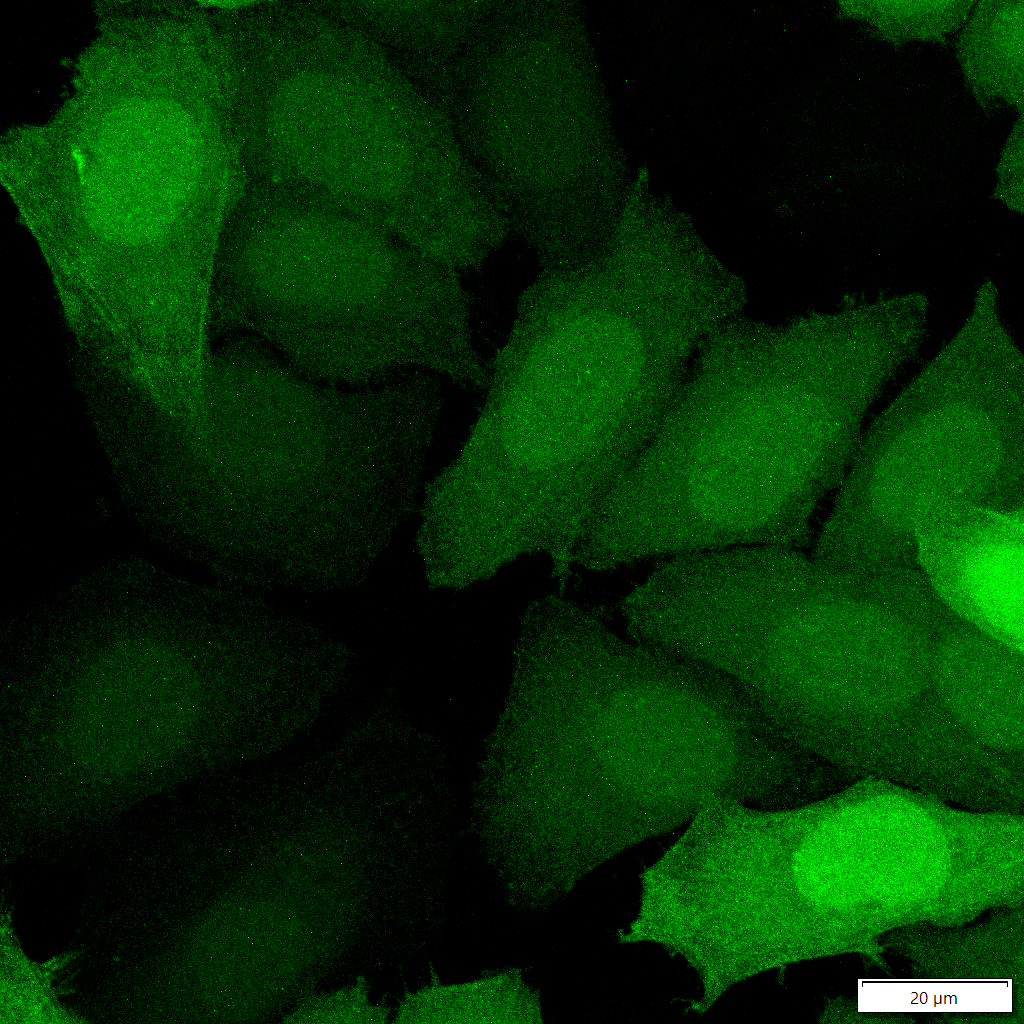

Supplement: Supplementary file 7 — Source data Fig. 5 [file 44319_2024_204_MOESM7_ESM.zip › Figure 5/5B/siControl + GFP-Control (IF images)/GFP-control.tif]

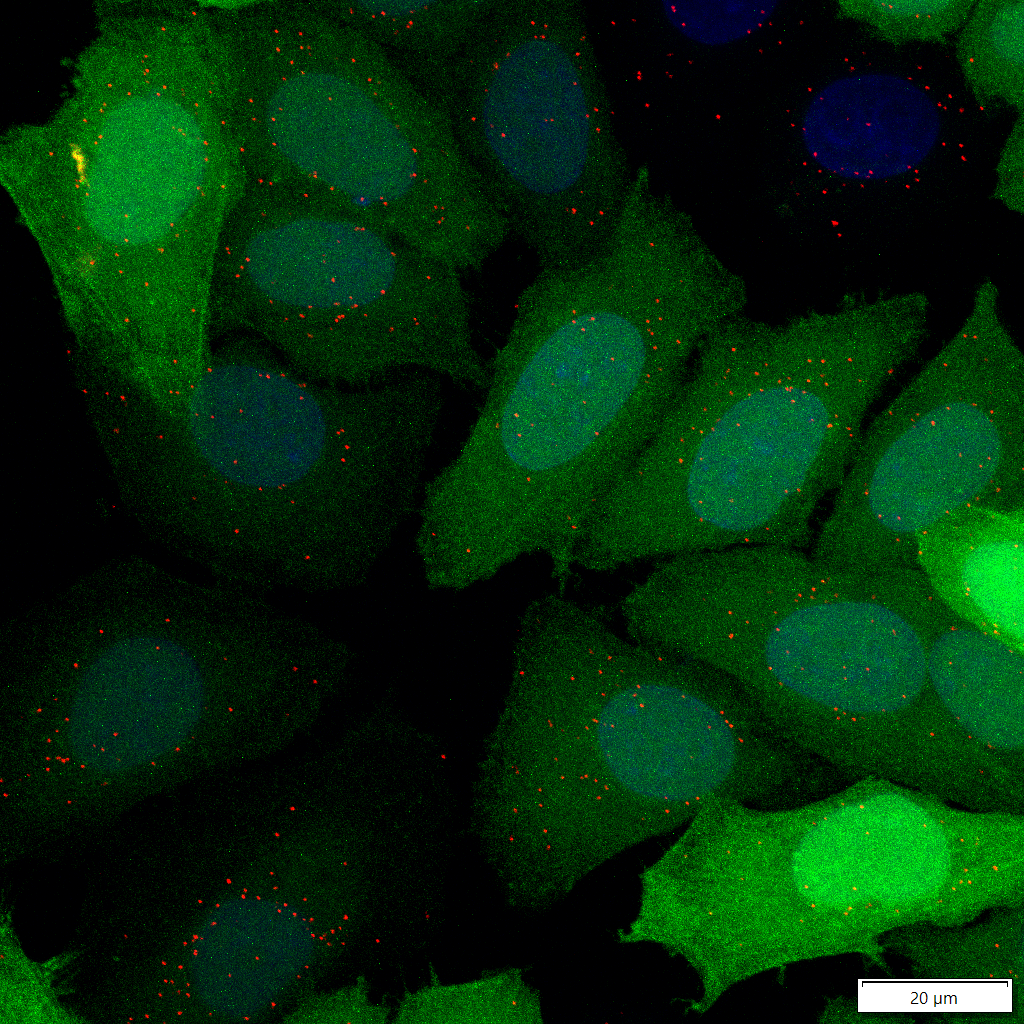

Supplement: Supplementary file 7 — Source data Fig. 5 [file 44319_2024_204_MOESM7_ESM.zip › Figure 5/5B/siControl + GFP-Control (IF images)/MERGE.tif]

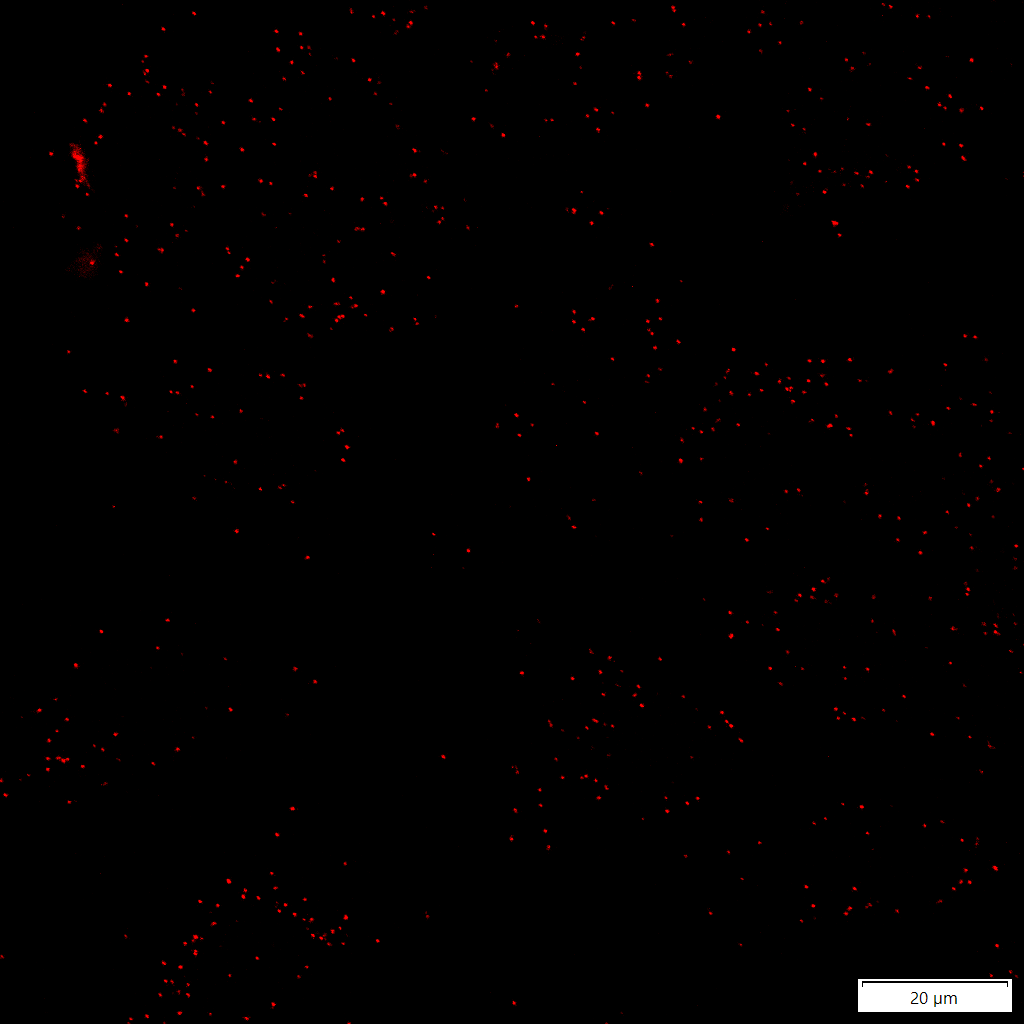

Supplement: Supplementary file 7 — Source data Fig. 5 [file 44319_2024_204_MOESM7_ESM.zip › Figure 5/5B/siControl + GFP-Control (IF images)/PLA (VAPB+PTPIP51).tif]

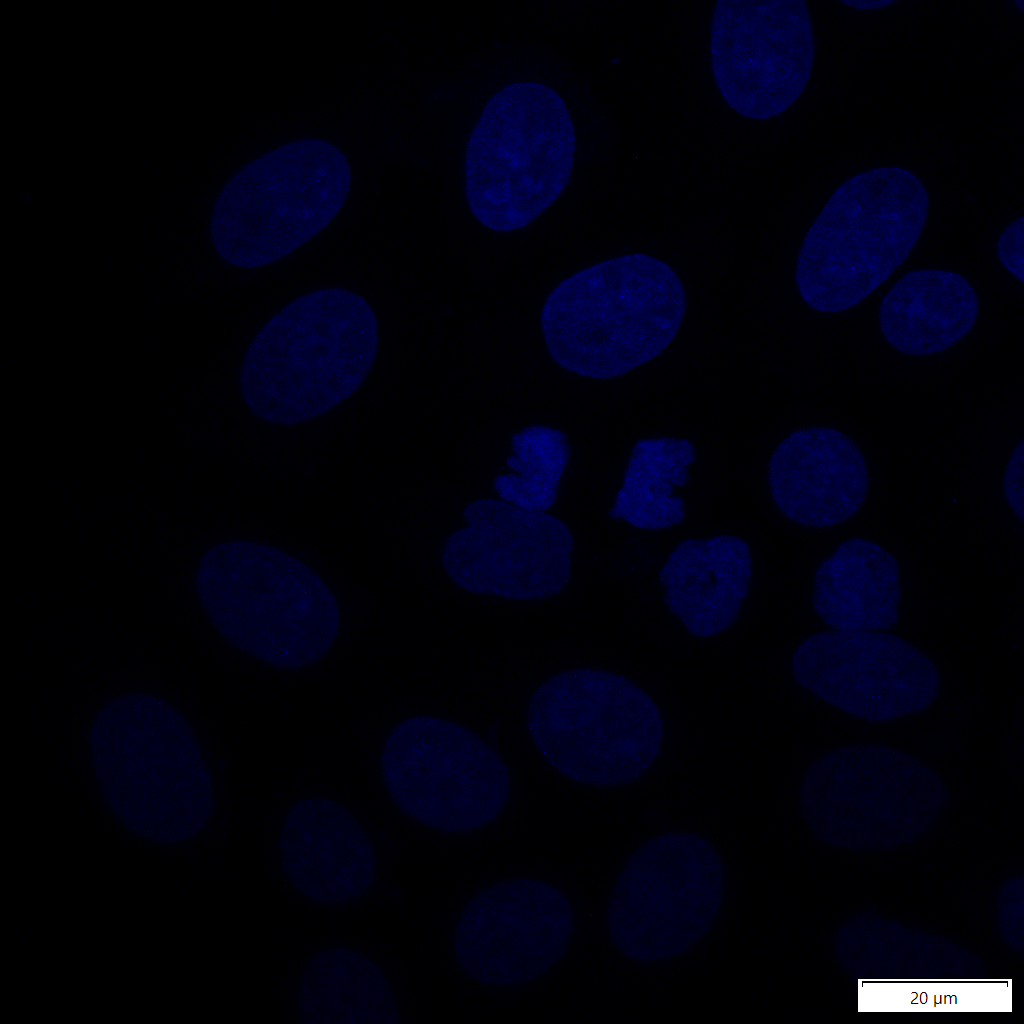

Supplement: Supplementary file 7 — Source data Fig. 5 [file 44319_2024_204_MOESM7_ESM.zip › Figure 5/5B/siNup358 + GFP-Control (IF images)/DAPI.tif]

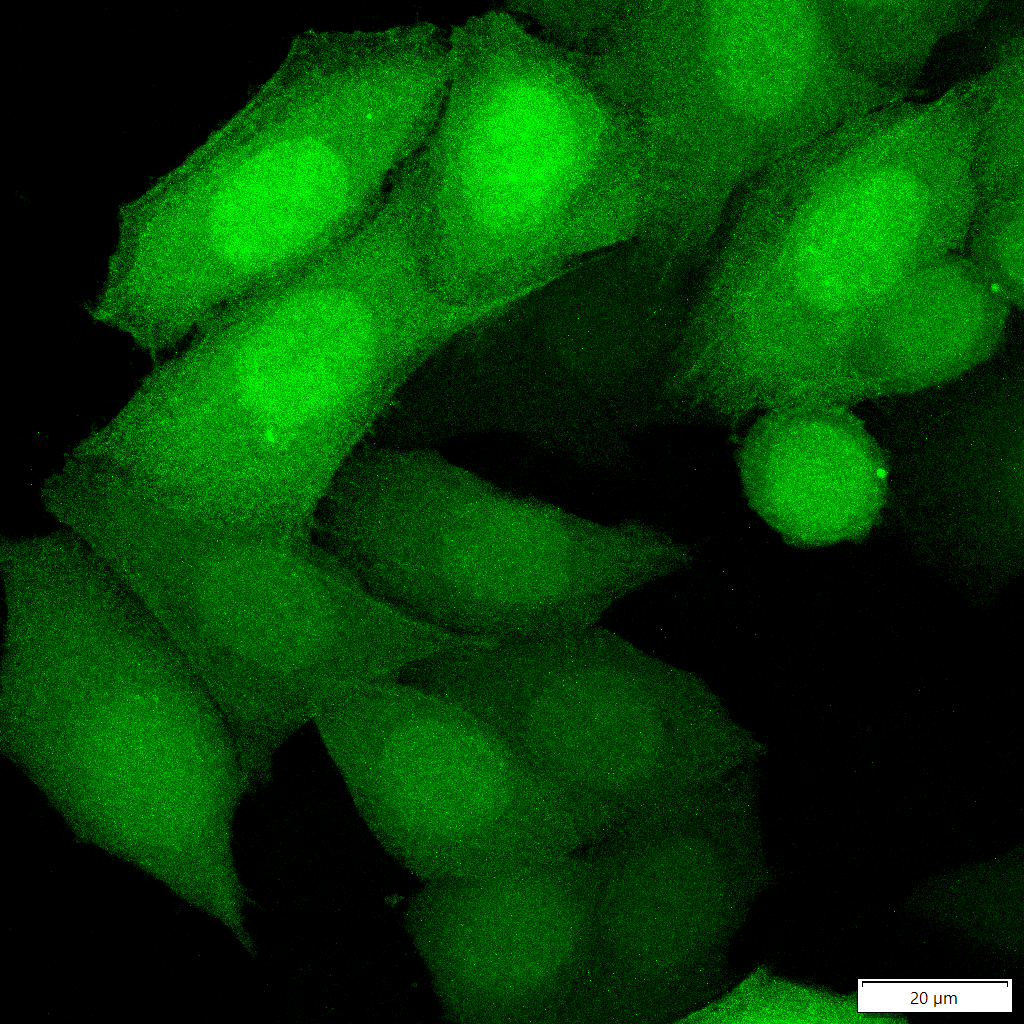

Supplement: Supplementary file 7 — Source data Fig. 5 [file 44319_2024_204_MOESM7_ESM.zip › Figure 5/5B/siNup358 + GFP-Control (IF images)/GFP-control.tif]

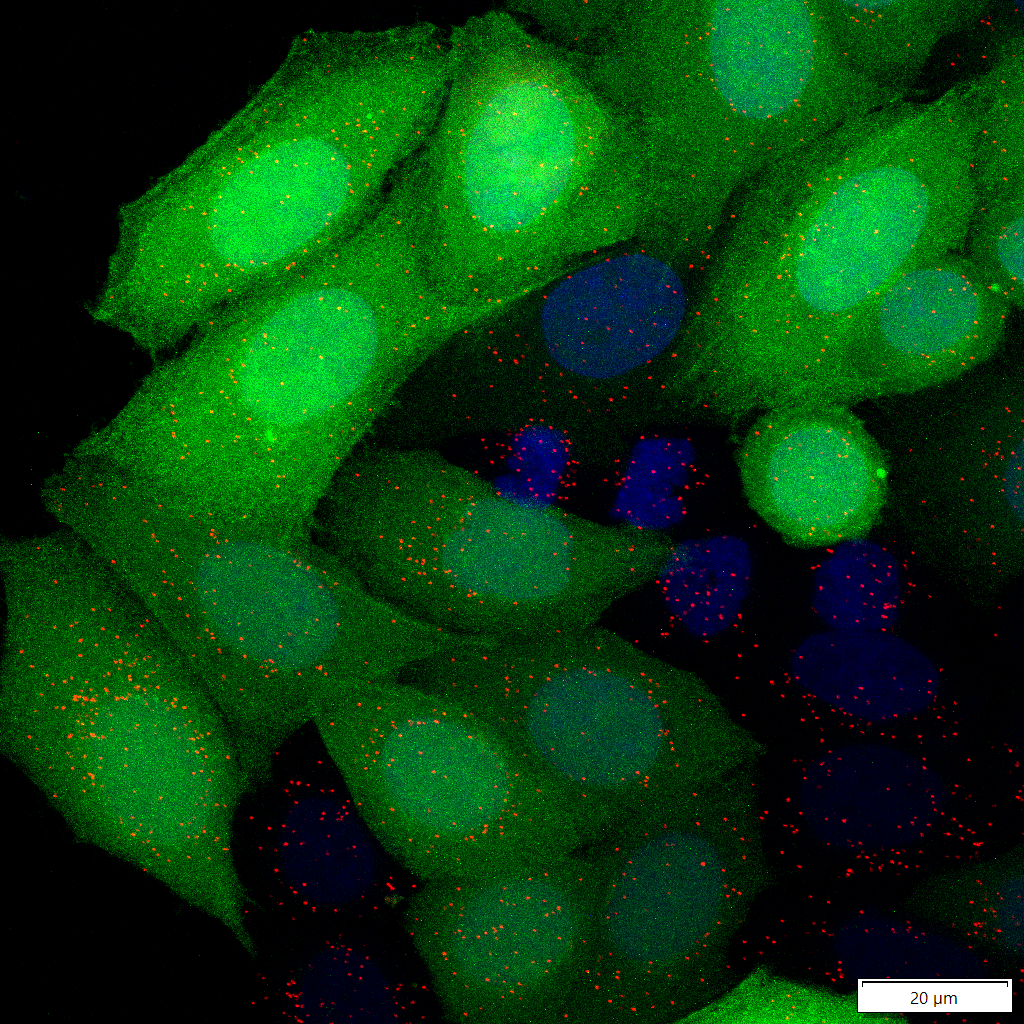

Supplement: Supplementary file 7 — Source data Fig. 5 [file 44319_2024_204_MOESM7_ESM.zip › Figure 5/5B/siNup358 + GFP-Control (IF images)/MERGE.tif]

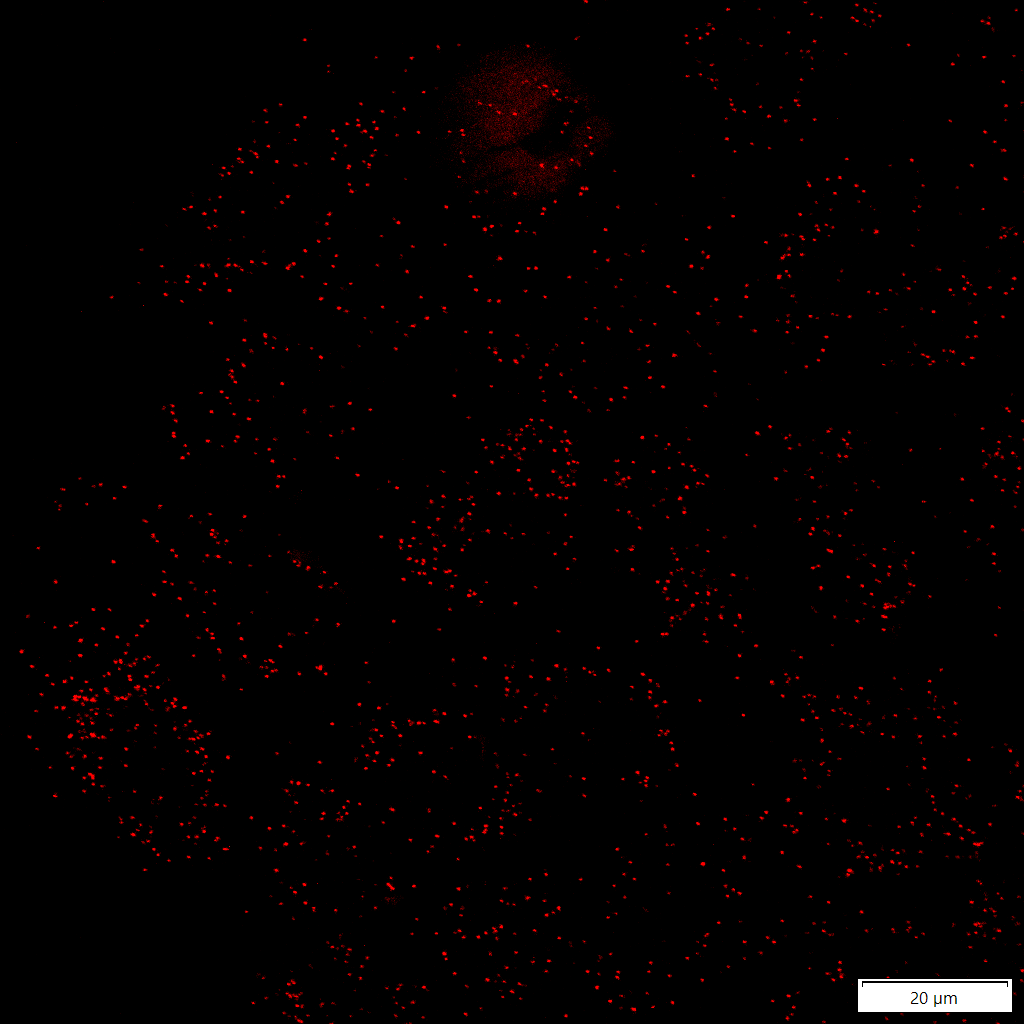

Supplement: Supplementary file 7 — Source data Fig. 5 [file 44319_2024_204_MOESM7_ESM.zip › Figure 5/5B/siNup358 + GFP-Control (IF images)/PLA (VAPB+PTPIP51).tif]

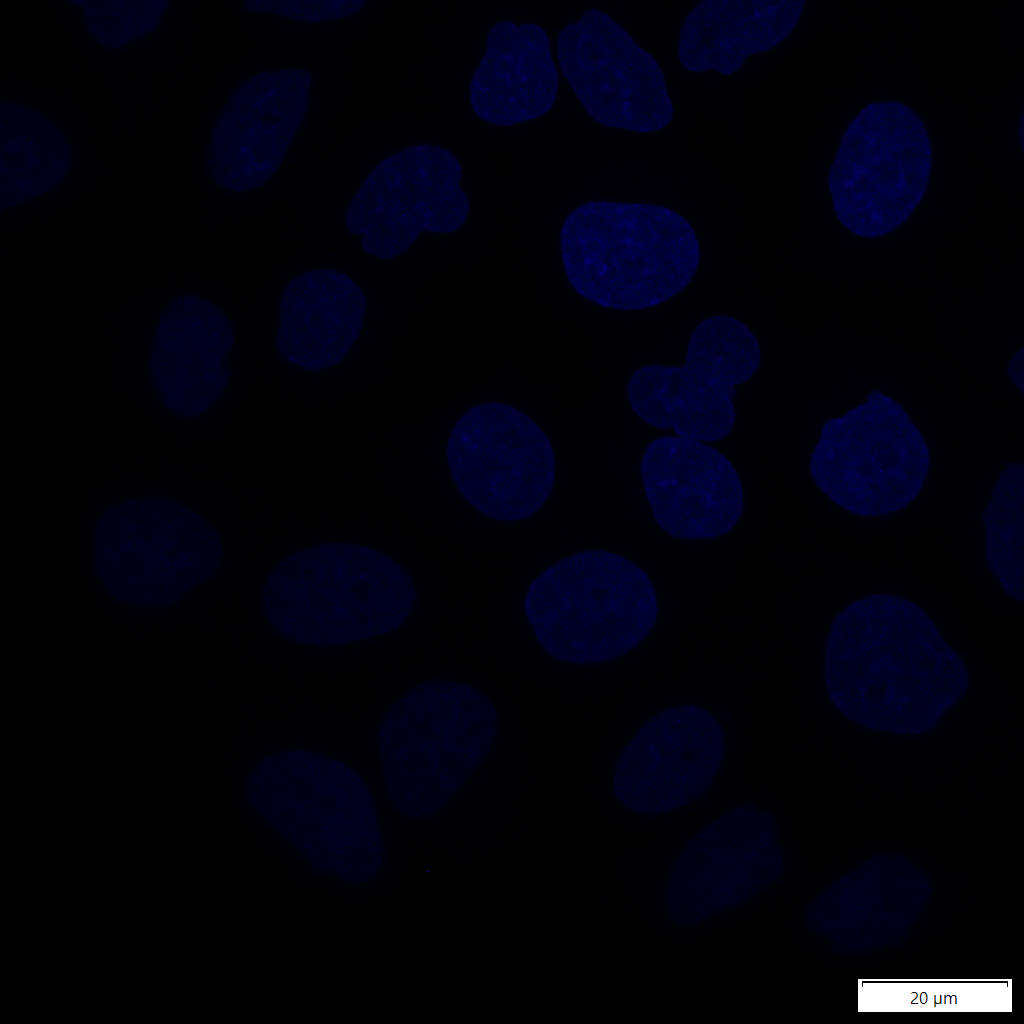

Supplement: Supplementary file 7 — Source data Fig. 5 [file 44319_2024_204_MOESM7_ESM.zip › Figure 5/5B/siNup358 + GSK3b-GFP (IF images)/DAPI.tif]

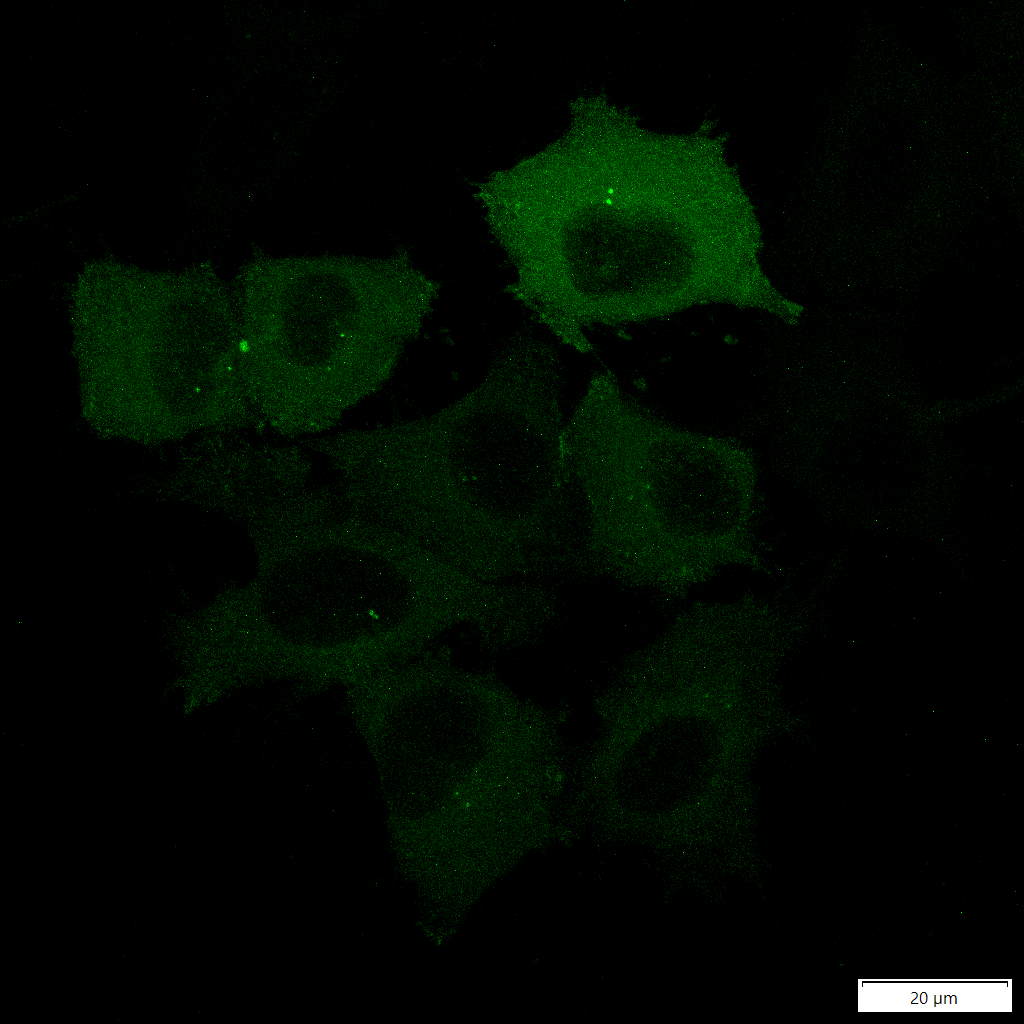

Supplement: Supplementary file 7 — Source data Fig. 5 [file 44319_2024_204_MOESM7_ESM.zip › Figure 5/5B/siNup358 + GSK3b-GFP (IF images)/GSK3b-GFP.tif]

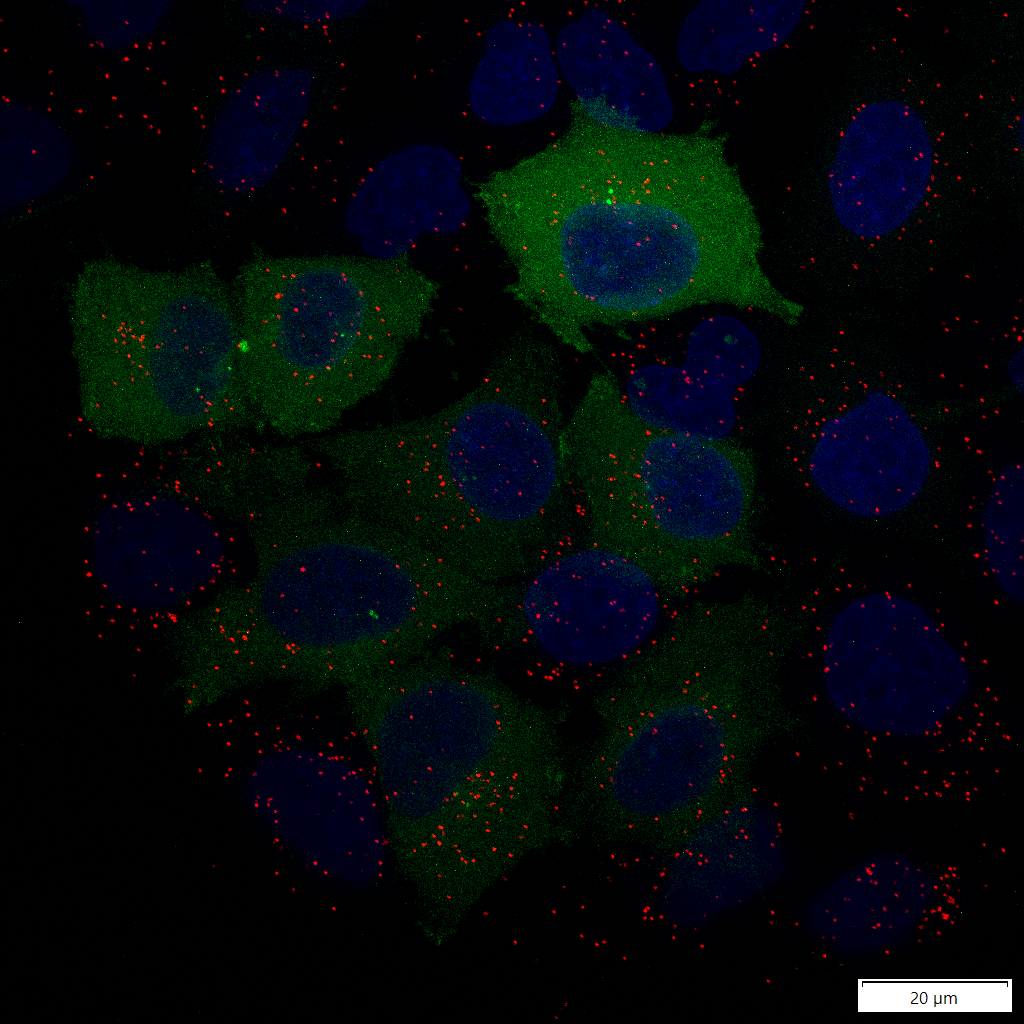

Supplement: Supplementary file 7 — Source data Fig. 5 [file 44319_2024_204_MOESM7_ESM.zip › Figure 5/5B/siNup358 + GSK3b-GFP (IF images)/MERGE.tif]

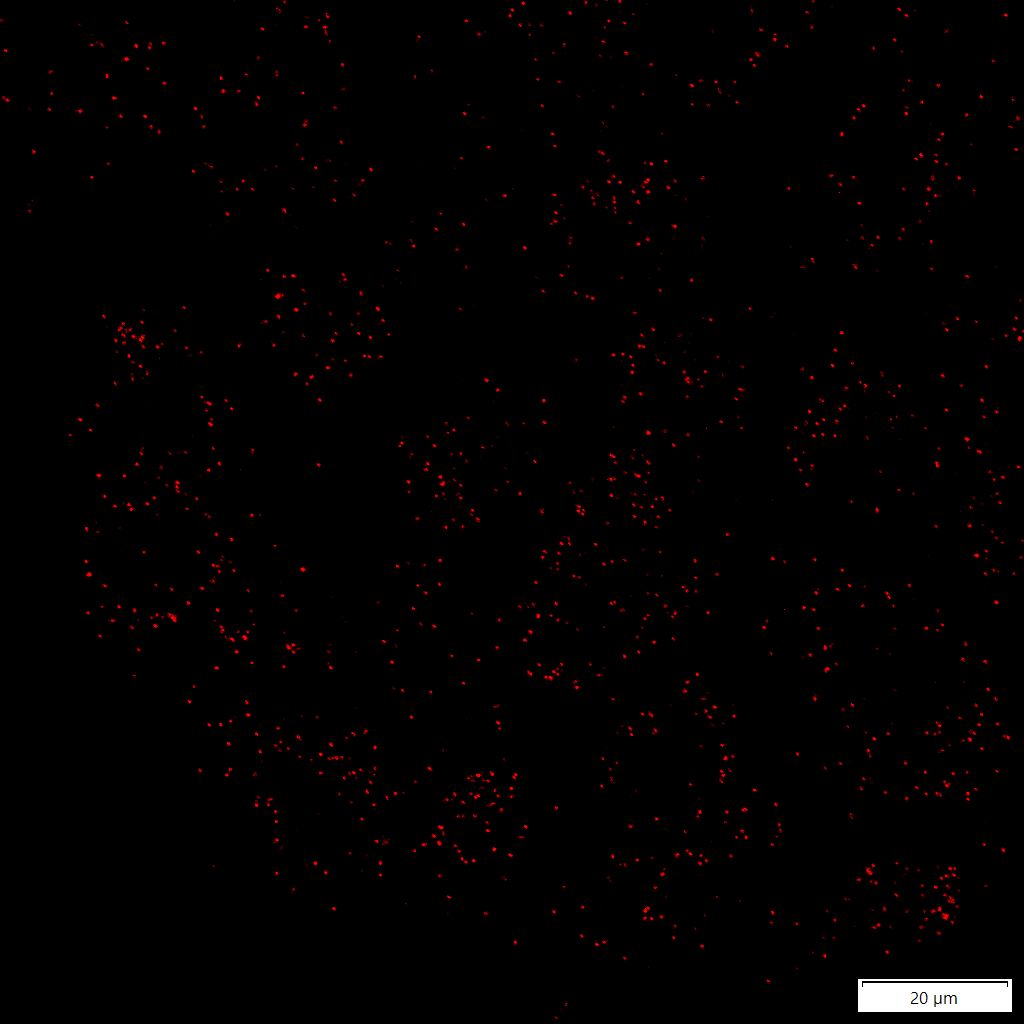

Supplement: Supplementary file 7 — Source data Fig. 5 [file 44319_2024_204_MOESM7_ESM.zip › Figure 5/5B/siNup358 + GSK3b-GFP (IF images)/PLA (VAPB+PTPIP51).tif]

Uncropped western blots related to Figure 5C (Left)

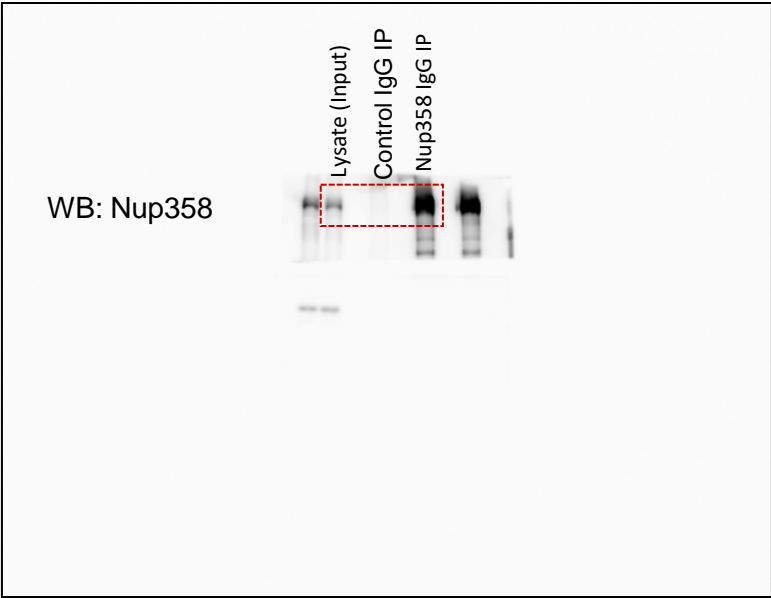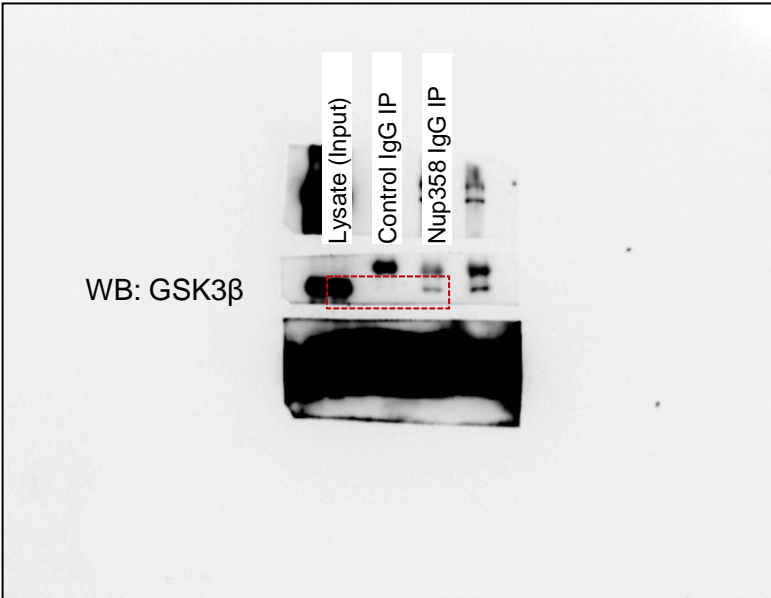

Supplement: Supplementary file 7 — Source data Fig. 5 [file 44319_2024_204_MOESM7_ESM.zip › Figure 5/5C/Figure 5C_Western Blots (Left).pdf]

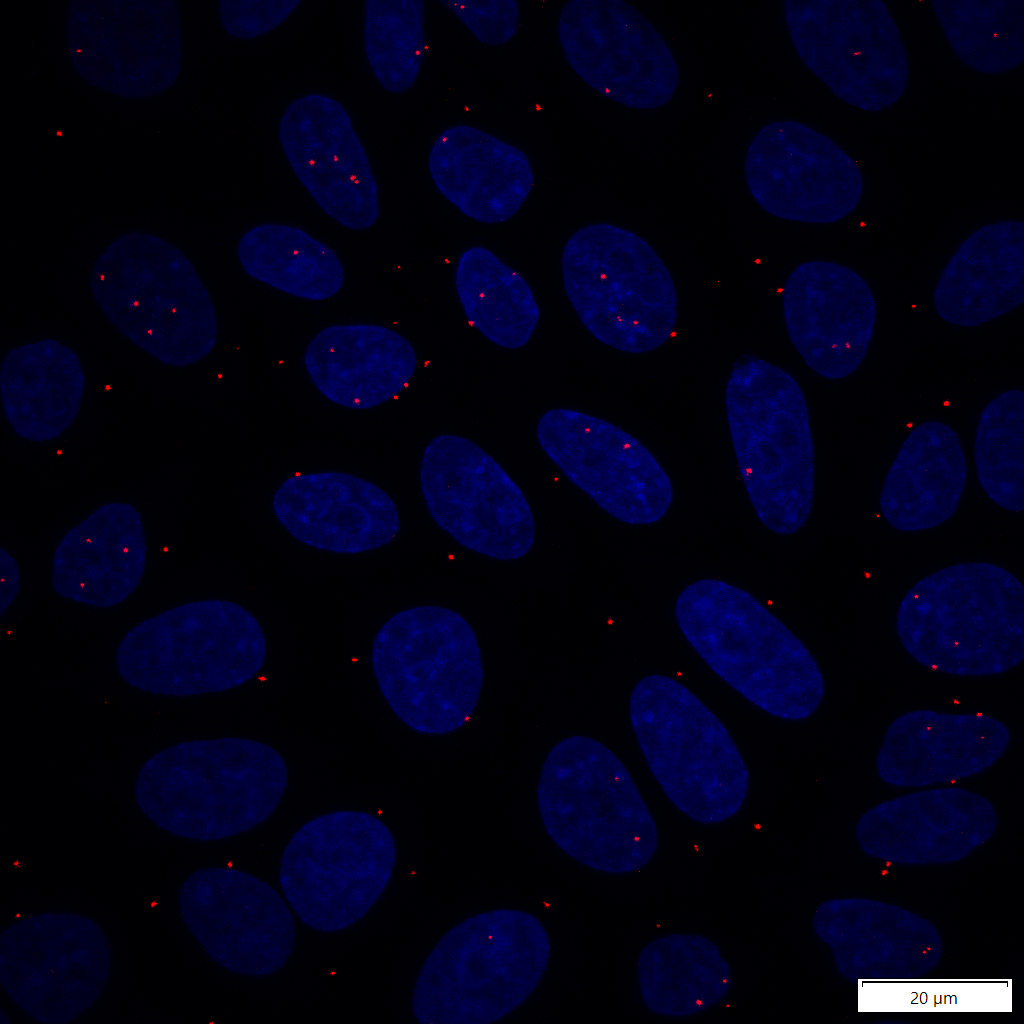

Supplement: Supplementary file 7 — Source data Fig. 5 [file 44319_2024_204_MOESM7_ESM.zip › Figure 5/5C/GSK3b control PLA image.tif]

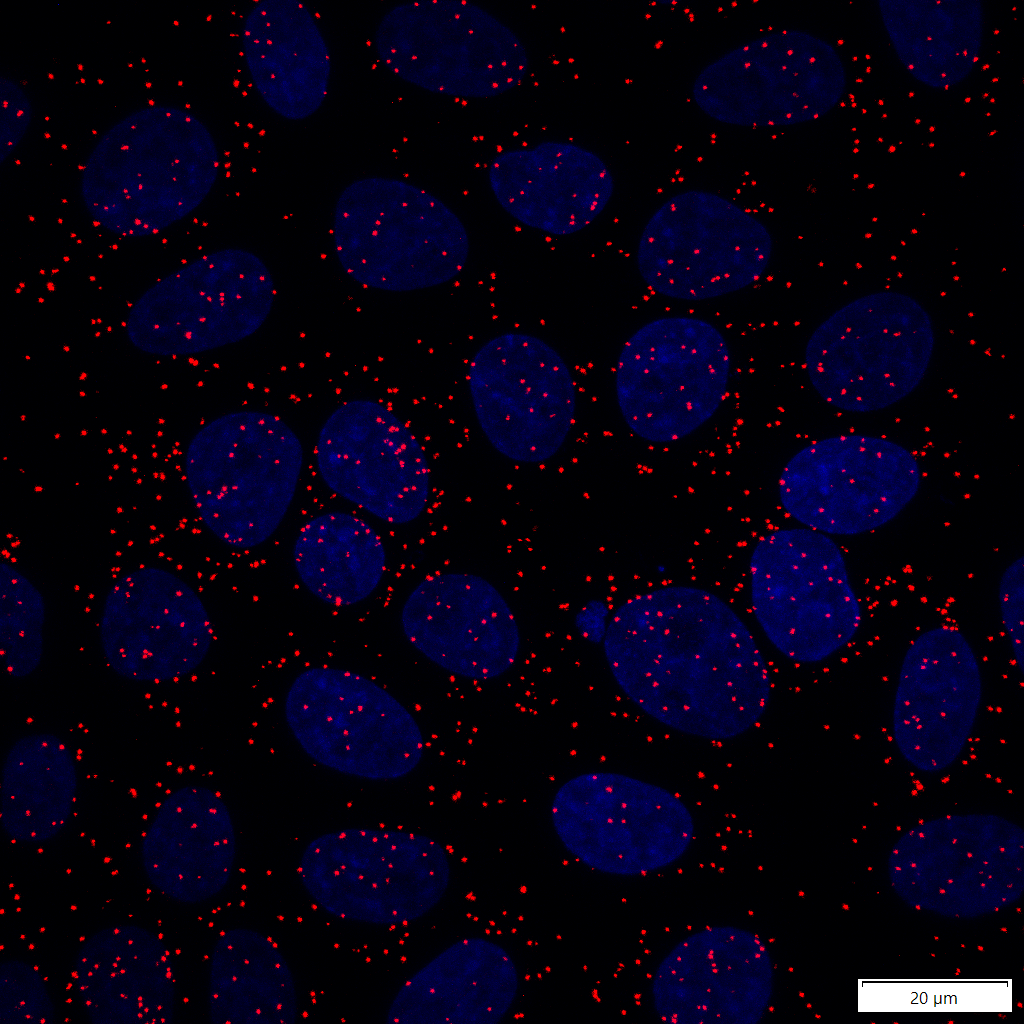

Supplement: Supplementary file 7 — Source data Fig. 5 [file 44319_2024_204_MOESM7_ESM.zip › Figure 5/5C/GSK3b+RanGAP1 PLA image.tif]

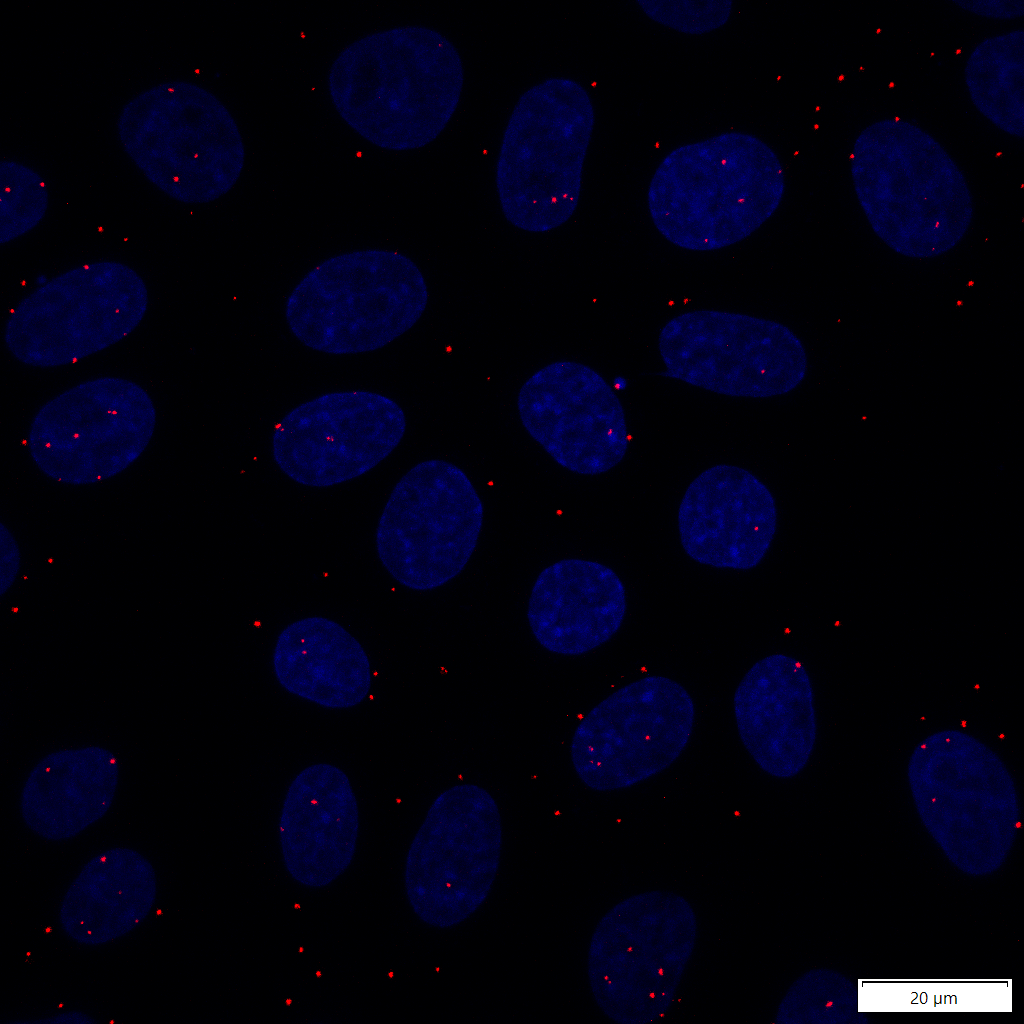

Supplement: Supplementary file 7 — Source data Fig. 5 [file 44319_2024_204_MOESM7_ESM.zip › Figure 5/5C/RanGAP1 control PLA image.tif]

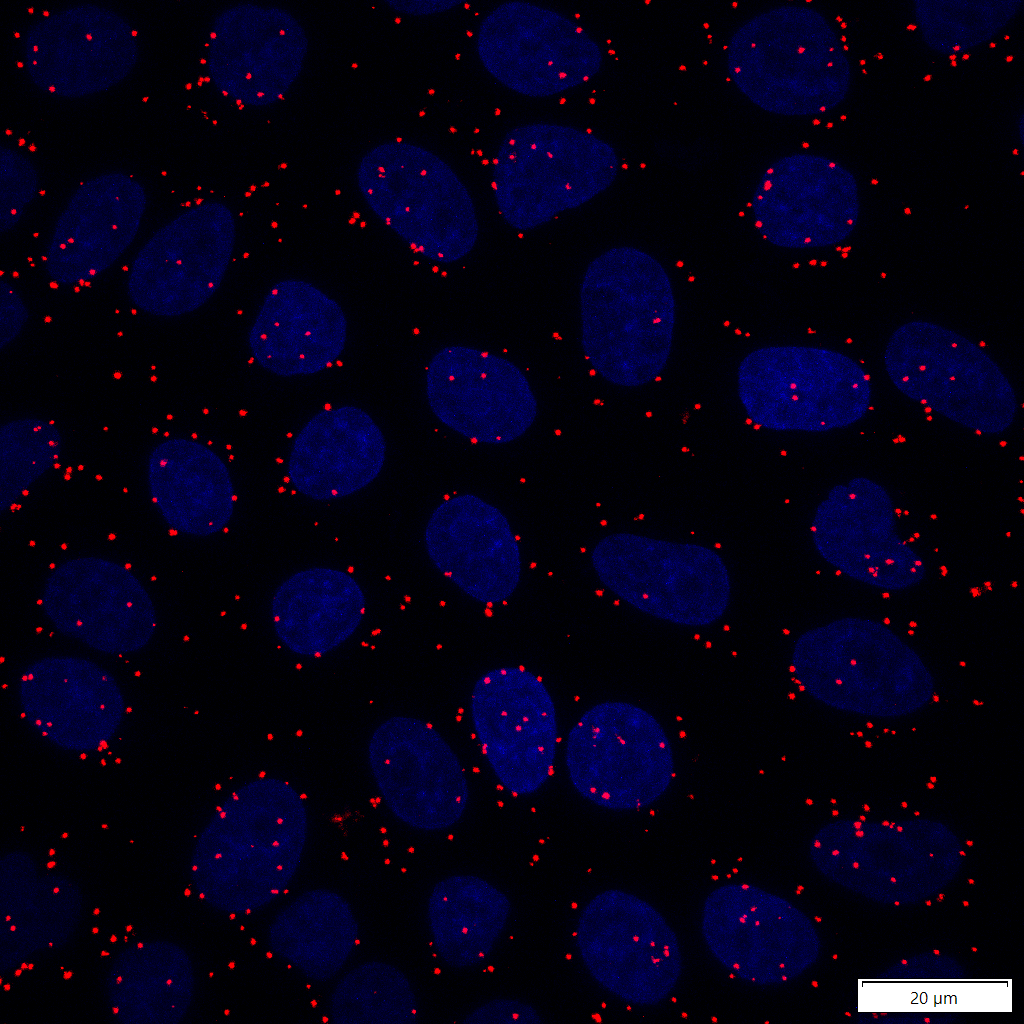

Supplement: Supplementary file 7 — Source data Fig. 5 [file 44319_2024_204_MOESM7_ESM.zip › Figure 5/5D/siControl - Insulin (VAPB+PTPIP51 PLA) image.tif]

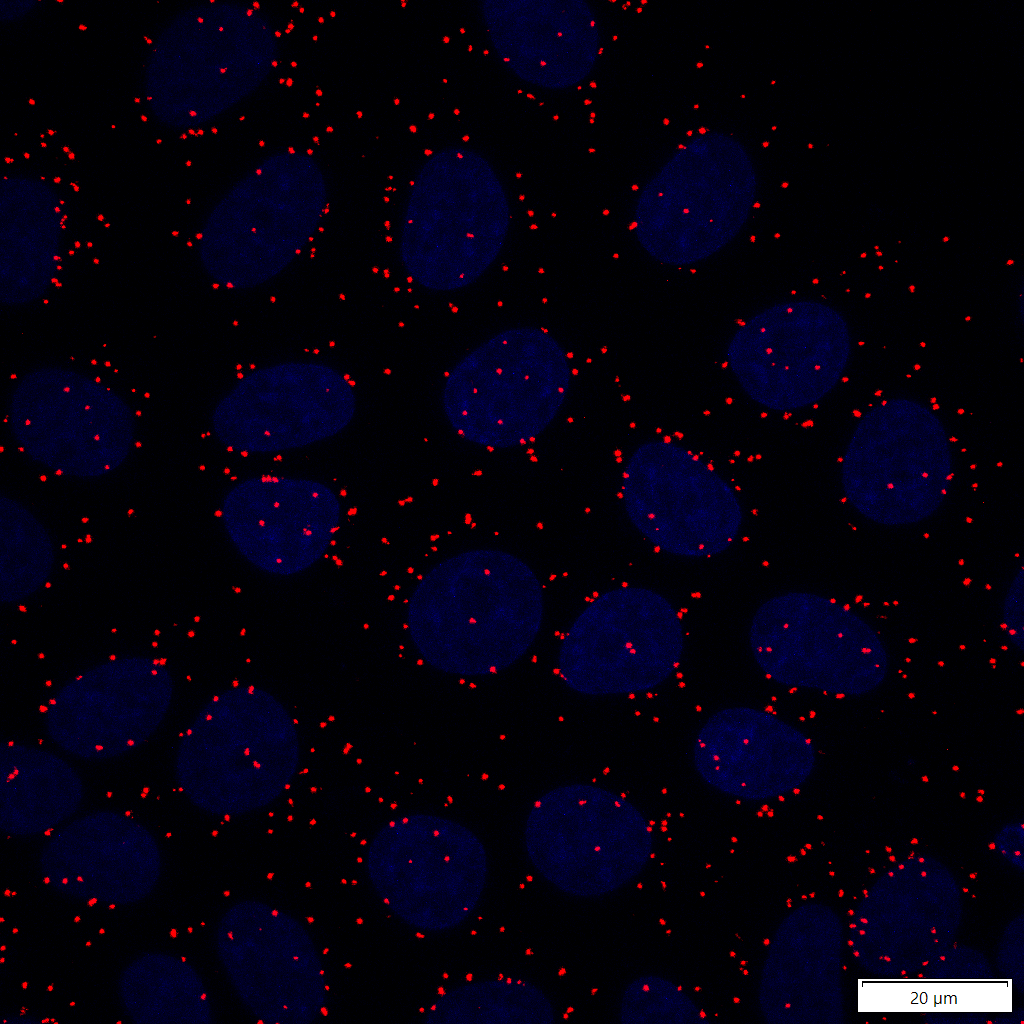

Supplement: Supplementary file 7 — Source data Fig. 5 [file 44319_2024_204_MOESM7_ESM.zip › Figure 5/5D/siControl + Insulin (VAPB+PTPIP51 PLA) image.tif]

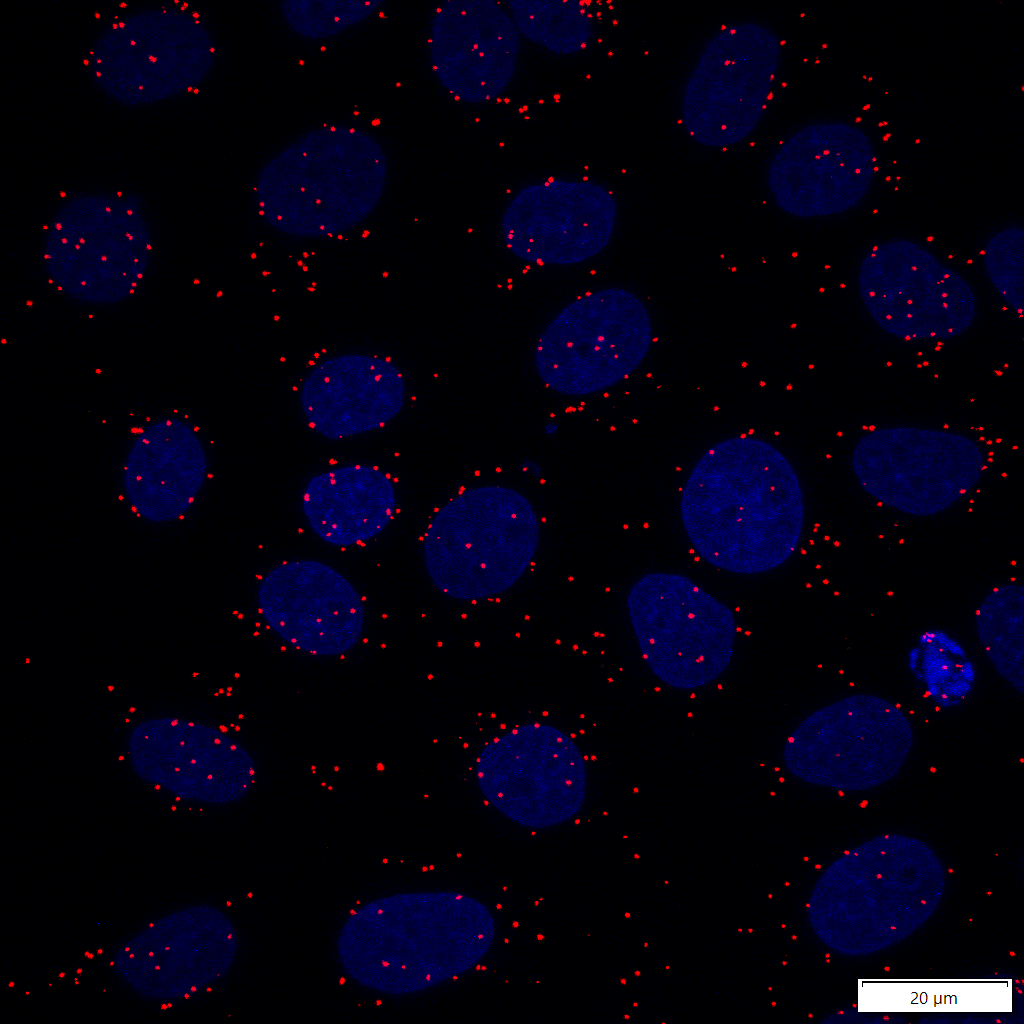

Supplement: Supplementary file 7 — Source data Fig. 5 [file 44319_2024_204_MOESM7_ESM.zip › Figure 5/5D/siNup358 - Insulin (VAPB+PTPIP51 PLA) image.tif]

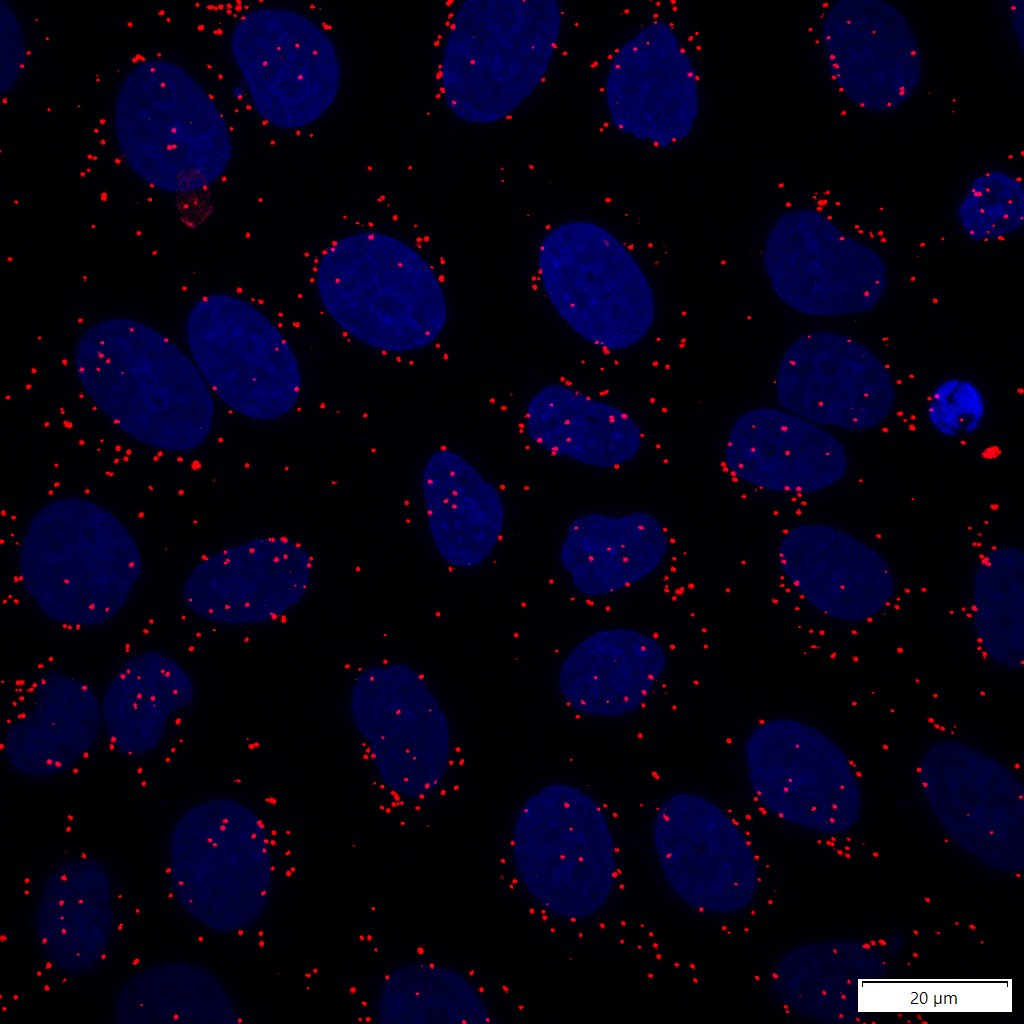

Supplement: Supplementary file 7 — Source data Fig. 5 [file 44319_2024_204_MOESM7_ESM.zip › Figure 5/5D/siNup358 + Insulin (VAPB+PTPIP51 PLA) image.tif]

Uncropped western blots related to Figure 6A (Bottom)

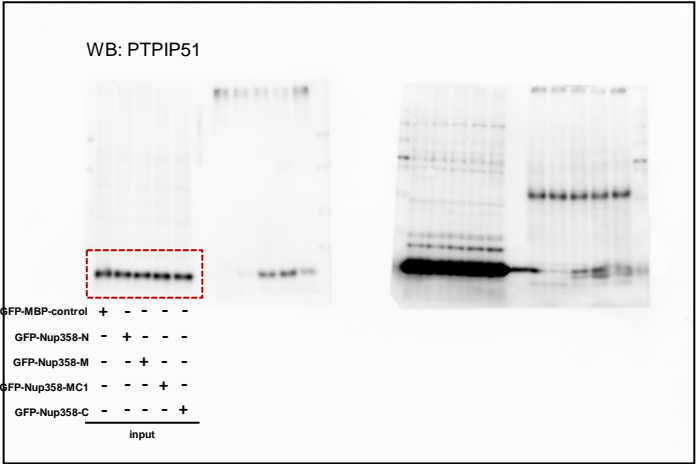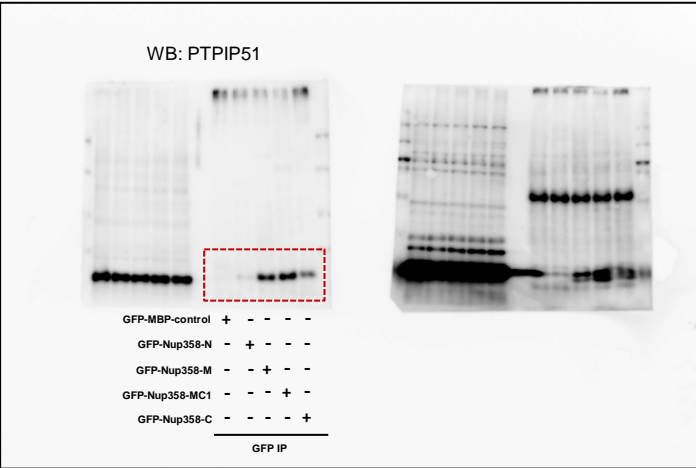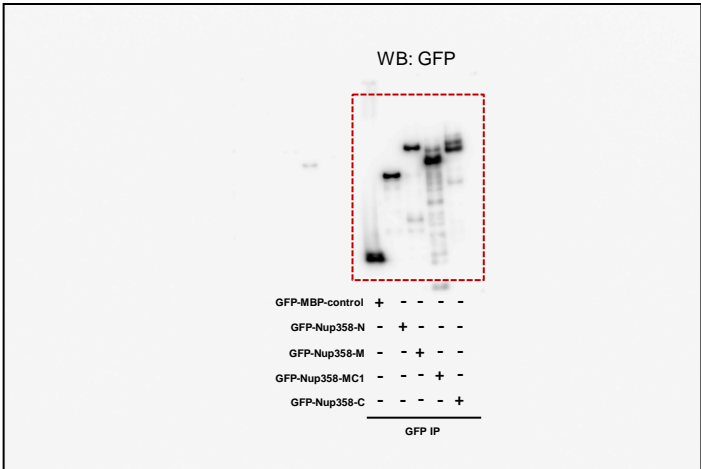

Supplement: Supplementary file 8 — Source data Fig. 6 [file 44319_2024_204_MOESM8_ESM.zip › Figure 6/6A/Fig 6A Western Blots (Bottom).pdf]

## Uncropped western blots related to Figure 6B

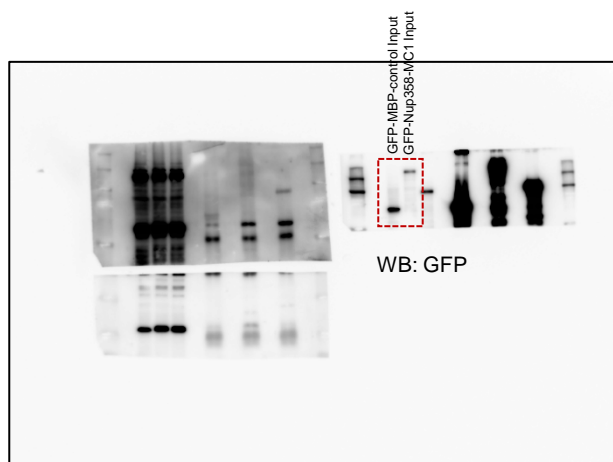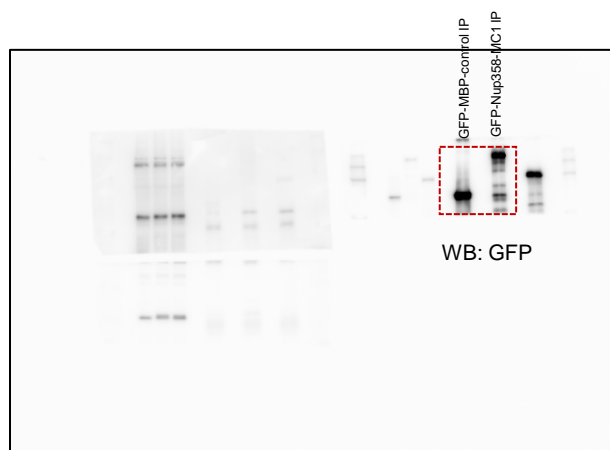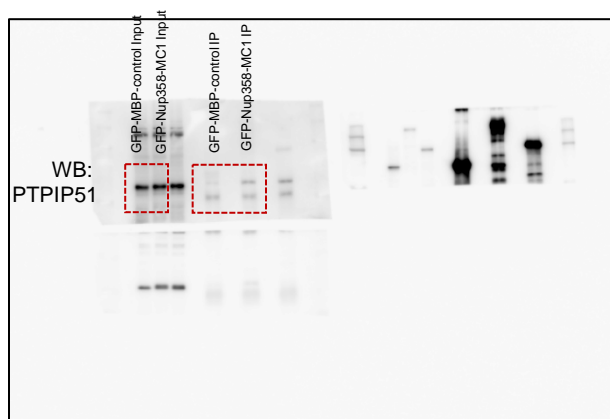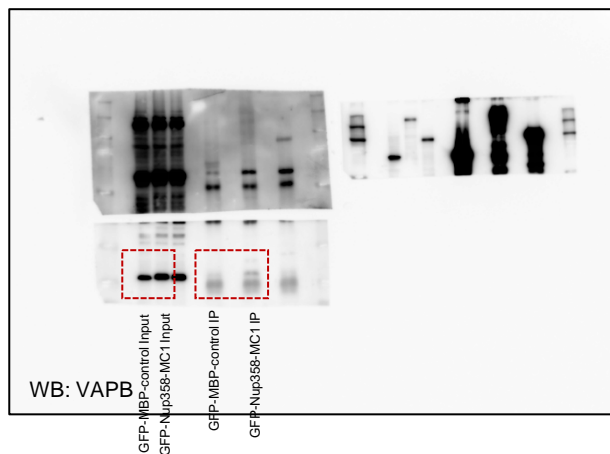

Supplement: Supplementary file 8 — Source data Fig. 6 [file 44319_2024_204_MOESM8_ESM.zip › Figure 6/6B/Figure 6B Western Blots.pdf]

Uncropped western blots related to Figure 5C (Left)

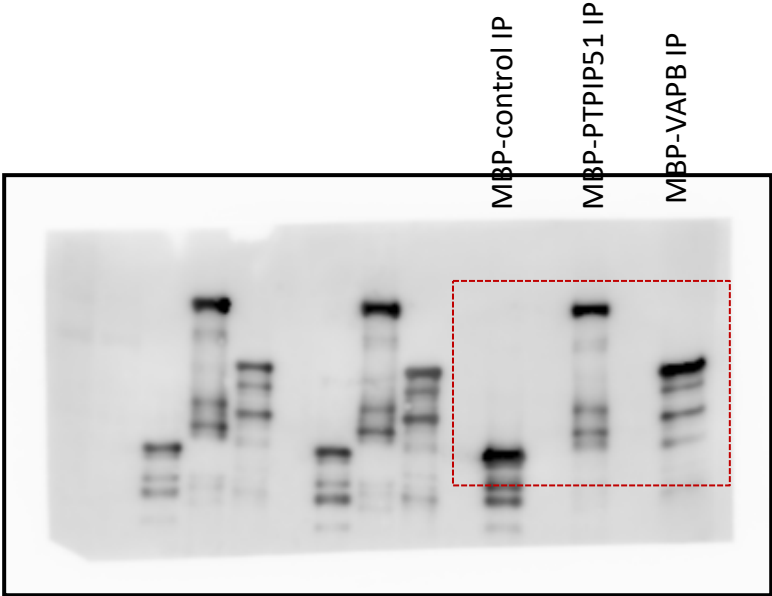

WB: MBP

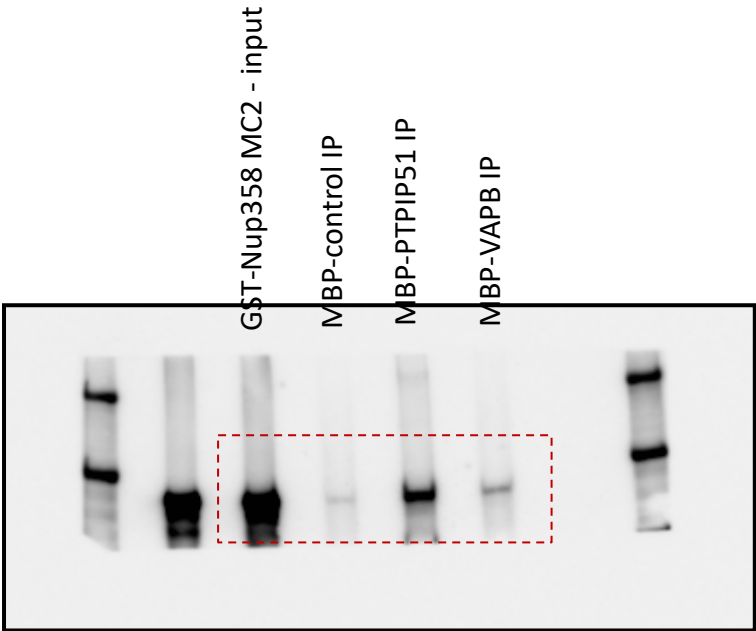

WB: Nup358-IR antibody

Supplement: Supplementary file 8 — Source data Fig. 6 [file 44319_2024_204_MOESM8_ESM.zip › Figure 6/6C/Figure 6C Western Blots (Left).pdf]

# Uncropped western blots related to Figure 6D (Left)

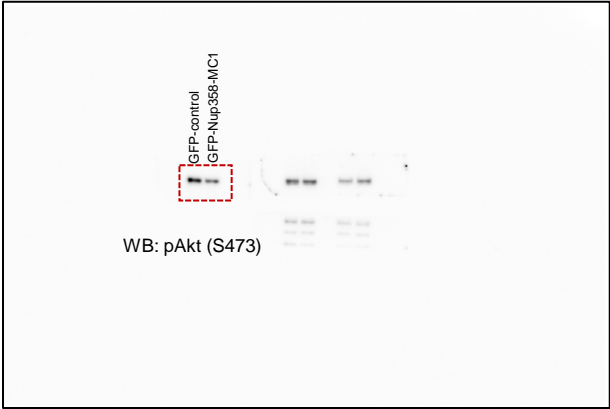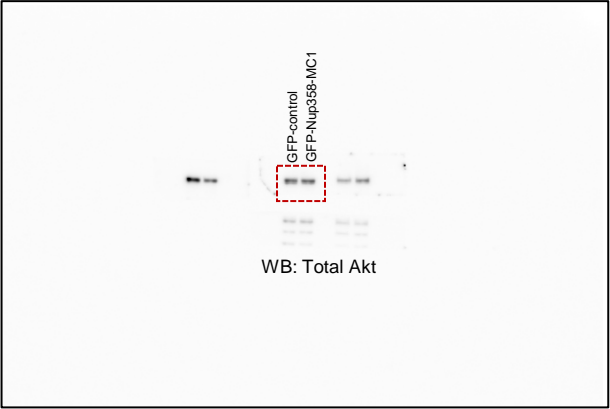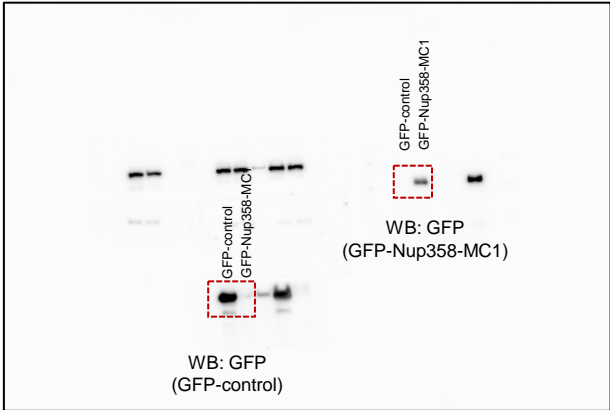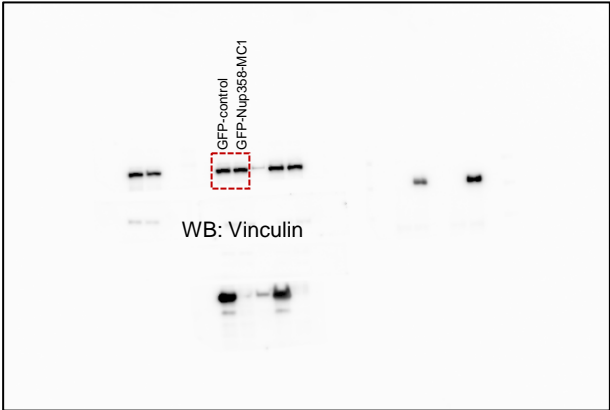

Supplement: Supplementary file 8 — Source data Fig. 6 [file 44319_2024_204_MOESM8_ESM.zip › Figure 6/6D/Figure 6D Western Blots (Left).pdf]

Uncropped western blots related to Figure 6E (Left)

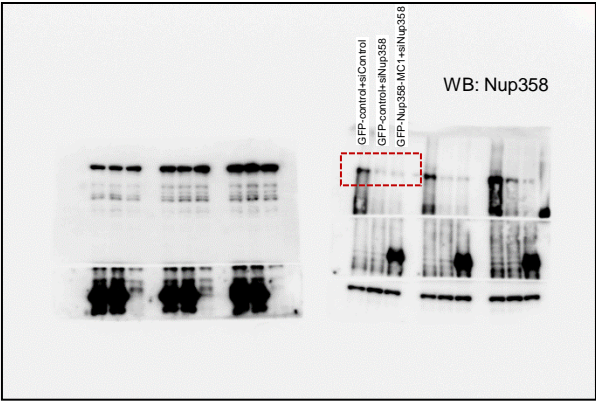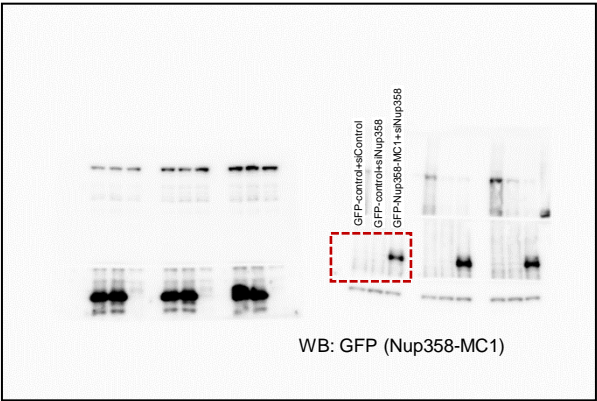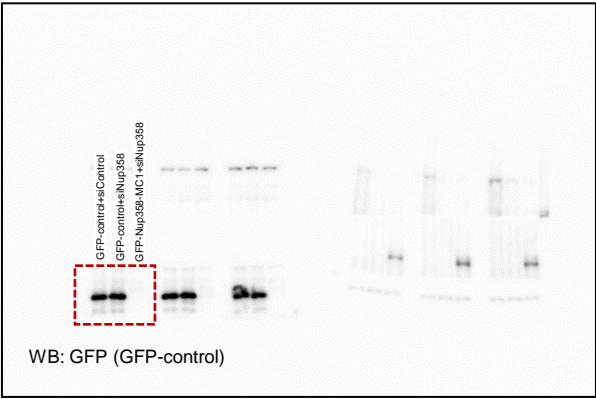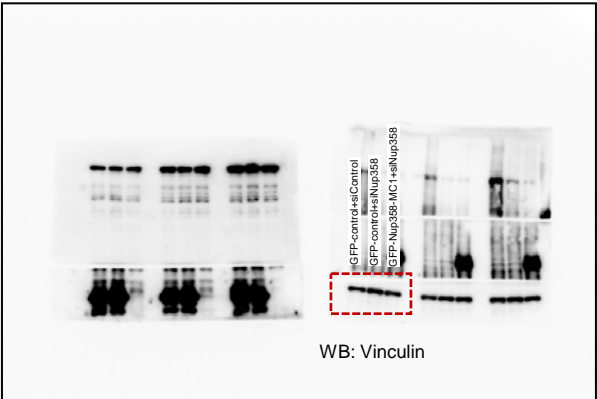

Supplement: Supplementary file 8 — Source data Fig. 6 [file 44319_2024_204_MOESM8_ESM.zip › Figure 6/6E/Figure 6E Western Blots (Left).pdf]

## Uncropped western blots related to Figure 6E (Right Top)

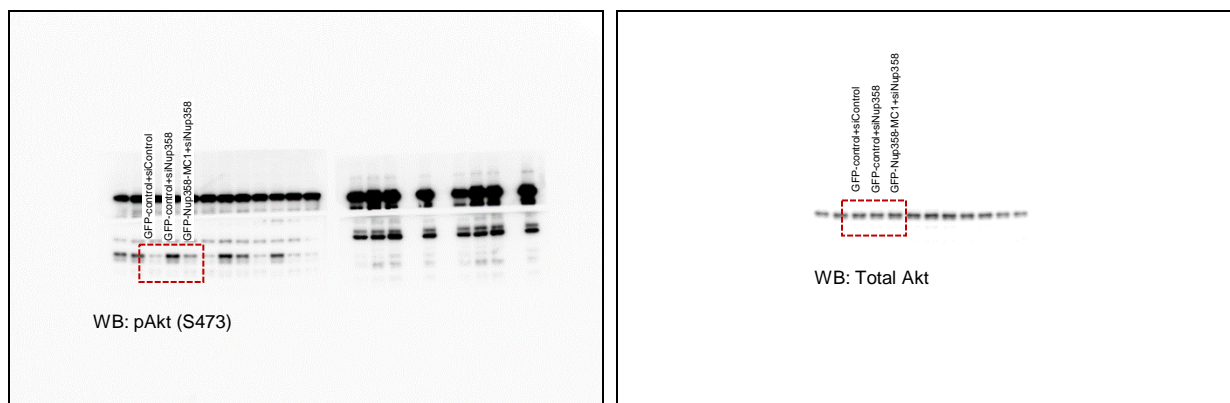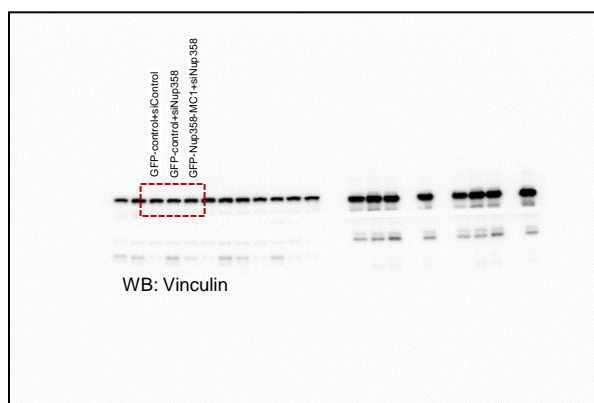

Supplement: Supplementary file 8 — Source data Fig. 6 [file 44319_2024_204_MOESM8_ESM.zip › Figure 6/6E/Figure 6E Western Blots (Right Top).pdf]

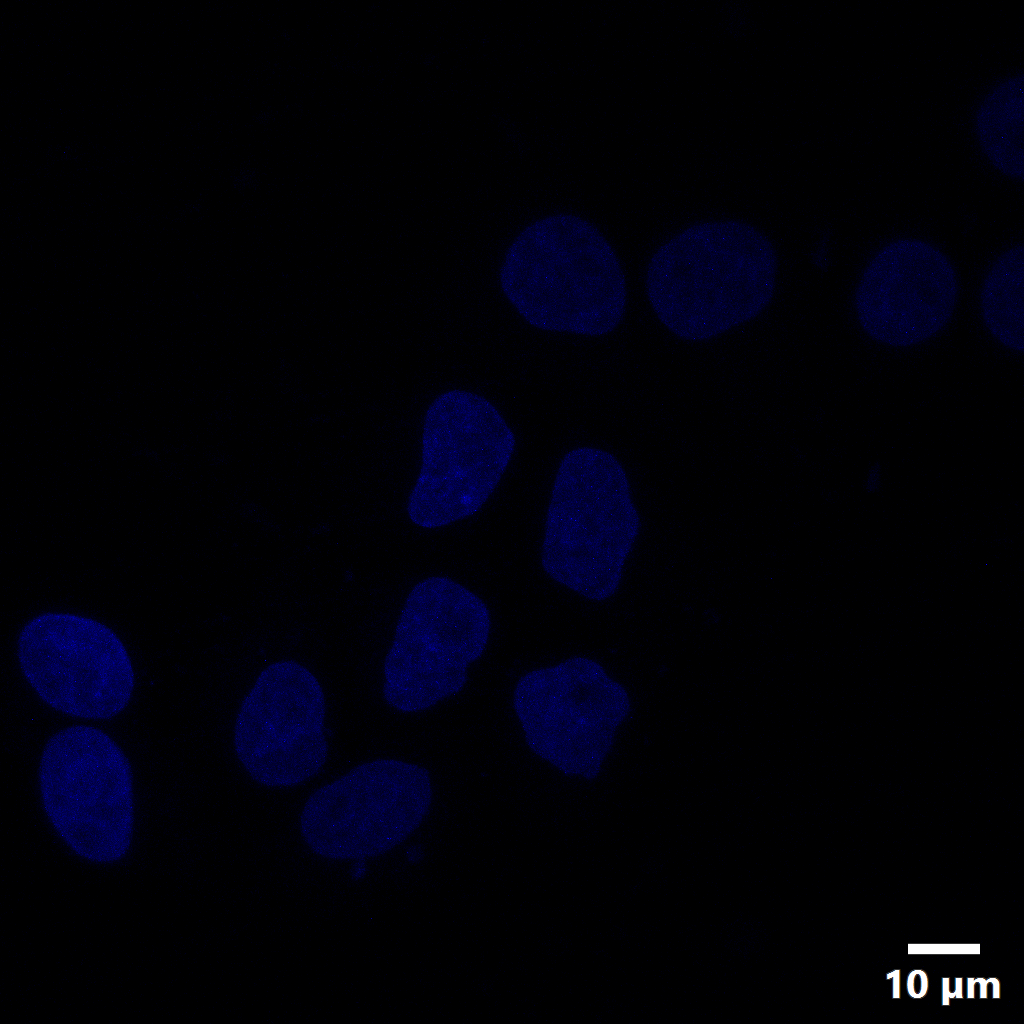

Supplement: Supplementary file 8 — Source data Fig. 6 [file 44319_2024_204_MOESM8_ESM.zip › Figure 6/6F/GFP-control (IF images)/DAPI.tif]

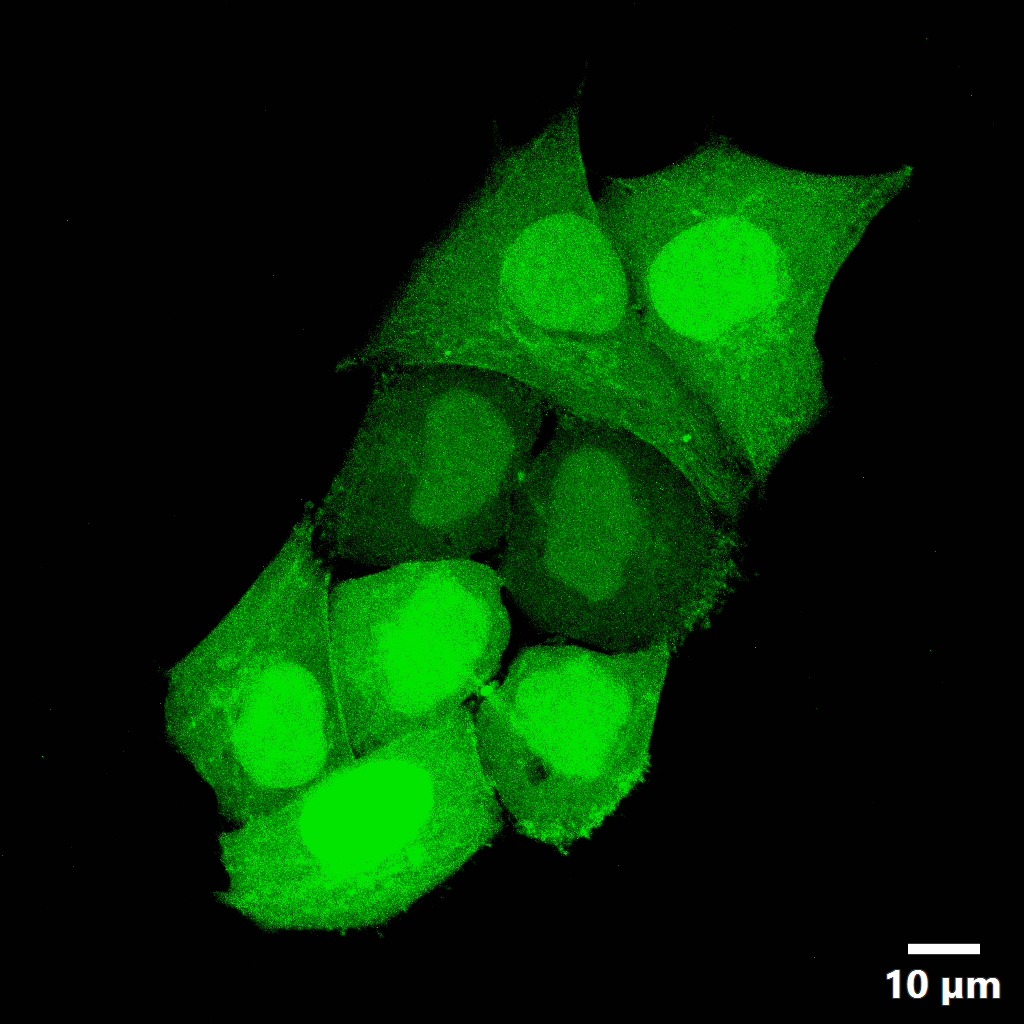

Supplement: Supplementary file 8 — Source data Fig. 6 [file 44319_2024_204_MOESM8_ESM.zip › Figure 6/6F/GFP-control (IF images)/GFP-control.tif]

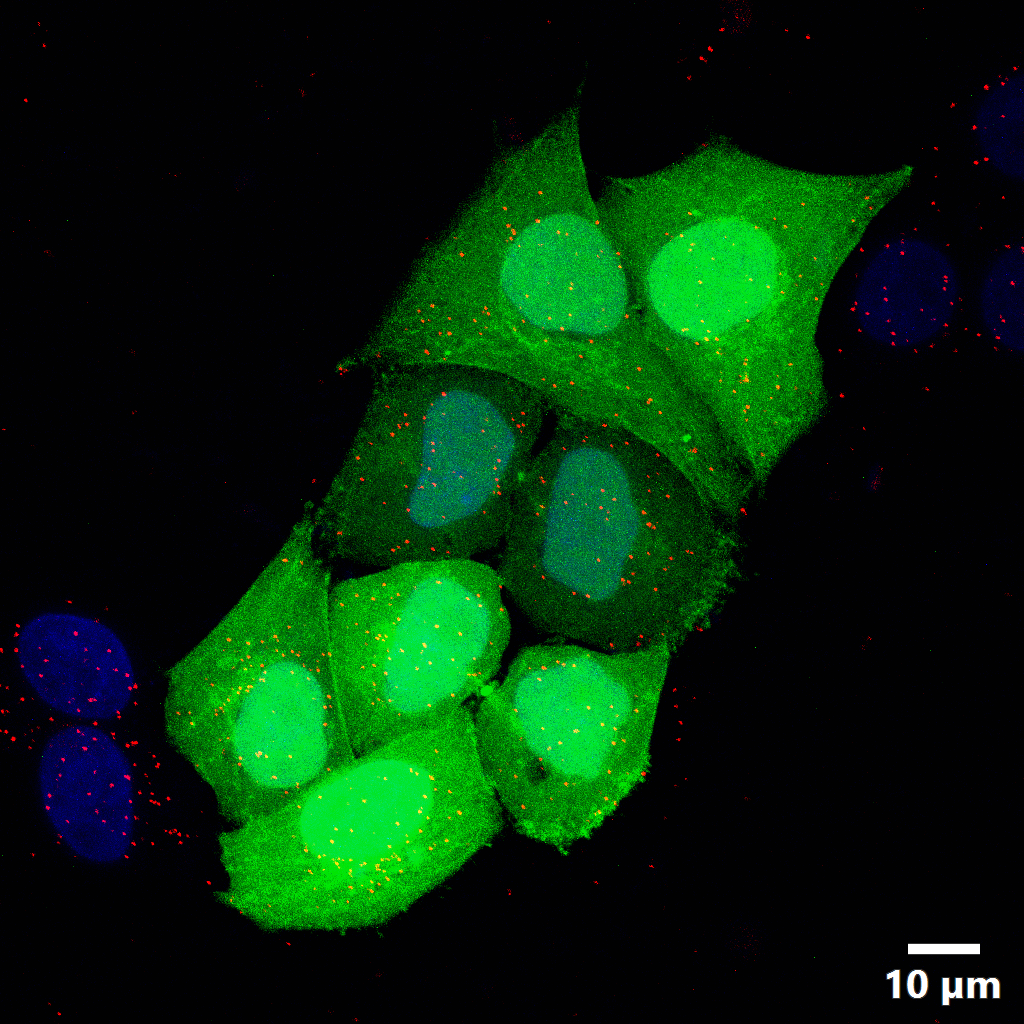

Supplement: Supplementary file 8 — Source data Fig. 6 [file 44319_2024_204_MOESM8_ESM.zip › Figure 6/6F/GFP-control (IF images)/Merge.tif]

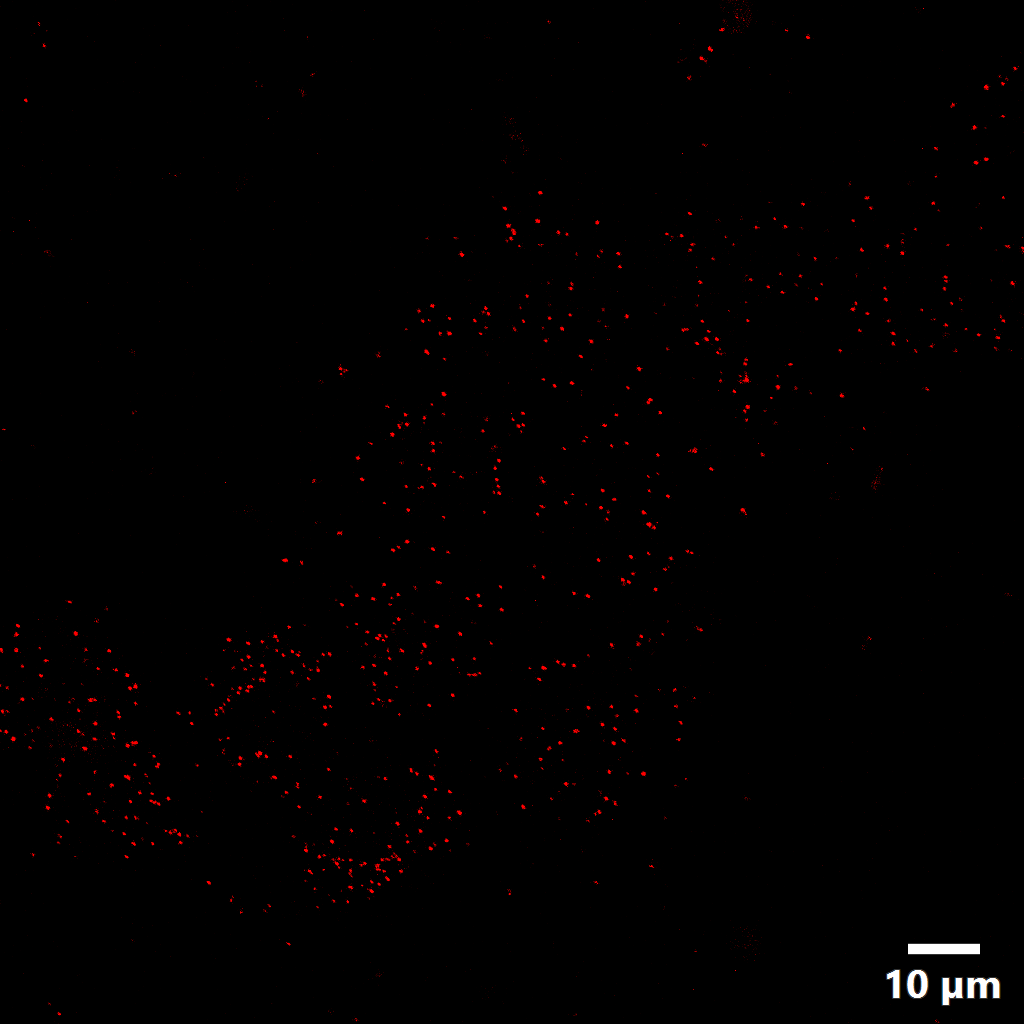

Supplement: Supplementary file 8 — Source data Fig. 6 [file 44319_2024_204_MOESM8_ESM.zip › Figure 6/6F/GFP-control (IF images)/PLA (VAPB+PTPIP51).tif]

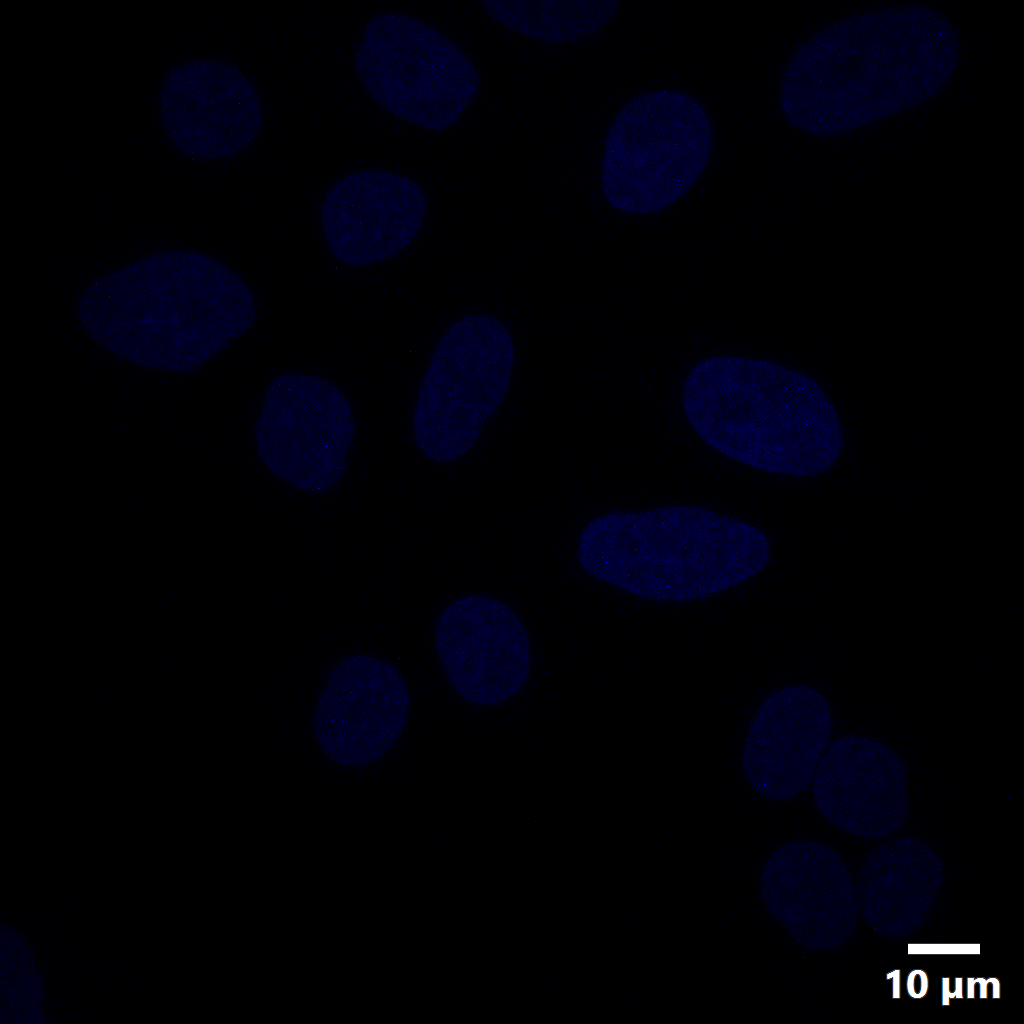

Supplement: Supplementary file 8 — Source data Fig. 6 [file 44319_2024_204_MOESM8_ESM.zip › Figure 6/6F/GFP-Nup358-MC1 (IF images)/DAPI.tif]

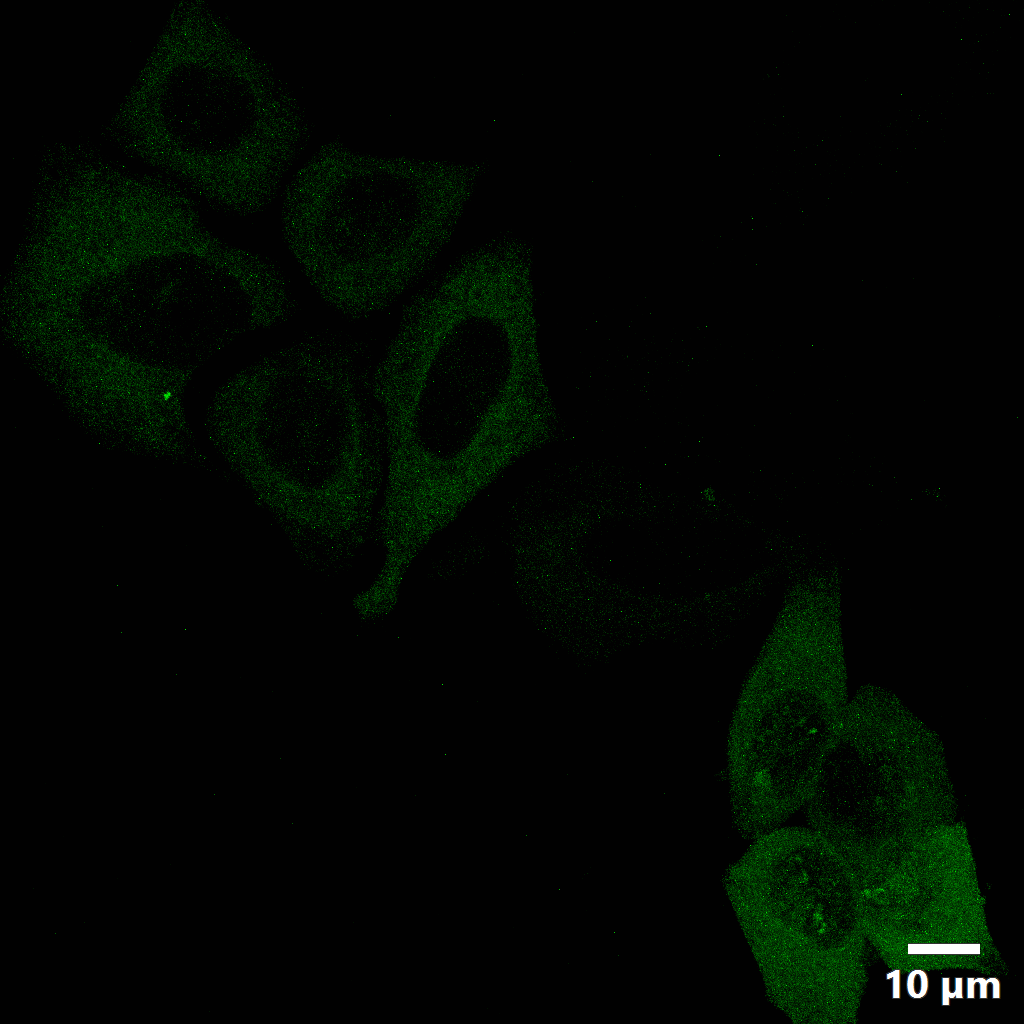

Supplement: Supplementary file 8 — Source data Fig. 6 [file 44319_2024_204_MOESM8_ESM.zip › Figure 6/6F/GFP-Nup358-MC1 (IF images)/GFP-Nup358-MC1.tif]

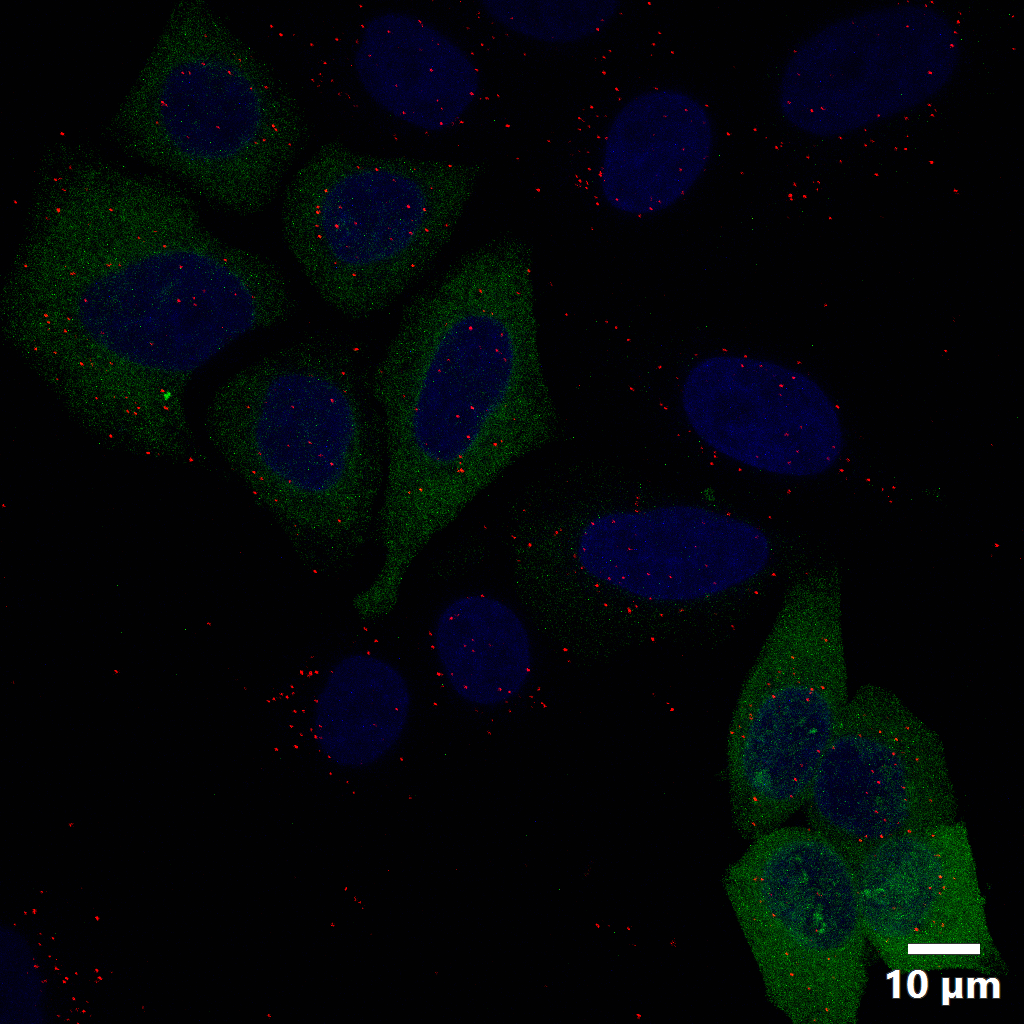

Supplement: Supplementary file 8 — Source data Fig. 6 [file 44319_2024_204_MOESM8_ESM.zip › Figure 6/6F/GFP-Nup358-MC1 (IF images)/Merge.tif]

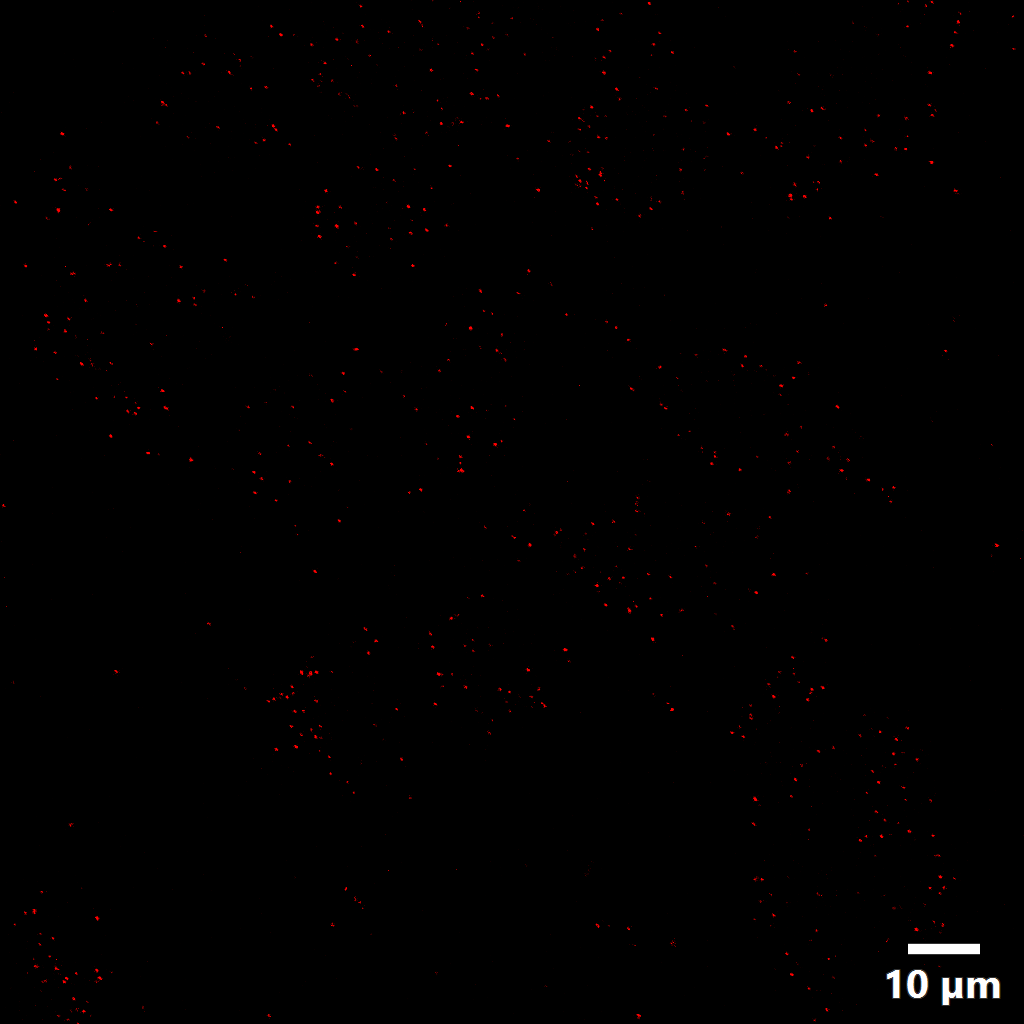

Supplement: Supplementary file 8 — Source data Fig. 6 [file 44319_2024_204_MOESM8_ESM.zip › Figure 6/6F/GFP-Nup358-MC1 (IF images)/PLA (VAPB+PTPIP51).tif]
