# Supplementary material for: Nup358 restricts ER-mitochondria connectivity by modulating mTORC2/Akt/GSK3β signalling
Source: EMBO Rep. 2024 Jul 18;25(10):14. doi: 10.1038/s44319-024-00204-8 (PMC11466962; doi:10.1038/s44319-024-00204-8)
Supplement: Supplementary file 9 — Expanded View Figures [file 44319_2024_204_MOESM9_ESM.pdf]

Expanded View Figures

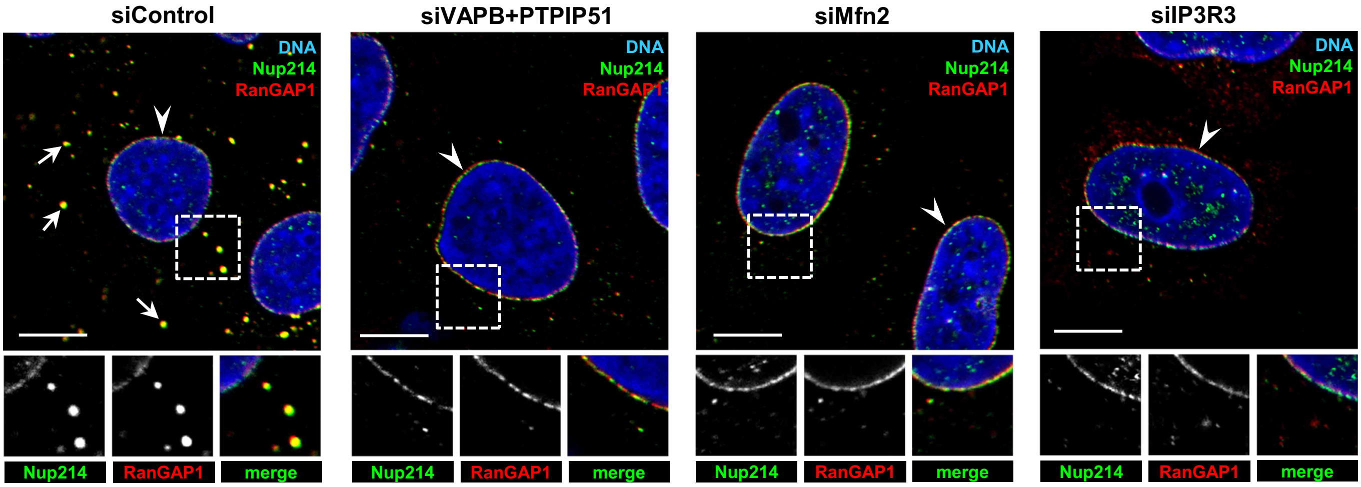

Figure EV1. ERMCS integrity is important for AL assembly and/or stability.

Depletion of ERMCS proteins affects AL assembly. HeLa cells were treated with indicated siRNAs and the AL integrity was monitored by presence of cytoplasmic Nup214 (green) and RanGAP1 (red). Here RanGAP1 was used as a surrogate for Nup358, as RanGAP1 is known as a strong binding partner that colocalizes with Nup358 at the nuclear envelope and AL. Number of cytoplasmic puncta, which represent AL (shown in arrows), are significantly reduced when ERMCS proteins are depleted as compared to control cells. Under the same conditions, the NE staining of nucleoporins (arrowheads) was largely unaffected. Scale bar, 10 µm.

 © The Author(s)

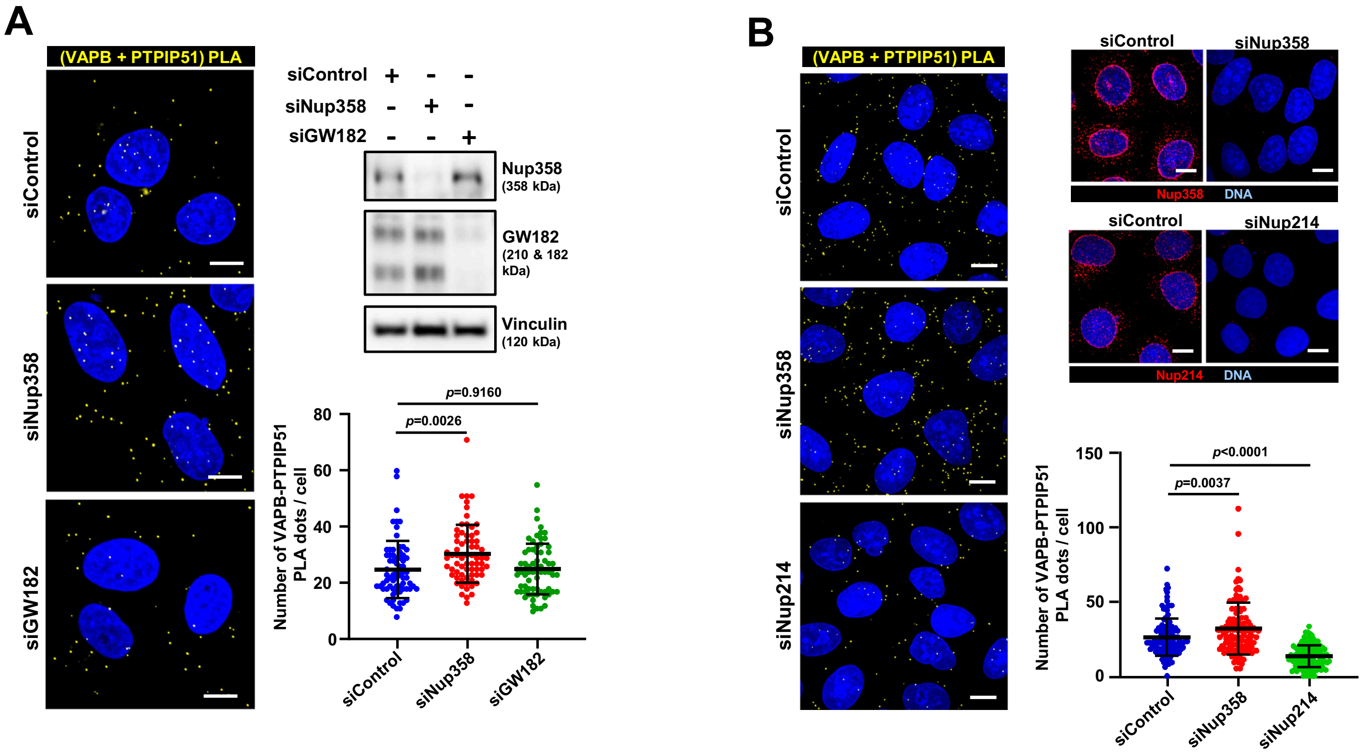

Figure EV2. Interfering with miRNA pathway does not affect ERMCSs.

(**A**) Depletion of GW182 does not affect contacts between ER and mitochondria. HeLa cells were treated with indicated siRNAs and the intactness of ERMCS was monitored in situ by PLA using VAPB and PTPIP51 antibodies. Left: Representative microscopic images showing PLA puncta (yellow) under the indicated conditions. DNA was stained with Hoechst 33342 (blue). Scale bar, 10 µm. Right top: Depletion of indicated proteins was confirmed by western blotting. Vinculin was used as loading control. Right bottom: Quantitative data showing number of PLA dots per cell, derived from indicated conditions ($n = 70$ cells for siControl; 69 cells for siNup358 and 71 cells for siGW182 from 3 independent experiments). Data are mean ± SD, unpaired Student's *t* test. *P* values are indicated. (**B**) Depletion of Nup214 reduces contacts between ER and mitochondria. HeLa cells were treated with indicated siRNAs and the ERMCS were monitored in situ by PLA using VAPB and PTPIP51 antibodies. Left: Representative microscopic images showing PLA puncta (yellow) under the indicated conditions. DNA was stained with Hoechst 33342 (blue). Scale bar, 10 µm. Right top: Depletion of indicated proteins was confirmed by immunostaining with the indicated antibodies (red). Right bottom: Quantitative data showing number of PLA dots per cell, derived from indicated conditions ($n = 127$ cells for siControl; 106 cells for siNup358 and 116 cells for siNup214 from 3 independent experiments). Data are mean ± SD, unpaired Student's *t* test. *P* values are indicated.

© The Author(s)

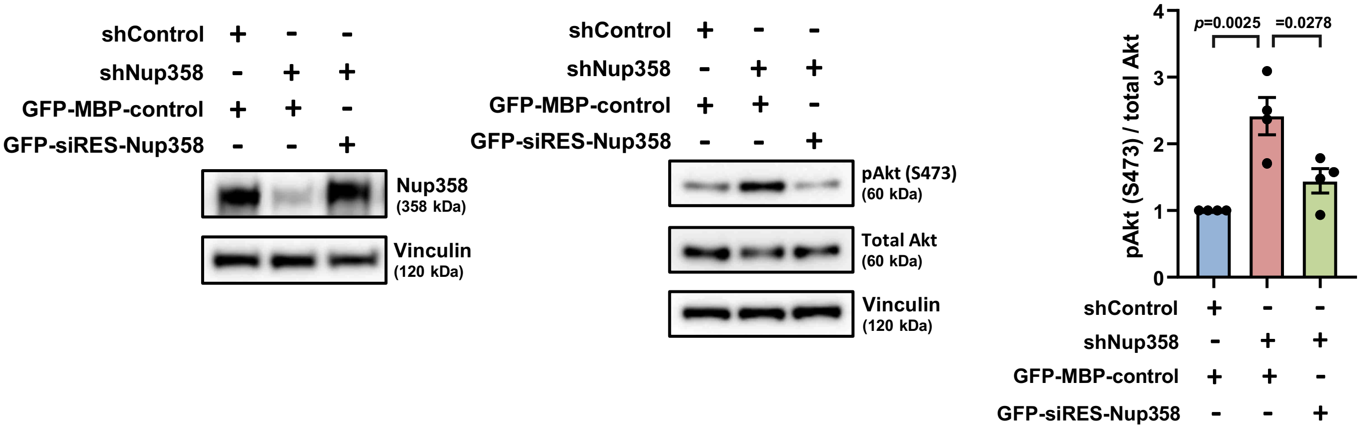

Figure EV3. Ectopic expression of Nup358 rescues the increased mTORC2/Akt activity in Nup358-deficient cells.

Stable HEK293T cells with Inducible short hairpin RNA (shRNA) for control (shControl) or Nup358 (shNp358) were initially induced with doxycycline. Later the cells were transfected with GFP-MBP-control or GFP-siRNA-resistant (siRES)-Nup358 construct as indicated. Left: Expression levels of Nup358 in the indicated samples were monitored by western blotting with Nup358 antibodies. Vinculin was used as loading control. Middle: Levels of indicated proteins were assessed by western blotting. Vinculin was loading control. Right: Quantitative representation depicting relative levels of pAkt normalized to total Akt ($n = 4$ independent experiments) under the indicated conditions. Data are mean ± SEM, Student's t test. P values are indicated.

 © The Author(s)

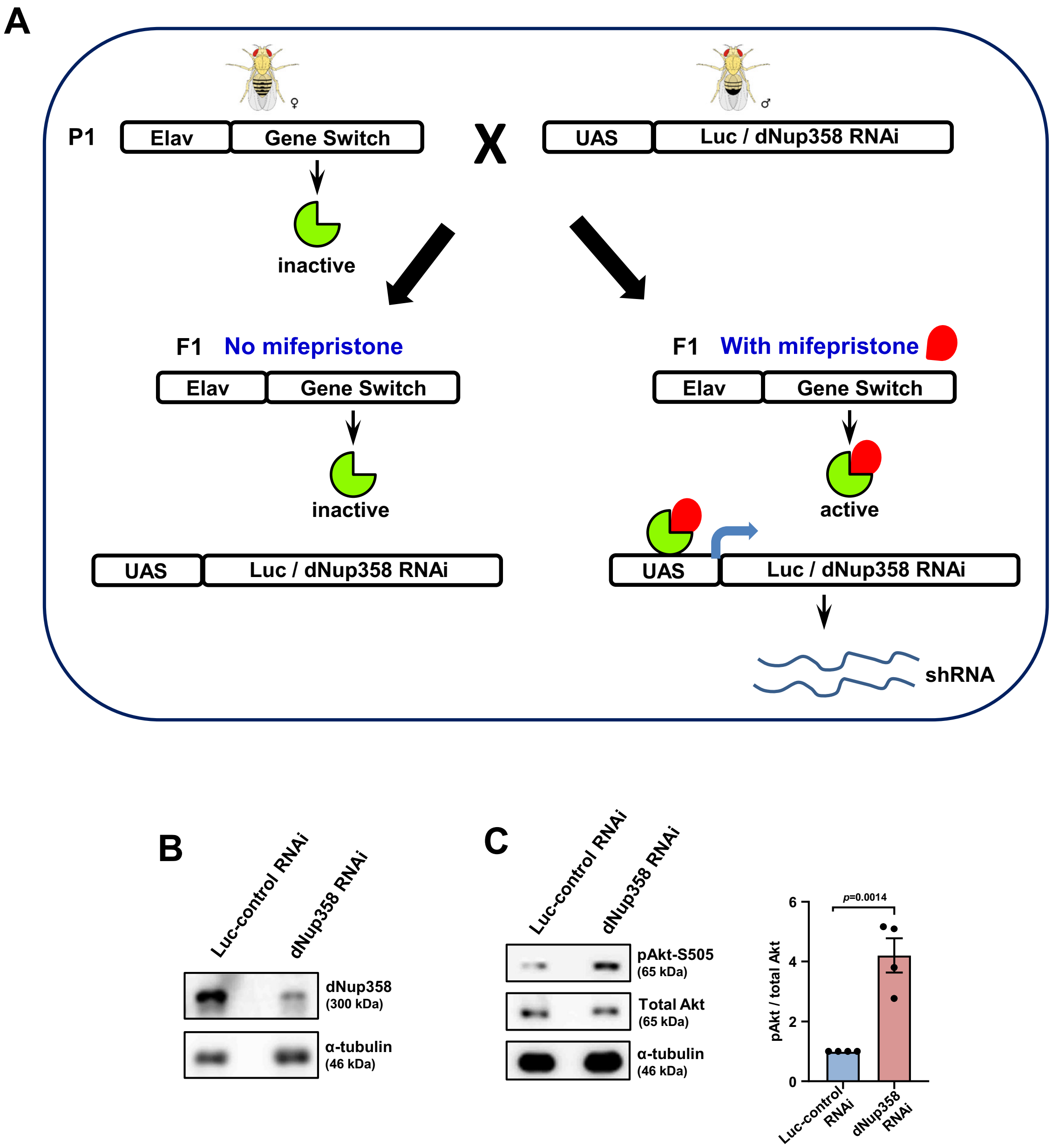

Figure EV4. Conserved regulatory role of Nup358 in restricting mTORC2/Akt activation in Drosophila.

(**A**) A schematics showing how F1 progeny was obtained by crossing parental (P) lines as indicated. The adult Drosophila flies (F1) were treated with 500 μM mifepristone (RU486) for 72 h to induce control shRNA (Luciferase, Luc) or dNup358-specific shRNA. (**B**) Brains lysates were assessed for dNup358 knockdown by western blotting. α-tubulin was used as loading control. (**C**) Left: Lysates were analysed for pAkt (S505) levels by western blotting. α-tubulin was used as loading control. Right: Quantitative data showing the levels of pAkt (normalized to total Akt) under indicated conditions ($n = 4$ independent experiments). Data are mean ± SEM, unpaired Student's t test. P value is indicated.

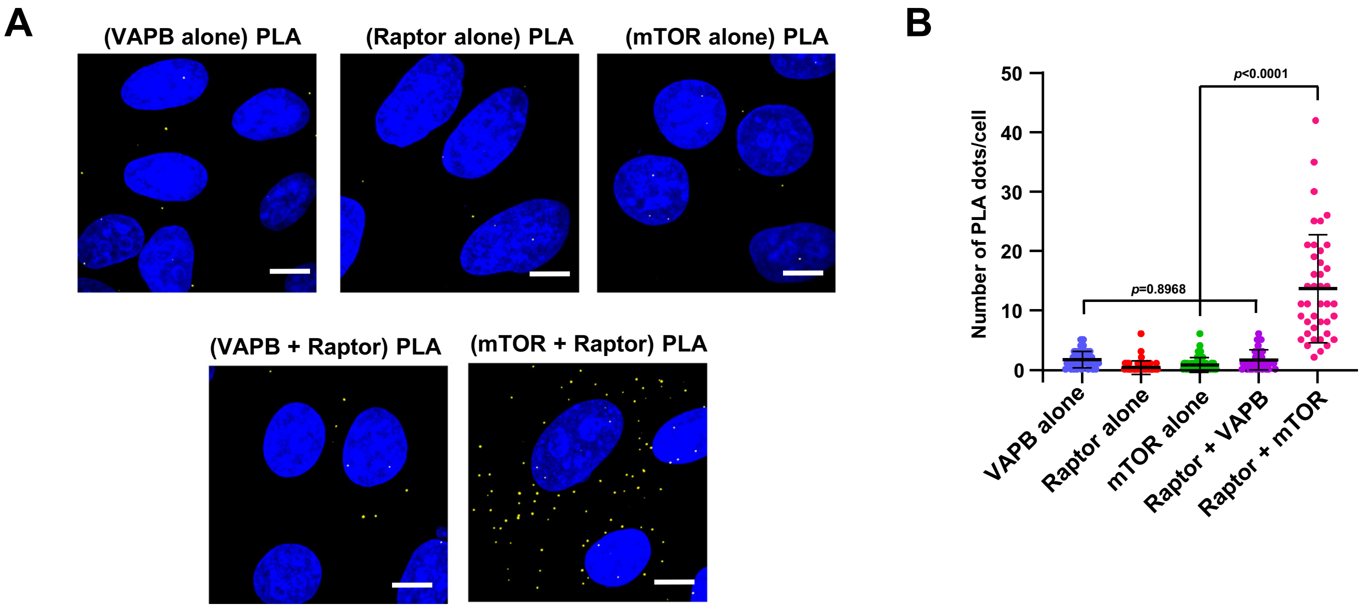

Figure EV5. VAPB does not interact with the mTORC1-specific subunit Raptor.

(**A**) HeLa cells were subjected to PLA (yellow dots) for detecting the VAPB-Raptor interaction and mTOR-Raptor (positive control) interaction, with individual antibody as negative controls. DNA was stained with Hoechst 33342 (blue). Scale bar, 10 µm. (**B**) Quantitation of PLA dots per cell (*n* = 56 cells for VAPB alone; 41 cells for Raptor alone; 57 cells for mTOR alone; 48 cells for VAPB-Raptor interaction; 41 cells for VAPB–mTOR interaction; from a single experiment). Data are mean ± SD, unpaired Student's *t* test. *P* values are indicated.

 © The Author(s)
